# Alzheimer’s Disease Immunotherapy and Mimetic Peptide Design for Drug Development: Mutation Screening, Molecular Dynamics, and a Quantum Biochemistry Approach Focusing on Aducanumab::Aβ2–7 Binding Affinity

**DOI:** 10.1021/acschemneuro.4c00453

**Published:** 2024-09-20

**Authors:** Victor L. B. França, Eveline M. Bezerra, Roner F. da Costa, Hernandes F. Carvalho, Valder N. Freire, Geanne Matos

**Affiliations:** † Department of Physiology and Pharmacology, Federal University of Ceará, 60430-270 Fortaleza, Ceará, Brazil; ‡ Department of Sciences, Mathematics and Statistics, Federal Rural University of Semi-Arid (UFERSA), 59625-900 Mossoró, RN, Brazil; § Department of Structural and Functional Biology, Institute of Biology, State University of Campinas, 13083-864 Campinas, São Paulo, Brazil; ∥ Department of Physics, Federal University of Ceará, 60430-270 Fortaleza, Ceará, Brazil

**Keywords:** computational biology, molecular dynamics, aducanumab affinity, monoclonal antibody

## Abstract

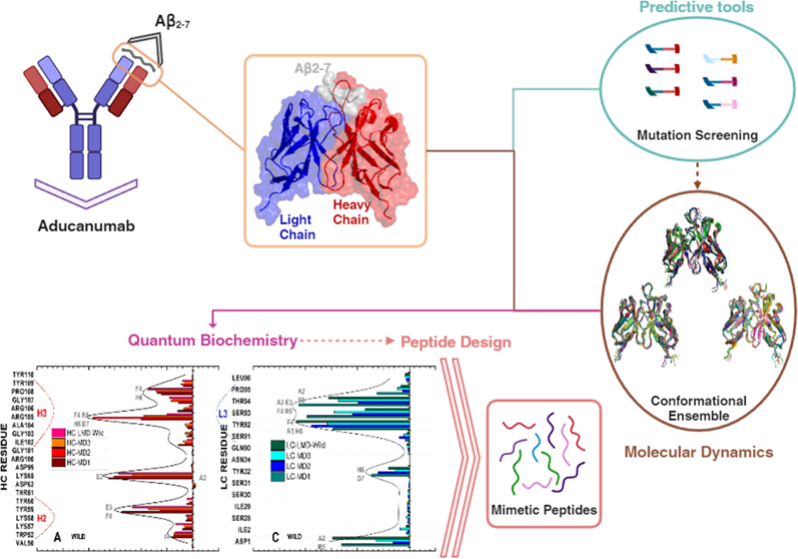

Seven treatments are approved for Alzheimer’s disease, but five of them only relieve symptoms and do not alter the course of the disease. Aducanumab (Adu) and lecanemab are novel disease-modifying antiamyloid-β (Aβ) human monoclonal antibodies that specifically target the pathophysiology of Alzheimer’s disease (AD) and were recently approved for its treatment. However, their administration is associated with serious side effects, and their use is limited to early stages of the disease. Therefore, drug discovery remains of great importance in AD research. To gain new insights into the development of novel drugs for Alzheimer’s disease, a combination of techniques was employed, including mutation screening, molecular dynamics, and quantum biochemistry. These were used to outline the interfacial interactions of the Aducanumab::Aβ_2–7_ complex. Our analysis identified critical stabilizing contacts, revealing up to 40% variation in the affinity of the Adu chains for Aβ_2–7_ depending on the conformation outlined. Remarkably, two complementarity determining regions (CDRs) of the Adu heavy chain (HCDR3 and HCDR2) and one CDR of the Adu light chain (LCDR3) accounted for approximately 77% of the affinity of Adu for Aβ_2–7_, confirming their critical role in epitope recognition. A single mutation, originally reported to have the potential to increase the affinity of Adu for Aβ_2–7_, was shown to decrease its structural stability without increasing the overall binding affinity. Mimetic peptides that have the potential to inhibit Aβ aggregation were designed by using computational outcomes. Our results support the use of these peptides as promising drugs with great potential as inhibitors of Aβ aggregation.

## Introduction

Alzheimer’s disease (AD) is directly responsible for 60–80% of cases of dementia in older individuals.^1^ Dementia refers to a significant loss of cognitive abilities beyond the natural neurodegenerative effects of aging.^1^ As life expectancy has increased over the past centuries, age-related diseases such as cancer, atherosclerosis, diabetes, and Alzheimer’s disease have become a growing concern.^2−4^ Although advanced age is a risk factor for these diseases, it is particularly critical for AD, where age is the greatest risk factor, surpassing genetics, and family history.^1^ It was estimated that healthcare costs associated with AD exceeded $345 billion in the United States in 2023, which is twice as much as the cost 10 years ago.^1,5^ This intensification in healthcare costs is related to the increase in life expectancy and aging, which is the greatest risk factor for AD development. The progression of AD is usually divided into three phases: preclinical AD, mild cognitive impairment due to AD, and dementia due to AD. This disease progresses slowly, resulting in the destruction of neurons, memory impairment, and decreased physical function.^5^ Decades of research on this neurodegenerative disease have revealed that cognitive decline is caused by a variety of pathological processes, including excessive extracellular aggregation of amyloid-β (Aβ), intracellular neurofibrillary tangles formed by hyperphosphorylation of tau protein, cholinergic dysfunction, excessive glutamatergic stimulation, oxidative stress, and neuroinflammation.^6−11^

Although AD has a complex pathophysiology, two hypotheses have gained significant evidence: the amyloid cascade hypothesis (ACH)^12−14^ and the tau hyperphosphorylation hypothesis (THH).^15−17^ The amyloidogenic pathway results from the sequential cleavage of the amyloid precursor protein (APP) by β-secretase and γ-secretase. β-Secretase cleaves at the N-terminus of APP, while γ-secretase cleaves at its intramembranous domain. The products of β-secretase-mediated cleavage are APP-β and a membrane-bound fragment, which is the target of γ-secretase. The γ-secretase enzyme is responsible for releasing C-terminal fragments and Aβ peptides that contain 38, 40, or 42 amino acid residues.^18,19^ These Aβ peptides become neurotoxic upon translocation to the extracellular cell and aggregate, progressively forming oligomers, protofibrils, and mature fibrils.^20^ Some evidence suggest these Aβ aggregates accumulate because the health balance between production and clearance of Aβ is dysregulated in the brain of AD patients.^21,22^ The THH is based on an imbalance in the degree of tau phosphorylation, which is 3–4 times higher than that found in healthy individuals.^23,24^ These filaments undergo further aggregation and thickening. In many cases, pathologically hyperphosphorylated tau protein’s ability to bind to tubulin is reduced, leading to a microtubule formation impairment.^23,24^

Although many efforts are directed toward finding a cure for Alzheimer’s disease, the available treatments have limitations. Five of seven AD treatments approved by the Food and Drug Administration (FDA) can improve symptoms without changing the course of the disease.^1^ These treatments are based on acetylcholinesterase inhibitors (donepezil, rivastigmine, galantamine), a glutamate receptor antagonist (memantine), and a combination of memantine and donepezil.^19^ Although these drugs are commonly used in the treatment of Alzheimer’s disease, they cannot interrupt neurodegeneration.^19^ As a result, numerous clinical trials have been conducted to evaluate potential disease-modifying therapies.^25,26^

As a result of numerous efforts, two monoclonal antibodies (mAbs) have been approved by the FDA: Aducanumab (Adu)^27^ and Lecanemab (Lec).^28^ Adu and Lec were found to have the strongest affinities for Aβ fibrils and protofibrils, respectively.^29^ These affinities lead to the destabilization of these structures and activate immune system-mediated clearance of Aβ aggregates.^29−31^ In addition, Donanemab’s clinical trial results recently led to an FDA approval application whose acceptance is expected soon.^32^ Currently, these drugs are only approved to treat patients with mild cognitive impairment due to AD and mild AD.^19^ Additionally, the clinical efficacy of Aducanumab lacks solid evidence,^33^ and the administration of Adu and Lec has resulted in significant occurrences of amyloid-related imaging abnormalities due to edema or effusion, leading to extensive debate in recent years.^34−38^ Therefore, it is widely agreed that drug discovery remains of major importance in Alzheimer’s disease research.

AD immunotherapy research aims to provide a disease-modifying therapy that improves cognitive functions by clearing aggregates of Aβ and impairing oligomerization and fibrillation in the Aβ cascade.^31^ Although many anti-Aβ mAbs have failed in clinical trials, Adu and Lec have resulted in a reduced decline in cognitive abilities.^26^ It is well-established that antibodies directed toward the N-terminus of Aβ elicit superior clinical responses compared to those that bind to central segments or the C-terminus, such as Solanezumab and Crenezumab.^39^ Compared to other anti-Aβ monoclonal antibodies, Adu has a unique inhibitory mechanism of Aβ aggregation, selectively reducing the secondary nucleation rate of Aβ42 resulting in a significant reduction of Aβ oligomers.^31^ The FDA’s approval of Adu is a historic landmark, but it has also raised concerns and highlighted the fact that a definitive treatment for Alzheimer’s disease is still far from being developed. Although there are some contradictions, this monoclonal antibody may lead to better cognitive and clinical improvements compared to Donanemab and Lecanemab, which are also Aβ cleaners.^40^

**Figure 1:**
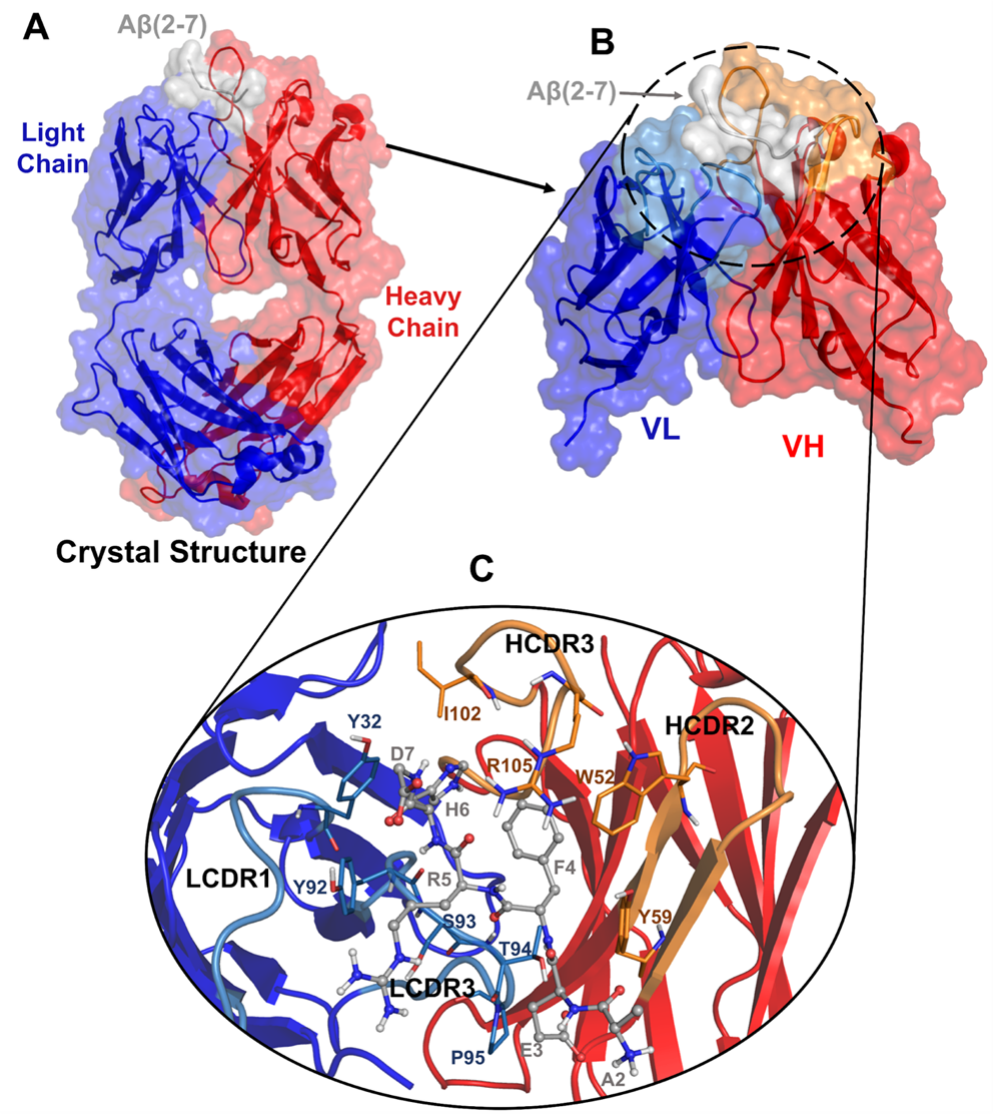
Overview of Aducanumab::Aβ_2–7_ crystallographic structure with HC’s missing residues modeled. (A) Crystallographic structure of Aducanumab::Aβ_2–7_ represented in the cartoon with HC, LC, and Aβ_2–7_ colored in red, blue, and gray. (B) Portion subjected to molecular dynamics simulations corresponding to the variable fragment heavy chain (VH) and variable fragment light chain (VL) bound to Aβ_2–7_. (C) Illustration of the main contacts on the Aducanumab::Aβ_2–7_ surface.

Aducanumab is a recombinant human immunoglobulin (IgG1) that can cross the blood–brain barrier and promote the clearance of Ab aggregates.^41^ It has a distinctive selectivity for soluble Ab oligomers and insoluble Aβ fibrils.^41^ A comparison with gantenerumab, another anti-Aβ mAb, illustrates this distinct selectivity: Adu has Aβ monomer binding affinity that is approximately 100-fold lower than that of gantenerumab, while both antibodies have similar affinities for AB aggregates.^42^ Clinical trials have shown that Aβ cleaning mediated by Adu occurs in a dose- and time-dependent manner.^41^ In addition, Adu is the only monoclonal antibody with FDA approval to treat Alzheimer’s disease and a resolved and published three-dimensional structure (see Figure 1).^26,42^ The elucidated cocrystal structure demonstrates that Adu interacts with the N-terminus of Aβ, which adopts an extended conformation during binding.^42^ Based on structural and biochemical analyses, the determinant regions responsible for recognizing the epitope formed by Aβ amino acids 3 to 7 are HCDR2, HCDR3, and LCDR1,^42^ see Figure 1.

From a pharmaceutical viewpoint, it is critical to understand the atomic interactions that are responsible for positive clinical responses. This comprehension is essential for the development and discovery of drugs that can eliminate Aβ aggregates and prevent oligomer/fibril formation. Therefore, designing such a pharmaceutical drug necessitates comprehensive knowledge not only of the Adu::Aβ structure but also of its binding mechanism including dynamics and affinity characterization. The structural data of anti-Aβ mAbs has provided useful information in AD research.^42−45^ Some studies have outlined mAb::Aβ complexes through molecular dynamics (MD) simulations to gain new insights into the binding mechanism.^46−48^ On the other hand, quantum biochemistry is a powerful computational method that has been used to study biological complexes^49^ related to various diseases, including schizophrenia,^50^ cancer,^51,52^ arterial hypertension,^53^ and COVID-19.^54^ Thus, the implementation of quantum biochemistry coupled with MD is a reliable approach to describe the surface interactions of Alzheimer’s drugs as Aducanumab, the only anti-Aβ drug with both FDA approval and a solved three-dimensional structure.

This work aims to provide a detailed molecular-level description of the surface interactions between Aducanumab and Aβ_2–7_ (Figure 1). In addition, we investigate mutations that could enhance Adu’s binding affinity to this epitope, and also we design synthetic peptides with a mechanism of action similar to Aducanumab, which can be cheaply synthesized and improved. To accomplish this, we employ computational techniques based on molecular mechanics and quantum mechanics. To the best of our knowledge, this is the first attempt to apply quantum mechanics to study/design drugs in the realm of Alzheimer’s disease immunotherapy treatment. In particular, the computational results allowed the design of mimetic peptides that have potential use in the treatment of Alzheimer’s disease.

## Results and Discussion

### Screening of Mutations

A set of 23 Adu’s amino acid mutations with potential to increase the affinity between aducanumab and Aβ_2–7_ was initially obtained. Most of them are LC’s mutations, such as LC-SER91 and LC-SER30, which had the highest numbers of potential single amino acid substitutions identified (Table 1). However, only the LC-S91Y mutation presented three favorable predictive measurements of ΔΔ*G*_Bind_, ΔΔ*G*_Affinity_, and ΔΔ*G*_Stability_ using different methods (Table 1). This initial screening provides a rationale for selecting S91Y as the most promising mutation based on two suspected increases in Adu::Aβ_2–7_ binding affinity and an indication of improvement in complex stability. Since previous reports have shown that antibody mutations can improve affinity and specificity for epitopes,^55,56^ this screening was conducted with the aim of identifying potential mutations that could enhance the therapeutic properties of Aducanumab. Thus, the LC-S91Y mutation was inserted into the complex Adu::Aβ_2–7_ to perform a comparison between wild and mutated complexes in terms of structural stability and binding affinity data using robust computational techniques.

**Table 1:** Screening of Potential Mutations in Aducanumab’s Chains^a^

LC mutation^b^	ΔΔ*G*_bind_^b^ (kcal/mol)	ΔΔ*G*_affinity_^c^ (kcal/mol)	ΔΔ*G*_stability_^d^ (kcal/mol)	HC mutation^b^	ΔΔ*G*_bind_^b^ (kcal/mol)	ΔΔ*G*_affinity_^c^ (kcal/mol)	ΔΔ*G*_stability_^d^ (kcal/mol)
S91W	–0.43	0.39	0.25	R105H	–0.2	0.95	0.03
S91Y	–0.29	–1.85	0.46	G107F	–0.14	0.01	0.05
S30W	–0.25	0.83	–0.12	P108W	–0.12	0.76	–0.21
S91F	–0.25	0.18	0.46	P108M	–0.11	0.73	–0.71
S30G	–0.19	0.81	–0.32	R105F	–0.09	0.87	–0.09
S91I	–0.19	0.85	0.54	G107L	–0.09	0.04	0.37
T94W	–0.14	0.49	0.03	R105L	–0.06	0.85	–0.05
Y32W	–0.12	0.95	–0.25	P108Y	–0.04	–0.27	–0.23
S93M	–0.1	1.16	–0.26				
S91V	–0.09	0.61	0.14				
S30F	–0.07	0.88	–0.18				
S91H	–0.07	–0.24	–0.11				
S30Y	–0.06	0.96	–0.01				
S30H	–0.02	0.93	–0.46				
S93L	–0.02	1.16	–0.2				

a The only mutation with three predictive adequate parameters (and its parameters) are underlined.

b List of HC and LC mutations provided by BEATMUSIC 1.0 (http://babylone.ulb.ac.be/beatmusic/index.php) with its respectively increase in the affinity measured in kcal/mol.

c A second measurement of binding affinity variation (in kcal/mol) assessed in MutaBind2 (https://lilab.jysw.suda.edu.cn/research/mutabind2/research/mutabind2/).

d The structural stability variation (in kcal/mol) resultant from mutation calculated by DeepDDG (https://protein.org.cn/ddg.html).

### Structural Variations and Fluctuations of Aducanumab::Aβ_2–7_ Complex

**Figure 2:**
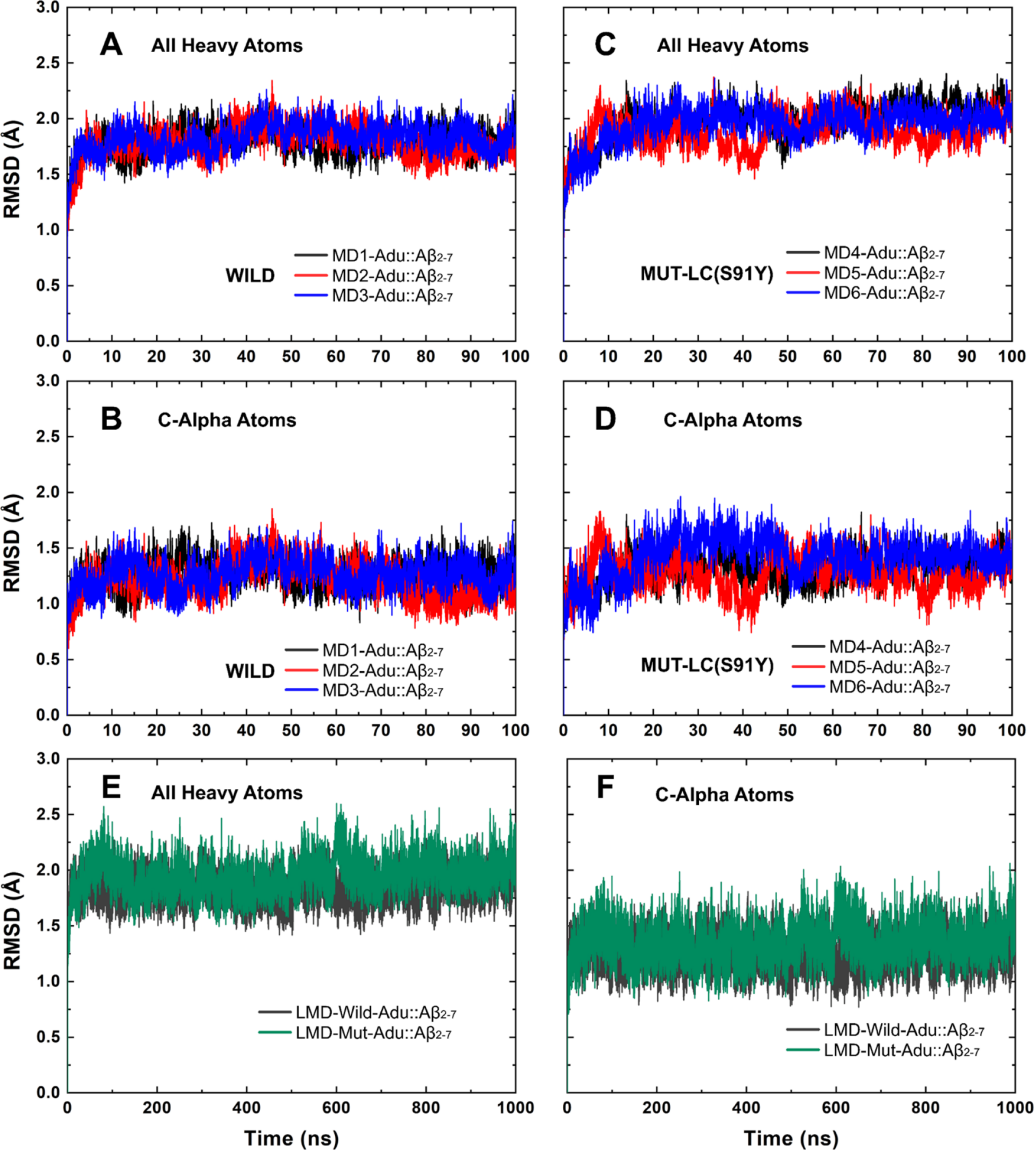
RMSD values vs simulation time. RMSD values of the wild complex within short MDs and measurements based on (A) all heavy atoms and (B) C-α atoms. The RMSD variation of mutated aducanumab::Aβ_2–7_ for (C) all heavy atoms and (D) C-α atoms in short MDs. The RMSD measurements considered (E) all heavy atoms and (F) C-α atoms in long MDs.

**Figure 3:**
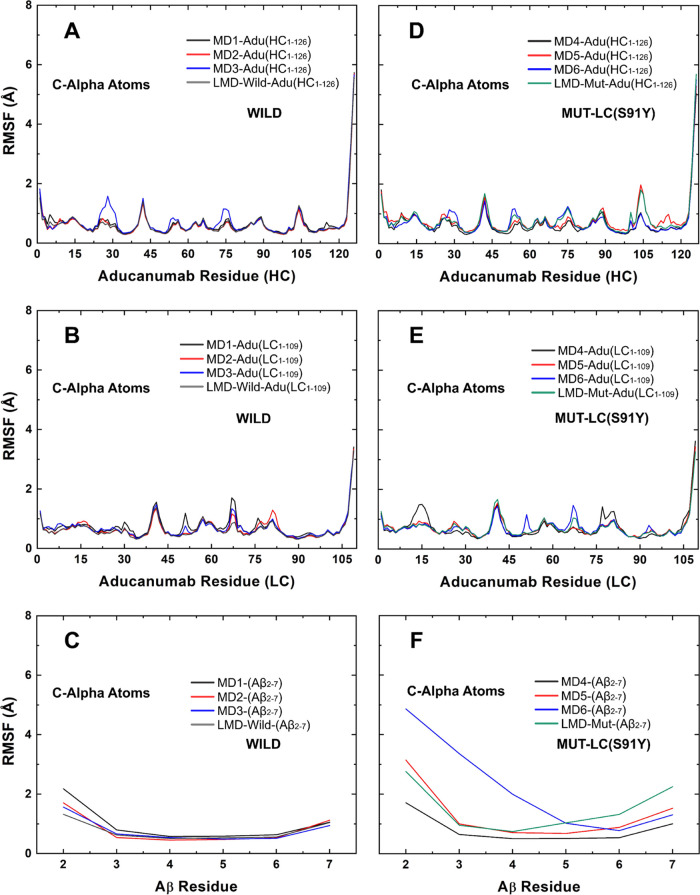
RMSF values calculated from short (MD1-MD6) and long (LMD-Wild and LMD-Mut) simulations of (A–C) wild and (D–F) mutated aducanumab::Aβ_2–7_ based on C-α atoms.

For the simulation time interval of 5–100 ns in the short molecular dynamics, the average root-mean-square deviation (RMSD) fluctuations of all heavy atoms (c-α-ΔRMSD) were 0.83 (0.95 Å) and 0.91 (1.07 Å) for the wild and mutated complexes, respectively (Figure 2). The extended simulations also demonstrated that the mutated complex exhibited the highest ΔRMSD, with fluctuations slightly exceeding those observed in the short MD. The ΔRMSD values for these complexes, calculated using all heavy atoms (C-α), were 0.94 (1.05) and 1.17 (1.24) (Figure 2). The flexibility of aducanumab residues measured by root-mean-square fluctuations (RMSF) values revealed that this structure is predominantly rigid with RMSF < 1 Å, with minor segments having RMSF near 2 Å (Figure 3A,B). The Aβ_2–7_ residues showed a similar profile, with _Aβ_ALA2 as the only residue with RMSF near 2 Å in multiple replicas (Figure 3C). The only difference detected between wild and mutated Aducanumab chains was a minor increase in the level of fluctuations of residues close to that of _LC_TYR91 (Figure 3E).

Moreover, when compared to the wild complex, Aβ_2–7_ bound to mutated Adu showed higher fluctuations (Figure 3F). In comparison to the fluctuations observed in the Aβ_2–7_ fragment bound to the wild form of Adu (Figure 3C), the fluctuations in the entire Aβ_2–7_ fragment bound to the mutated form of Adu (Figure 3F) were found to be higher. Both short molecular dynamics replicas and long molecular dynamics (LMD) corroborated this structural hallmark (Figure 3). This instability occurs mainly in _Aβ_ALA2, _Aβ_GLU3, and _Aβ_PHE4 which showed RMSF > 2 Å in simulation MD6 (Figure 3F). However, a similar pattern was also observed in MD5 and the LMD of the mutated complex (LMD-Mut; Figure 3F). Interestingly, the comparison of RMSF measurements from 1 μs LMD simulations also revealed the highest structural fluctuations of the portion _Aβ_ARG5-_Aβ_ASP7 of Aβ_2–7_ bound to the mutated Adu (Figure 3).

The _Aβ_GLU3-_Aβ_HIS6 portion has RMSF values between 1 and 2 Å in the simulations of the unbound Aβ_2–7_ fragment (Figure S1), while the same portion has only values below 1 Å when bound to wild Adu (Figure 3C) and few values above 1 Å in the mutated complex (Figure 3F), confirming that this portion is critical for maintaining Aβ_2–7_ trapped on the aducanumab surface, as previously reported.^42^ For more information about the structural stability of unbound Aβ_2–7_ during simulations, see Figures S1 and S2. Moreover, _Aβ_ARG5 showed a distinct fluctuation pattern: the high fluctuation level in the side chain, while the main chain was rigid (Figure S3).

**Figure 4:**
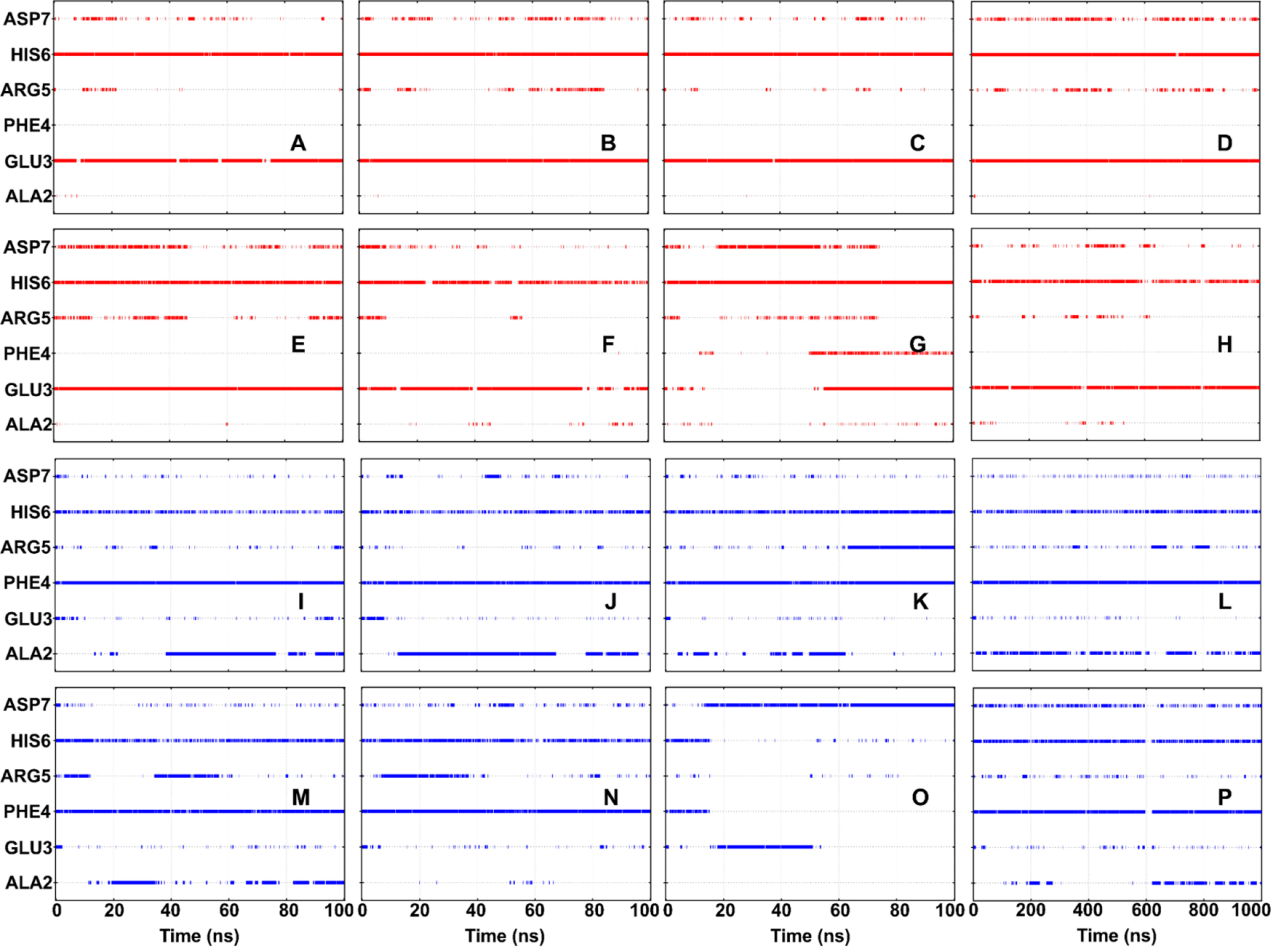
Hydrogen bond profile of the Aβ_2–7_ residues with the aducanumab (A–H) heavy chain and (I–P) light chain over the entire period of the simulations. Data of simulations (A, I) MD1, (B, J) MD2, (C, K) MD3, (D, L) LMD-Wild, (E, M) MD4, (F, N) MD5, (G, O) MD6, and (H, P) LMD-Mut are represented.

It was found that LC has more hydrogen bonds with Aβ_2–7_ than HC in both short and long simulations (Figures S4 and S5). _Aβ_GLU3 and _Aβ_HIS6 were the residues with the most conserved hydrogen bond contacts with HC (Figure 4), while _Aβ_PHE4 and _Aβ_HIS6 were predominantly responsible for these contacts with LC (Figure 4). Compared with simulations of the wild complex, the MD5′s and MD6′s hydrogen bond profiles (performed by Aβ_2–7_ during the simulations) showed that _Aβ_ALA2 stopped interacting with LC and started establishing strong interactions with HC (Figure 4). LMD simulations demonstrated that this phenomenon is a characteristic feature of mutated complex dynamics, occurring in a cyclic manner. Specifically, _Aβ_ALA2 forms and subsequently loses hydrogen bonds with LC, before reestablishing them at a later point in time (Figure 4). Moreover, the hydrogen bonds _Aβ_PHE4::LC and _Aβ_HIS6::LC were not conserved in MD6 after 20 ns (Figure 4). This dynamics profile suggests that the ability to maintain Aβ_2–7_ trapped and stable within the Aducanumab’s paratope was affected by the single amino acid substitution S91Y in the Aducanumab’s light chain. Despite these changes resulting from LC-S91Y, the mutated system remains as a stable complex and does not undergo extensive conformational changes and has the potential for spontaneous occurrence, as suggested by both its average RMSD values below 2 Å and the ΔRMSD of the order of 1 Å (Figure 2).

In contrast to the previous work, which performed simulations for complexes containing Aβ_2–7_, Aβ monomer, and Aβ oligomers,^47^ our efforts were focused on the crystallographic structure of Aducanumab::Aβ_2–7_. This Aβ fragment contains the Adu epitope Aβ_3–7,_^42^ which makes it a valuable structure for understanding surface interactions. The simulations demonstrated that Aducanumab undergoes minor conformational changes, which is consistent with the findings of Arndt et al. Differences in structural stability between Adu::Aβ_2–7_ and Mut-Adu::Aβ_2–7_ complexes can be inferred from the differences between their RMSD and RMSF values. The LC-S91Y mutation appears to cause a slight change in the structural stability of Adu::Aβ_2–7_. It is worth noting that some of the RMSF values obtained for the wild complex differ slightly from those reported by Frost and Zacharias.

Most simulations indicate that Aβ_2–7_ is rigid, but one simulation (MD6) of the mutated complex showed a high displacement of _Aβ_ALA2-_Aβ_PHE4, similar to the lack of stabilization in the N-terminus of Aβ_2–7_ of the crystallographic structure previously reported through MD simulation.^47^ Although the computational results did not reveal high RMSF values for the wild complex, a 500 ns simulation time of wild Adu::Aβ_2–7_ previously reported that this displacement in the N-terminus can be part of the recognition of Adu::Aβ_2–7._^47^ In view of this, performing this process in a faster mode can be an interesting feature, as is the case here with the mutated Adu. This modified antibody may potentially offer a novel binding way with the potential to disrupt Aβ aggregates, as previously proposed by distinct theoretical studies that evaluated mutations in proteins.^57−59^ The divergence between molecular dynamics conducted at varying time scales reiterates that simulation time significantly influences structural stability measurements. Additionally, the force field utilized by Frost and Zacarias differed from the one applied herein, which helped to elucidate why some structural behaviors observed here differed from those reported by them. Nevertheless, the structural descriptors RMSD and RMSF can suggest a minor propensity for Mut-Adu::Aβ_2–7_ to occur spontaneously, and additional assays are required to determine if LC-S91Y affects the stability and binding affinity of Aβ_2–7_. It is crucial to acknowledge that these structural descriptors (RMSD and RMSF) were employed to examine the LC-S91Y impact, drawing upon prior studies that effectively utilized theoretical methodologies in disparate biological systems.^60,61^

### Conformational Ensembles and Description of Nonbonded Interactions

**Figure 5:**
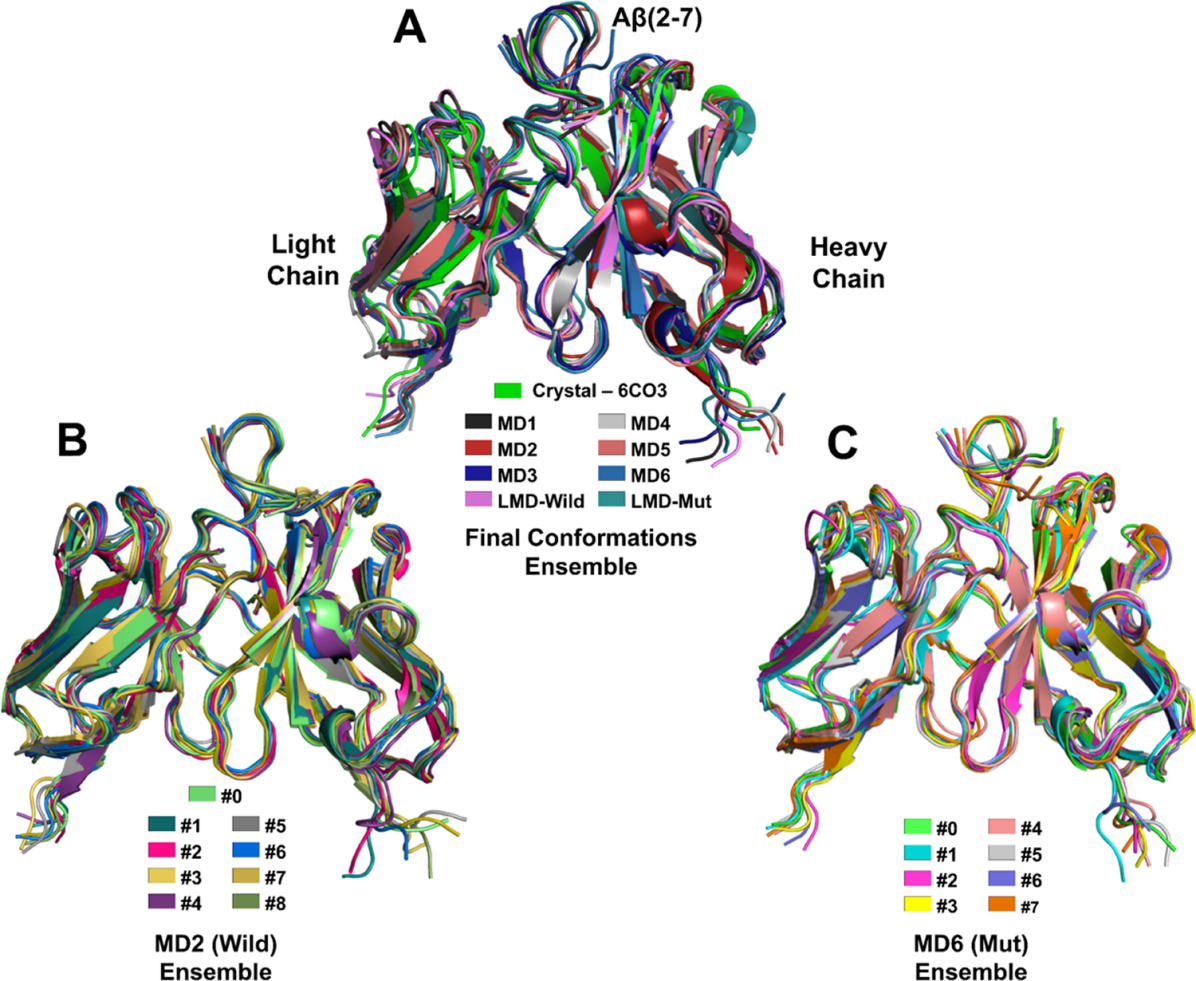
Structural alignment of conformational groups named the (A) final conformations (FC), (B) MD2 (Wild) ensemble, and (C) MD6 (Mut) ensemble.

The conformational ensembles under scrutiny consist of the final conformations of the wild complex (Wild-FC) named MD1, MD2, MD3, and LMD-Wild, and the final conformations of the mutated complex (Mut-FC) are named MD4, MD5, MD6, and LMD-Mutated (Figure 5A). Additionally, the representative conformations (RC) of the wild-type (MD2/Wild-RC) and mutated (MD6/Mut-RC) complexes were enumerated as #0–#8 (Figure 5B) and #0–#7 (Figure 5C), respectively. Among the simulations of the wild complex, MD2 exhibited the highest RMSD fluctuation, while MD6 showed the highest fluctuation among the mutated complex simulations (Figure 2). As a consequence (and aiming a high conformational flexibility between the representative conformations), the trajectories of these two simulations were used to generate the RC above-mentioned.

Compared to the crystallographic conformation, most of the final MD conformations showed a dislocation of _Aβ_ALA2 directed toward LC (Figure 5A), confirming the hydrogen bond profile that shows a strong interaction between _Aβ_ALA2 and LC beginning before 20 ns in MD1, MD2, MD3, MD4 and LMD-Wild (Figure 4). However, MD6 and Mut-RC revealed a distinct behavior where _Aβ_ALA2-_Aβ_PHE4 dislocated from LC to HC (Figure 5A–C).

**Figure 6:**
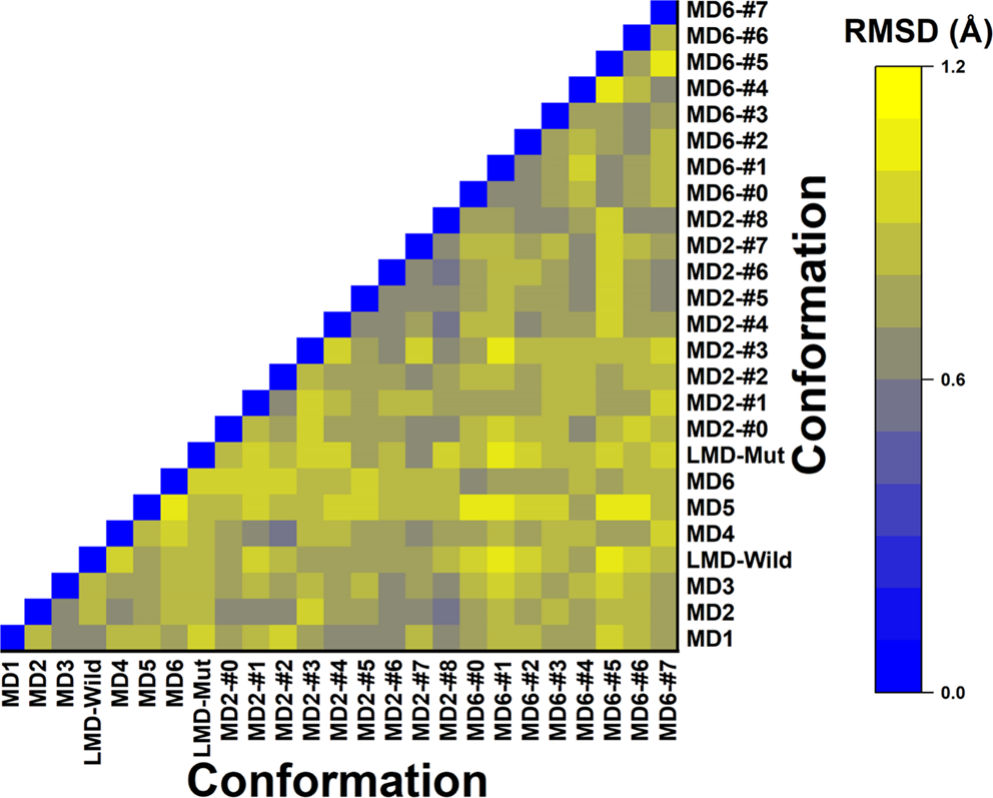
Heatmap of RMSD values between Aducanumab::Aβ_2–7_ conformations. The caption for the correlation of colors and RMSD values is on the right panel of the graph.

The conformational ensemble structures have RMSD values smaller than 1.2 Å from each other, including those provided by LMD (Figure 6). This structural similarity suggests that short molecular dynamics may be an appropriate approach to obtain Adu::Aβ_2–7_ binding states with energetic convergence. Although these RMSD values indicate high similarity, some conformations exhibit exclusive nonbonded interactions that are absent in others. For example, there are _LC_SER93(OG)::_Aβ_ALA2(N), _LC_TYR92(OH)::_Aβ_ARG5(NH1), _LC_TYR92(OH)::_Aβ_ARG5(NH2), _LC_TYR92(OH):: _Aβ_ASP7(OD2), _HC_LYS57(NZ)::_Aβ_GLU3(OE1), and _LC_GLN27(OE1)::_Aβ_ARG5(NH1) hydrogen bonds (Tables S1–S3). The high number of hydrophobic and hydrogen bond contacts conserved among many conformations confirms the rigidity of the Adu::Aβ_2–7_ complex (Tables S1–S3). To name a few, the hydrophobic interactions _HC_TRP52::_Aβ_PHE4, _HC_LYS65::GLU3, _HC_ILE102::_Aβ_HIS6, _HC_PRO108::_Aβ_HIS6, _LC_TYR92::_Aβ_ARG5, and _LC_SER93::_Aβ_PHE4 were detected at least 15 of the 23 Adu::Aβ_2–7_ conformations. Noteworthy, the hydrophobic interaction _HC_LYS65::_Aβ_GLU3 was not detected within MD6(Mut)-ensemble, suggesting that LC-S91Y directly affects this contact (Tables S1–S3). This mutation also resulted in the loss of three conserved hydrogen bonds: _LC_TYR92(O)::_Aβ_HIS6(N), _LC_THR94(OG1)::_Aβ_PHE4(N), and _LC_THR94(N)::_Aβ_PHE4(O) (Tables S1–S3). For more details about surface interactions about these interactions, see Figures S6–S9.

Most of the hydrophobic and hydrogen bond interactions detected here are in accordance with those previously reported by Arndt et al. This abundance of conserved interactions is also consistent with the small differences between conformations with all RMSDs measured within 1.20 Å. However, the conformational ensemble revealed some transient hydrogen bonds that were not previously reported, such as _HC_ARG105::_Aβ_ASP7, _HC_ASP1::_Aβ_ALA2, _LC_GLN27::_Aβ_ARG5, and _LC_TYR92:: _Aβ_ARG5. In agreement with previous reports, this clearly reveals the significance of employing a conformational ensemble approach within the context of molecular dynamics, even for rigid proteins.^62^

### The Aducanumab::Aβ_2–7_ Quantum Biochemistry

**Figure 7:**
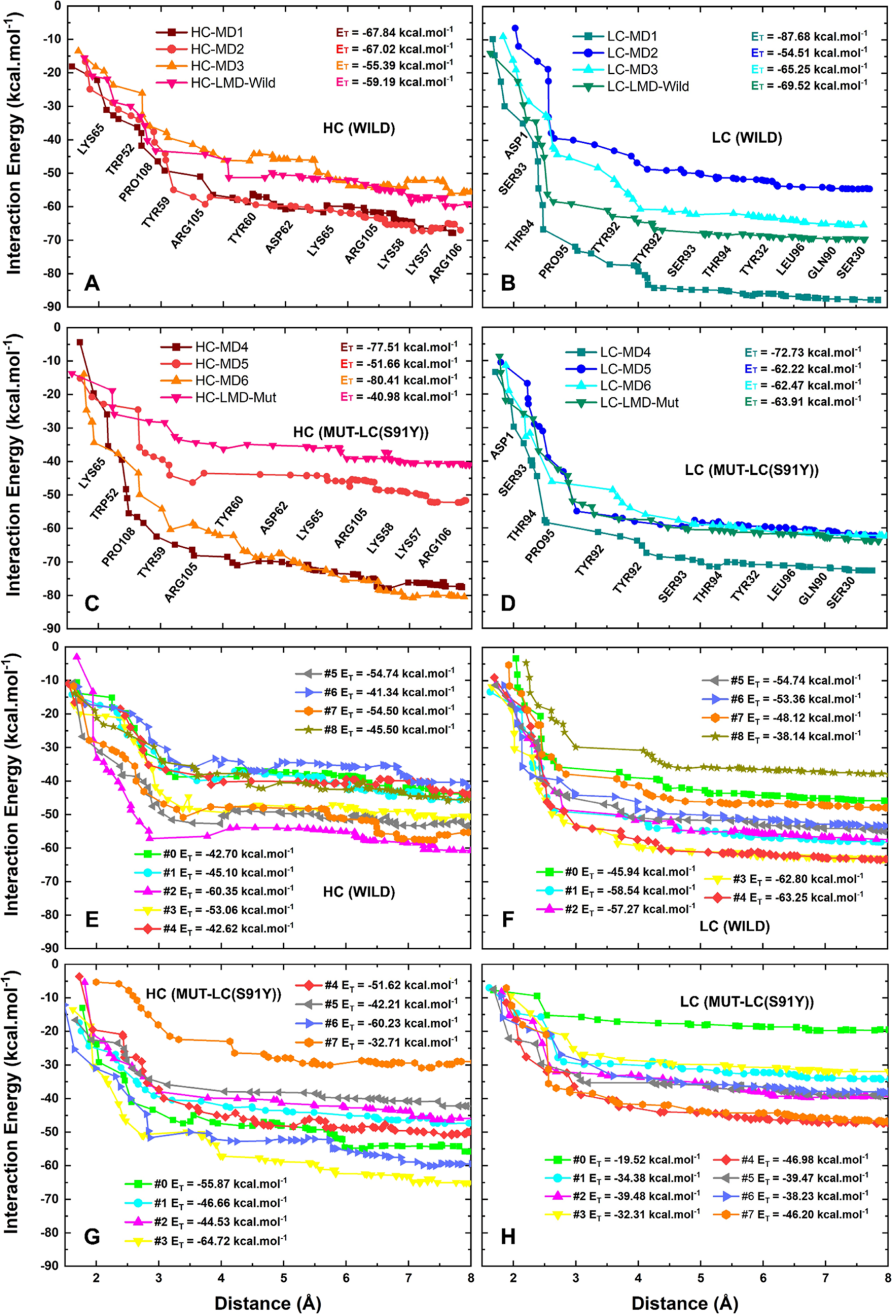
Sum of individual interaction energies involving the Aducanumab-residue::Aβ_2–7_-residue contacts up to 8 Å. The profiles of interaction energy of (A) Aducanumab-HC(Wild):: Aβ_2–7_, (B) Aducanumab-LC(Wild):: Aβ_2–7_, (C) Aducanumab-HC(Mut)::Aβ_2–7_, and (D) Aducanumab-LC(Mut):: Aβ_2–7_ are based on the ensemble of final conformations from MD. The same description of these subsystems (Aducanumab-HC (Wild)/Aducanumab-LC (Wild)/Aducanumab-HC (Mut)/Aducanumab-LC (Mut)::Aβ_2–7_) considering RC are represented in (E–H), respectively. The residues near lines (A–D) make most of the surface contacts.

The close contacts (4 Å) between HC and Aβ_2–7_ are primarily mediated by _HC_LYS65, _HC_TRP52, _HC_PRO108, _HC_TYR59, and _HC_ARG105, while the LC::Aβ_2–7_ close contacts (4 Å) are mainly mediated by _LC_ASP1 and the LCDR3 residues _LC_SER93, _LC_THR94, _LC_PRO95, and _LC_TYR92 (Figure 7A–D). This suggests that Adu’s heavy chain has more interaction points than its light chain, as discussed previously. Quantum biochemistry calculations also provide more definitive data on the differences between wild and mutated complexes, in terms of the binding affinities between Aducanumab and Aβ_2–7_. All values that follow are averages of the interaction energies. The interaction energies between HC and Aβ_2–7_ were −62.42 and −62.65 kcal·mol^–1^ in wild (MD1-LMD-Wild) and mutated (MD4-LMD-Mut) FC, respectively (Figure 7A,C). The affinity of the RC increased slightly from −48.88 kcal·mol^–1^ in the wild ensemble to −49.87 kcal·mol^–1^ in the mutated ensemble (Figure 7E,G). The affinity of LC::Aβ_2–7_ in wild and mutated FC (RC) were −69.26 (−53.57) and −65.33 (−37.07) kcal·mol^–1^ (Figure 7B,D,F,H), respectively. Additionally, the FC indicates that the total interaction energy (sum of HC and LC affinities) was not greatly affected by LC-S91Y, while the RC showed a decrease in the total affinity between Adu and Aβ_2–7_. It is noteworthy that the LMD simulations indicated a tendency toward a reduction in the binding affinity between HC and Aβ_2–7_ on the mutated complex (Figure 7A–D), which corroborates the findings of crystallographic reports that identified L3 as the most crucial LCDR in maintaining the capture of Aβ_2–7_, because its interaction was not significantly affected.^42^ In comparison to the short MD, the long MD demonstrated a significant impact on the affinity of the mutated complex, emphasizing the necessity of long simulations for the validation of mutations in biological systems.

The total interaction energies between Adu and Aβ_2–7_ from their multiple conformations showed significant differences. The convergence of total interaction energies was more successfully achieved for LC::Aβ_2–7_ than for HC::Aβ_2–7_ which can be seen by some slight repulsive interactions after 6 Å (Figure 7). Within wild-FC, the difference between MD1 and MD3 interaction energies, the conformations of highest and lowest affinities, was 34.5 kcal·mol^–1^ which represents ∼28.5% of MD3 affinity (Figure 7A,B). The highest difference found in wild conformations from RC ensemble was −34.00 kcal·mol^–1^, which represents an increase in ∼40% of the #8 conformation’ affinity (Figure 7E,F). The respective differences found in mutated ensembles from FC (MD4-LMD-Mut) and RC (#0–#7) were: −45.31 kcal·mol^–1^ (Figure 7C,D) and −23.21 kcal·mol^–1^ (Figure 7G,H). These differences represent approximately 43.2% and 30.8% of the weaker binding affinity calculated. Moreover, compared to the mutated ensembles, the distributions of total interaction energy values of wild ensembles are more uniform (Figure S10). Additional details concerning the quantum biochemistry calculations performed for each _Adu_Residue::_Aβ_Residue studied here are described in Tables S4–S28.

The LC was identified as the most attractive chain for the epitope based on the interaction energies obtained through quantum mechanical principles. This finding corroborates the earlier experimental reports published by Arndt et al., which demonstrated that this chain was responsible for four of the seven hydrogen bonds between Aducanumab and Aβ_2–7_ detected in the crystallographic solved structure. In contrast, the HC atom established three hydrogen bonds. These outcomes highlight the reliability of quantum biochemistry in the description of biological structural systems.

The role of water molecules in the binding affinity between proteins and distinct molecules^63,64^ is of critical importance. However, the high cost of computational resources associated with quantum biochemistry has hindered the implementation of explicit water on DFT calculations directed to RC, due to the large number of conformations involved (Figure 7). A comparison of distinct DFT calculations (explicit water/COSMO model) directed only to FC MD1, MD2, MD3, MD4, MD5, and MD6 was also performed to assess the impact of explicit water on the measurement of the interaction energy. The comparison revealed that all interaction energies were lower when explicit water was incorporated into the DFT calculations, indicating that the actual binding affinities of RC are undoubtedly more attractive than those calculated using the COSMO solvation model (Figure 7). This finding is consistent with previous reports emphasizing the crucial role of water networks in protein interactions.^63,64^ Please refer to Figure S11 for further details.

### Aducanumab::Aβ_2–7_ Energetic Hot Spots and Multiple Conformations

**Figure 8:**
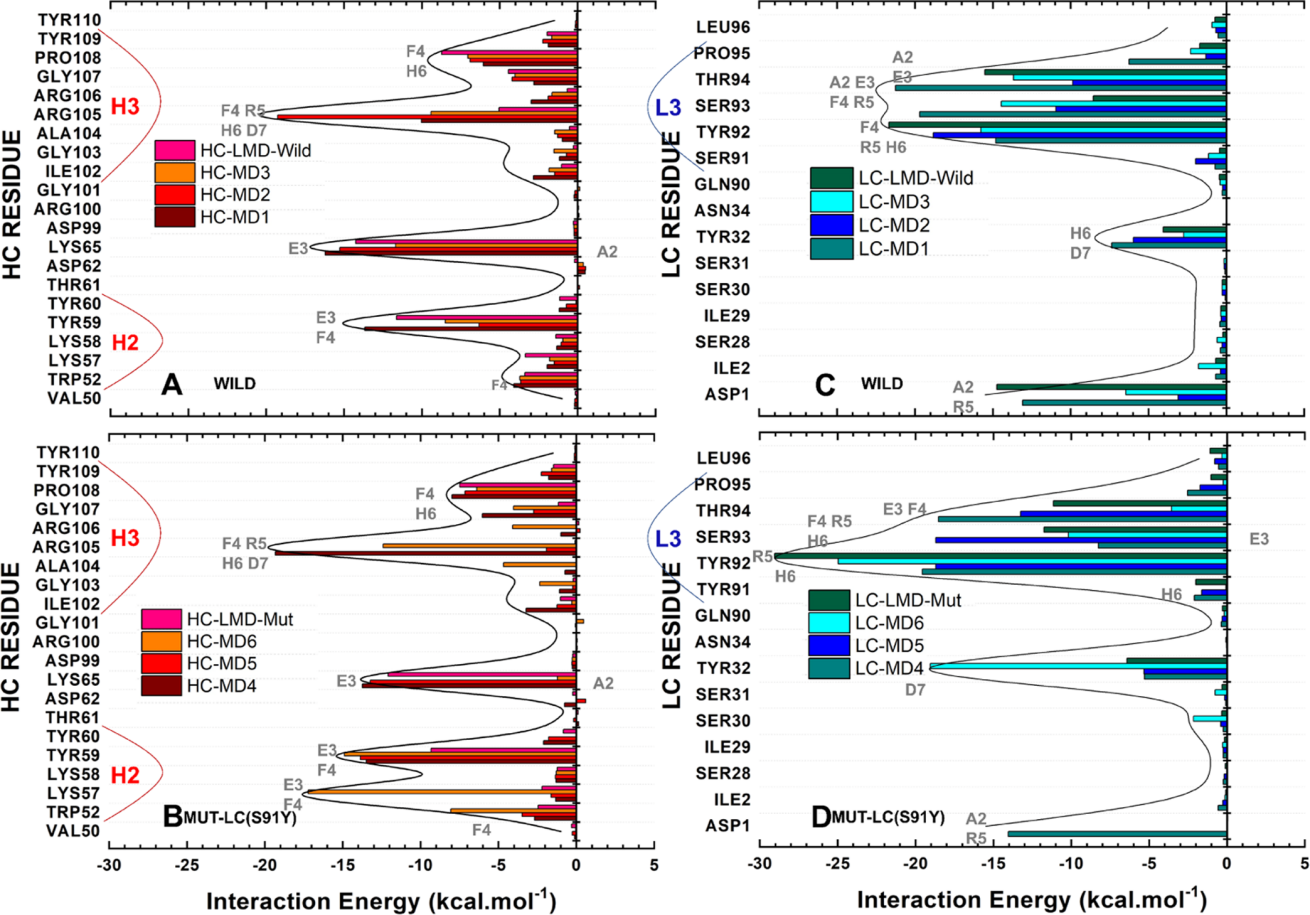
Quantum description of energetic hot spots based on final conformations. The energetic profiles of (A, B) HC and (C, D) LC from wild complex and mutated complexes are located at the top and bottom panels, respectively. The residues (of Aβ_2–7_) colored gray next to the bars are the most critical for each contact.

The quantum biochemistry identified three energetic hot spots, H2, H3, and L3, corresponding to complementarity determinant regions HCDR2, HCDR3, and LCDR3, which account for approximately 77% of the total affinity between Adu and Aβ_2–7_ (Figure 8). This proportion may exceed 80% depending on the structural conformation. _HC_LYS65, _HC_ARG105, _HC_TYR59, and _HC_PRO108 were Aducanumab’s residues predominantly responsible by the HC affinity for Aβ_2–7_ in FC (RC) with interaction energies of −14.36 (−10.43), −10.95 (−10.82), −10.04 (−7.51), and −7.19 (−6.32) kcal·mol^–1^ (Figure 8A). Moreover, residues _LC_TYR92, _LC_THR94, _LC_SER93 and _LC_ASP1 are the most critical to stabilize the Aβ_2–7_ trapped next to LC in FC (RC) presenting interaction energies of −17.77 (−16.08), −15.08 (−13.18), −13.42 (−9.86), and −9.35 (−4.51) kcal·mol^–1^ (Figure 8C). For detailed information regarding the quantum biochemistry description of representative conformations, see Table S29.

The observed differences in affinity between wild and mutated complexes can be attributed directly to the three hot spot segments, H2, H3, and L3. The Aducanumab in the wild-FC exhibits an average interaction energy of −17.92 (−29.56) kcal·mol^–1^ between H2 (H3) and Aβ_2–7_ (Figure 8A). This value decreased (increased) to −25.15 (−24.69) kcal·mol^–1^ (Figure 8A,B). The residues mainly responsible for this difference are _HC_TRP52, _HC_LYS57, and _HC_ARG105 (Figure 8A,B). The L3 experienced a slight increase in its interaction energy from −50.26 to −49.78 kcal·mol^–1^ (Figure 8C,D). As predicted in Table 1, the local binding affinity at position 91 improved, with _LC_SER91 and _LC_TYR91 having −1.08 and −1.45 kcal·mol^–1^, respectively (Figure 8C,D). Additionally, _LC_TYR92 exhibited an enhanced affinity for Aβ_2–7_, particularly at the conformation extracted from long molecular dynamics (Figure 8C,D). However, compared to the wild complex, nearby residues _LC_GLN90, _LC_SER93, _LC_THR94, _LC_PRO95, and _LC_LEU96 showed decreased affinity for Aβ_2–7_ in the mutated complex (Figure 8C,D).

One conceivable rationale for this diminished affinity stemming from the substitution of serine with tyrosine is the divergence in the physicochemical attributes of these amino acids. Serine is characterized by a shorter and predominantly hydrophilic side chain (slightly more polar than tyrosine), whereas tyrosine possesses a longer side chain comprising a phenol, which predisposes it to engage in hydrophobic and hydrophilic interactions. Given that the affinity of Aβ_2–7_ for TYR91 was marginally higher than that for SER91, one potential explanation is that the LC-S91Y mutation may have facilitated novel intramolecular interactions with proximate residues, thereby creating steric hindrance for the Aβ_2–7_::LC interaction. This could account for the diminished affinity of the L3 region for Aβ_2–7_.

**Figure 9:**
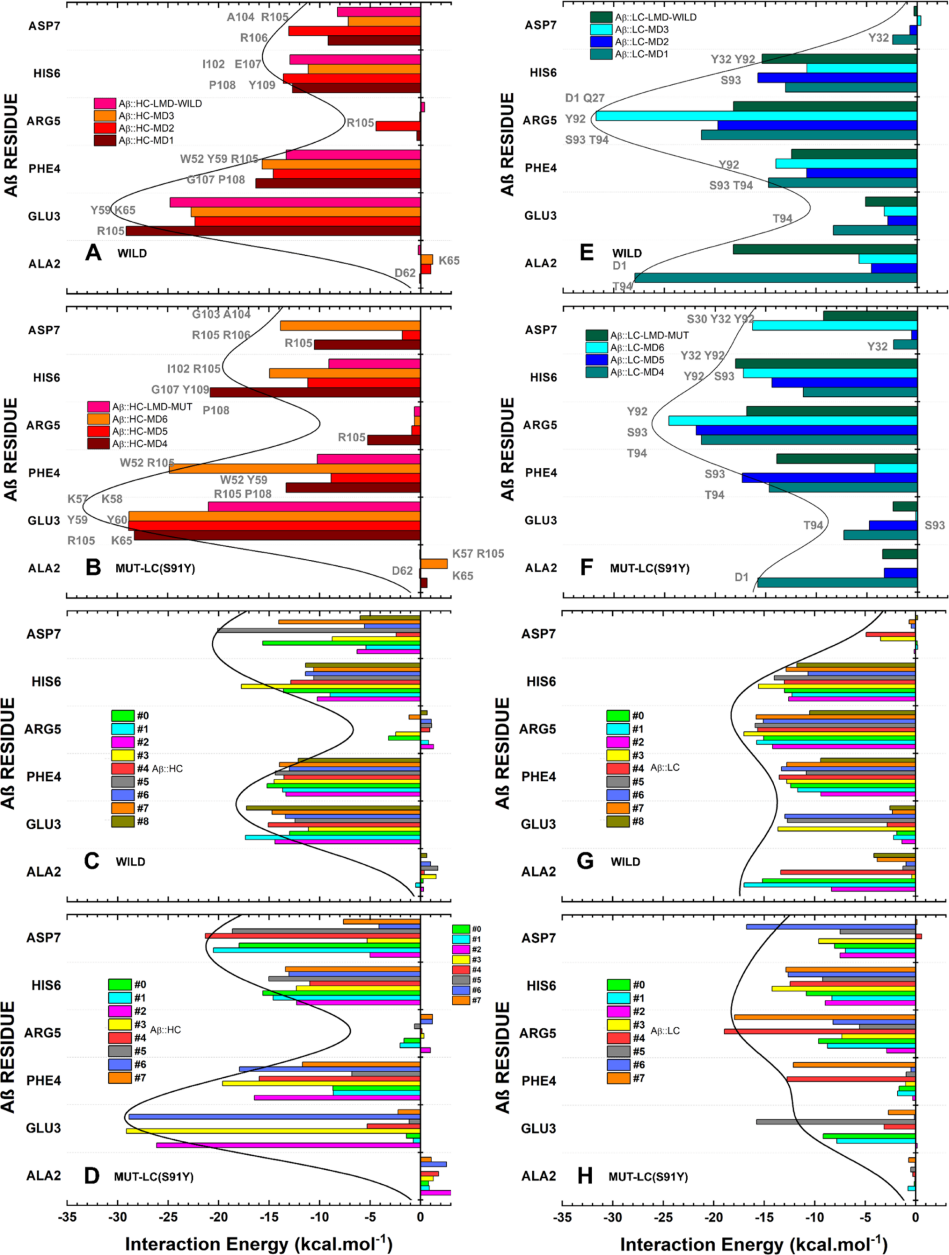
Affinity of Aβ_2–7_ amino acid residues for (A–D) HC Aducanumab in (A, B) final conformations and (C, D) representative conformations, and for (E–H) LC in (E, F) FC and (G, H) RC. The depicted figure illustrates the interaction energy of Aβ_2–7_ residues for (A, C) Wild-HC, (E, G) Wild-LC, (B, D) Mut-HC, and (F, H) Mut-LC. The residues (of Aducanumab) in gray next to the bars are the most critical for each contact.

The _Aβ_GLU3-_Aβ_PHE4 and _Aβ_HIS6-_Aβ_ASP7 are the hot spots of highest affinities for HC in both wild and mutated complexes (Figure 9). In the wild complex, H3 residues are responsible for the main contacts with _Aβ_HIS6-_Aβ_ASP7, while both H3 and H2 are crucial for the contacts with _Aβ_GLU3-_Aβ_PHE4. _HC_LYS65 also makes a critical contribution to the affinity of HC for _Aβ_GLU3. Moreover, the _Aβ_PHE4-_Aβ_HIS6 segment is mainly responsible for the Aβ_2–7_ affinity for wild-LC, while _Aβ_ARG5-_Aβ_ASP7 is the most critical segment for stabilizing the interaction with mutated LC, as shown in Figure 9H. The L3 residues _LC_TYR92-_LC_THR94, with the exception of _LC_ASP1::_Aβ_ARG5, _LC_GLN27::_Aβ_ARG5, and _LC_TYR32::_Aβ_HIS6, are primarily responsible for the energetic contacts between _Aβ_PHE4-_Aβ_ARG5 and wild-LC (Figure 9E). In the mutated complex, _LC_ASP1::_Aβ_ARG5 and _LC_GLN27::_Aβ_ARG5 had minor critical energetic contacts, while new contacts between _LC_SER30/_LC_TYR32/_LC_TYR92 and _Aβ_ASP7 were energetically important (Figure 9F). According to the RC outlined by quantum biochemistry, the mutated complex showed a decreased affinity for HC in _Aβ_ALA2 and _Aβ_GLU3, while _Aβ_HIS6 and _Aβ_ASP7 presented increased affinity (Figure 9). In comparison to the wild complex, all residues except for _Aβ_ASP7, showed a decrease in affinity for mutated LC (Figure 9G,H). The mutation LC-S91Y had a deeper impact on _Aβ_GLU3 and _Aβ_PHE4, as evidenced by the fluctuations in the values of interaction energies within the mutated ensemble (Figure 9B,D,F,H).

The calculated interaction energies suggest that the LC chain is the most crucial in stabilizing the binding of Aducanumab to the Aβ_2–7_ surface and the crystallographic study supports this finding,^42^ as above-discussed. The Adu residues responsible for this binding affinity, in decreasing order of affinity, are as follows: _LC_TYR92, _LC_THR94, _LC_SER93, _HC_LYS65, _HC_ARG105, _HC_TYR59, _HC_PRO108, and _LC_ASP1. In the mutated complex, the heavy chain plays the main role in binding affinity, causing a change in the order of the most attractive residues to _LC_TYR92 > _HC_R105 > _HC_TYR59 > _LC_TYR32 > _LC_SER93 > _HC_LYS65 > _LC_THR94 > _HC_PRO108. Except for _LC_ASP1, _LC_TYR32, and _HC_LYS65, the aforementioned critical residues are part of the groups H2 (_HC_TYR59), H3 (_HC_R105 and _HC_PRO108), and L3 (_LC_TYR92, _LC_SER93, and _LC_THR94). This result is in accordance with previous structural analysis based on X-ray data, which identified these three complementarity determining regions as essential for confining Aβ_2–7_ on surface contact.^42^ It is noteworthy that significant fluctuations in affinities account for up to 40% of the total interaction energy, even when all RMSD values between the Adu::Aβ_2–7_ conformations analyzed here are lower than 1.20 Å. This emphasizes the importance of outlining the flexibility of this complex using multiple binding conformations.

The quantum mechanics calculations performed here reveal that _Aβ_GLU3-_Aβ_PHE4 and _Aβ_HIS6-_Aβ_ASP7 are the main contacts with HC, while the binding affinity for LC is mainly mediated by _Aβ_PHE4, _Aβ_ARG5, and _Aβ_HIS6. These residues are also responsible for the majority of the conserved hydrophobic, salt bridge, and hydrogen bond interactions through multiple Adu::Aβ_2–7_ binding conformations. The computational results are in agreement with previous reports that identified _Aβ_PHE4 and _Aβ_HIS6 as the epitope core^42^ and the most energetic residues,^47^ respectively, followed by _Aβ_ARG5, _Aβ_GLU3, _Aβ_ASP7, and _Aβ_ALA2.^47^ The mutation LC-S91Y resulted in structural instability of Aβ_2–7_, leading to decreased affinities of _Aβ_ALA2/_Aβ_GLU3::HC and _Aβ_ALA2/_Aβ_GLU3/_Aβ_PHE4/_Aβ_ARG5/_Aβ_HIS6::LC, and an increased _Aβ_HIS6/_Aβ_ASP7::HC affinity. Although this mutation did not result in an increased affinity, as predicted in the Screening of Mutations section, our computational outcomes suggest a new binding mechanism that can effectively clear Aβ. One conceivable explanation for this diminished affinity is the discrepancy in the physical and chemical characteristics of SER and TYR. The longer side chain of TYR enables greater hydrophobic and polar interactions, which may explain the higher local affinity between Aβ_2–7_ and _LC_TYR91 in comparison to _LC_SER91 and Aβ_2–7_. However, the new interactions mediated by the long _LC_TYR91 side chain may also create steric hindrances, which could contribute to the observed decrease in the global affinity of the mutant LC by Aβ_2–7_.

Compared to the results of Arndt et al., the quantum mechanical analysis revealed a new critical contact mediated by _HC_LYS65 with an interaction energy of −10.45 kcal·mol^–1^ based on Wild-RC. The binding affinity measurements showed the following affinity order for Aβ_2–7_: L3 > H3 > H2 > L1. This is also in agreement with the crystallographic study, which identified LCDR3 as the primary point of Aβ confinement and L1 as a minor contributor.^42^ The L1 is here represented by _LC_TYR32 with an interaction energy of −4.94 kcal·mol^–1^, based on DFT calculations directed toward the Wild-RC.

### Design of Mimetic Peptides

**Figure 10:**
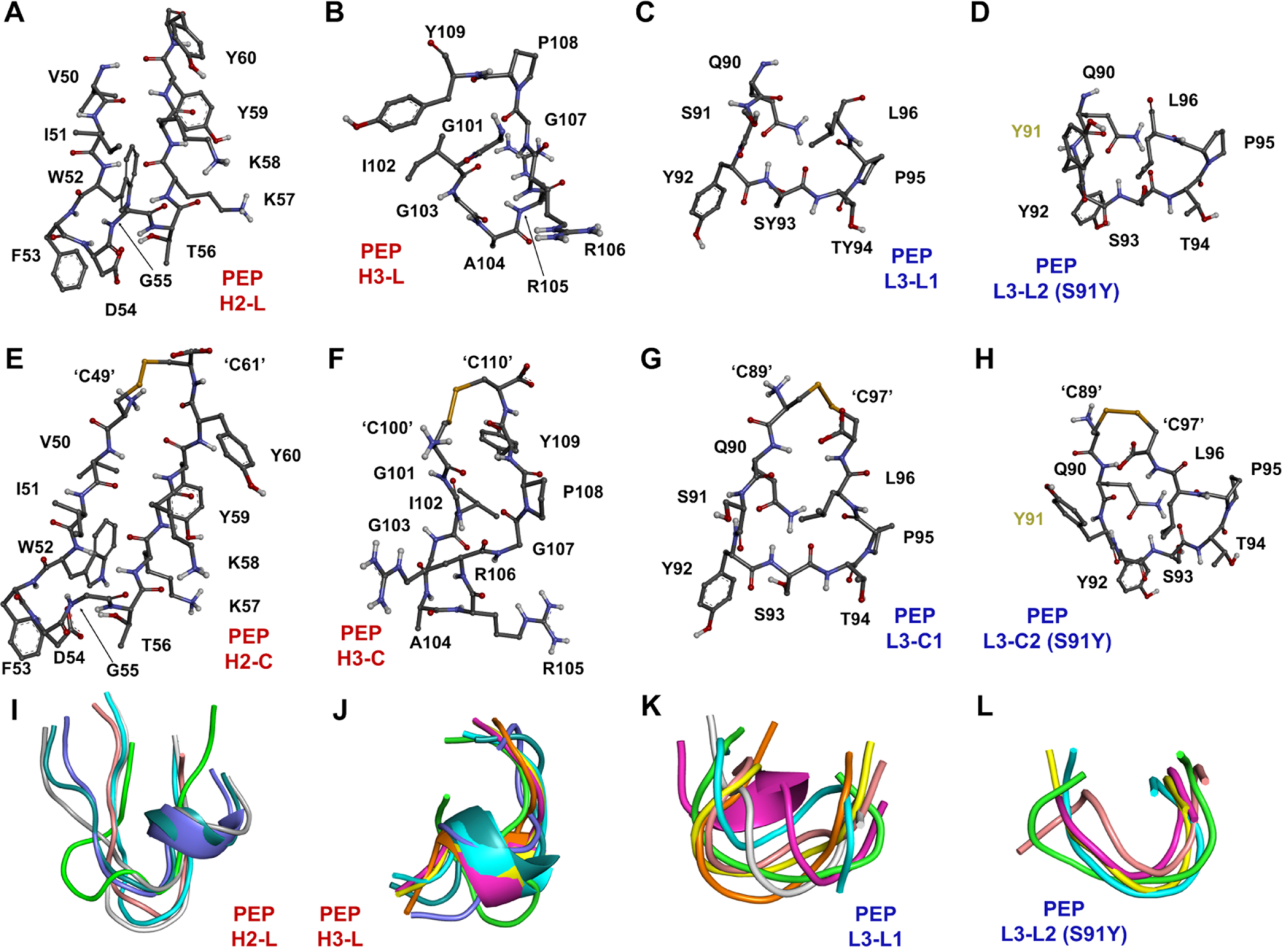
Three-dimensional representation of mimetic peptides. The (A–D) linear and (E–H) cyclic peptides are shown. Cysteine residues have been added to the C- and N-terminus to make the peptides cyclic and are indicated by ‘’. Dark lime green residues refer to the mutation added to LCDR3. (I–L) The alignments of multiple predicted structures for each linear peptide obtained by PEP-FOLD3 are also shown, where only the green peptide represents the structure directly removed from Aducanumab. Peptides with red and blue names are derived from HC and LC, respectively.

The design of mimetic peptides was based on the insights provided by quantum biochemistry calculations, which revealed that three complementarity determining regions are responsible for ∼77% of the affinity of Adu for its epitope. A linear and a cyclic peptide were designed (Figure 10) to mimic the binding capacity of each of the energetic hot spots detected on the chains of aducanumab using a similar approach previously established by Amaral et al.^52^ The proposed molecules are also based on a substantial body of literature on antibody-like mimetic peptides with proven biological activities and advantageous characteristics.^65^ Given that all the peptides proposed here are less than 2 kDa, their production costs are considerably lower than those associated with a full monoclonal antibody, which is approximately 150 kDa.^66^ Moreover, previous research has demonstrated that peptides elicit fewer immunogenic effects and are less toxic than monoclonal antibodies due to their reduced size.^66,67^

Despite the slightly reduced affinity of the mutant LC for Aβ_2–7_, the perturbations resulting from this mutation led us to design two additional mimetic peptides, a linear (Figure 10D) and a cyclic (Figure 10H), with a potentially different mechanism.

The linear mimetic peptides based on the hot spots H2, H3, L3, and mutant L3 were named PEP-H2-L, PEP-H3-L, PEP-L3-L1, and PEP-L3-L2, respectively (Figure 10). These peptides correspond to VAL50 to TYR60, GLY101 to TYR109, GLN90 to LEU96 with SER91, and GLN90 to LEU96 with TYR91. The cyclic peptides are composed of the same amino acid residue with the addition of one N- and one C-terminal cysteine, forming a disulfide bond (Figure 10). Except for PEP-H3-L, the predicted structures of linear peptides indicate that these molecules tend to maintain their secondary structures in a conformation very similar to that of their corresponding CDRs on Aducanumab (Figure 10I–L). The amino acid sequences of these peptides and predictions of their physical and chemical properties are indicated in Table 2. The solubility predictions indicate that linear peptides are more soluble than their derivative cyclic peptides, which may be a feature to consider in drug development (Table 2 and Figure S12).

**Table 2:** Physical and Chemical Properties of Mimetic Peptides

peptide	sequence	pI^a^	molecular weight^a^	Tm index^b^	intrinsic solubility score^c^	net charge^d^
Pep-H2-L	VIWFDGTKKYY	8.40	1419.64	–5.15	0.42	+1
Pep-H2-C	CVIWFDGTKKYYC	8.02	1625.92	–4.15	–0.18	+1
Pep-H3-L	GIGARRGPY	10.84	946.08	1.15	2.10	+2
Pep-H3-C	CGIGARRGPYC	8.96	1152.35	2.62	1.70	+2
Pep-L3-L1	QSYSTPL	5.52	794.86	–6.23	1.75	0
Pep-L3-C1	CQSYSTPLC	5.51	1001.14	–5.72	1.06	0
Pep-L3-L2	QYYSTPL	5.52	870.96	–6.00	1.09	0
Pep-L3-C2	CQYYSTPLC	5.51	1077.23	–5.53	0.25	0

a Isoelectric point and molecular mass calculations of peptide sequences using ProtParam (web.expasy.org/protparam/).

b Tm index calculated using Tm Predictor (tm.life.nthu.edu.tw/)Tm index >1 indicates Tm above 65 °C; Tm index <0 indicates Tm less than 55 °C; 0 < Tm index <1 represents a Tm value between 55 and 65 °C.

c Calculated solubility score on the Chemistry of Health server (www-cohsoftware.ch.cam.ac.uk/index.php/camsolintrinsic) where score >1 indicates good solubility while score <−1 indicates low levels of solubility.

d Net charge of peptides was calculated using the Antimicrobial Peptide Calculator and Predictor (aps.unmc.edu/prediction).

The computational predictions of these mimetic peptides showed that those derived from aducanumab’s HC are potentially more stable than the LC derivatives in an intestinal-like environment (Table 3). Predictions of allergenicity, hemolytic, and toxicity potentials showed that all designed peptides are potentially safe in these regards (Table 3). Since Aβ aggregates, the potential target of these molecules, accumulate in the brain of AD patients,^14^ the potential ability to cross the blood–brain barrier (BBB) was also evaluated, with positive predictions for all peptides (Table 3). Although the peptides have a lower number of cleavage sites susceptible to proteolysis by digestive enzymes than aducanumab, most of them have at least one cleavage site susceptible to the action of chymotrypsin, pepsin, and trypsin (Table 4). The exceptions are PEP-H3-L and PEP-H3-C, which are uniquely resistant to the action of pepsin (Table 4).

**Table 3:** *In Silico* Characterization of Features with Biological Importance

peptide	half-life^a^	stability^a^	antigenic determinants^b^	hemolytic potential^c^	toxin prediction^d^	BBB prediction (score)^e^
Pep-H2-L	1.91	high	0	nonhemolytic	non-toxic	BBB+ (0.15)
Pep-H2-C	1.75	high	0	nonhemolytic	non-toxic	BBB+ (0.17)
Pep-H3-L	1.08	high	0	nonhemolytic	non-toxic	BBB+ (0.25)
Pep-H3-C	1.18	high	0	nonhemolytic	non-toxic	BBB+ (0.25)
Pep-L3-L1	0.90	normal	0	nonhemolytic	non-toxic	BBB+ (0.13)
Pep-L3-C1	0.88	normal	0	nonhemolytic	non-toxic	BBB+ (0.16)
Pep-L3-L2	0.90	normal	0	nonhemolytic	non-toxic	BBB+ (0.12)
Pep-L3-C2	0.88	normal	0	nonhemolytic	non-toxic	BBB+ (0.23)

a Prediction of the half-life/stability of the mimetic peptides in the intestine like environment using HLP (https://webs.iiitd.edu.in/raghava/hlp/).

b The number of antigenic determinants in each peptide sequence was obtained from Predicting Antigenic Peptides (http://imed.med.ucm.es/Tools/antigenic.pl).

c The hemolytic potential of the sequences predicted using HemoPred (http://codes.bio/hemopred/).

d The toxicity potential of the peptides was obtained using ToxinPred (http://crdd.osdd.net/raghava/toxinpred/).

e The classification of the peptides regarding their potential ability to cross the blood–brain barrier was carried out using the parameters of B3Pred (https://webs.iiitd.edu.in/raghava/b3pred/predict.php).

**Table 4:** Susceptibility of Mimetic Peptides to the Action of Digestive Proteases

peptide	chymotrypsin (high specificity)^a^	chymotrypsin (low specificity)^a^	pepsin (pH 1.3)^a^	pepsin (pH > 2)^a^	trypsin^a^
HC-Adu	20	40	34	54	23
LC-Adu	18	37	36	52	18
Pep-H2-L	4	4	2	4	2
Pep-H2-C	4	4	2	4	2
Pep-H3-L	1	1	0	0	2
Pep-H3-C	1	1	0	0	2
Pep-L3-L1	1	2	1	3	0
Pep-L3-C1	1	2	1	3	0
Pep-L3-L2	2	3	1	4	0
Pep-L3-C2	2	3	1	4	0

a Number of sites that can be cleaved by each enzyme in each amino acid sequence. These predictions were carried out by ExPASy PeptideCutter (https://web.expasy.org/peptide_cutter/).

Compared to other anti-Aβ mAbs, Aducanumab inhibits the multiplication of aggregates by secondary nucleation of Aβ monomers at the surfaces of existing Aβ fibrils and promotes the removal of oligomers/fibrils via the immune system.^31^ Its mechanism of action is distinct. However, the use of Aducanumab presents several challenges, including amyloid-related imaging abnormalities (ARIA) caused by edema or effusion, high associated costs, and a limited number of eligible patients.^34−37^ Since ARIA are employed as an inclusion and exclusion criterion to approve continuous treatment based on monoclonal antibodies,^34^ it is crucial for the development of new Alzheimer’s disease drugs that have fewer adverse effects and a more favorable benefit-risk profile. As the field of peptide research expands, multiple potential activities are being proposed, including antimicrobial,^68,69^ anti-inflammatory,^70,71^ antidiabetic,^72,73^ and anti-Alzheimer.^74,75^ The potential anti-Alzheimer peptides that have been previously proposed are typically based on rational design, as exemplified by the bicyclic peptide developed by Ikenoue et al. or extracted from biological sources, such as kefir, by Malta et al. In addition to exhibiting antioxidant and antiacetylcholinesterase properties,^75^ these peptides demonstrated the capacity to modulate Aβ aggregation (*in vitro*) and suppress Aβ42 toxicity in *Caenorhabditis elegans* (*in vivo*).^74^

Based on these data and the unique mechanism of action of Aducanumab, it is expected that mimetic peptides based on the interaction energy hot spots H2, H3, and L3, which account for approximately 77% of the Adu affinity of Aβ, have the potential to inhibit this secondary nucleation with reduced cost and low side effects.^65,66^ In addition to the three mimetic peptides directly extracted from Aducanumab’s chains, a peptide based on mutated L3 was also designed. Four corresponding cyclic peptides were also designed. In addition to the interaction energy, another factor suggesting the potential usability of these peptides is the peptide folding predictions. These predictions revealed that the linear peptides PEP-H2-L, PEP-L3-L1, and PEP-L3-L2 tend to retain their three-dimensional structure, such as that obtained directly from Aducanumab. This is critical because conformational changes have the potential to disrupt the peptide::Aβ interactions.^76,77^*In silico* predictions also indicate that all peptide sequences are nonallergenic, nonhemolytic, nontoxic, and BBB+ (positive for crossing the blood–brain barrier). Although all sequences were indicated as potentially susceptible to intestinal proteases, cyclic peptides may be less susceptible to proteases,^78^ making oral administration a potential option. Moreover, the cyclic peptides that were designed in this study are distinct from those that were previously theoretically obtained by quantum biochemistry. This is due to the fact that cysteines were added to the N- and C-terminal portions of the linear peptides before a conventional end-to-end cyclization method was employed.^79^

The binding affinity of a drug candidate represents a pivotal parameter in the drug development process, as it has the potential to significantly impact the drug’s efficacy. This is exemplified by the case of certain corticotropin-releasing hormone receptor 1 antagonists.^80^ This property is typically assessed through a combination of computational and experimental methods.^81^ This property is typically evaluated through a combination of computational and experimental methods. In this context, and based on the results of quantum analysis, peptides derived from L3 (PEP-L3-L1 and PEP-L3-C1) have been identified as promising candidates. These peptides have been designed to target the most energetic hot spot and, in theoretical terms, are the most promising for forming a peptide::Aβ complex and impairing Aβ aggregation. The HCDR3-based molecules exhibited the most favorable predictive parameters with respect to protease resistance and the capacity to cross the blood–brain barrier. This potential resistance to proteolysis may facilitate the development of orally administered drugs,^82^ while the ability to cross the BBB is critical to obtaining an anti-Aβ, as its pathological accumulation in Alzheimer’s disease occurs in the brain.^14^ While these predictions require further experimental validation, they indicate that peptides derived from HCDR2 may be the least promising for further drug development.

Synthetic peptides were previously investigated for their druggability and therapeutic use as antimicrobial agents through *in silico* analysis.^83^ These computational results were confirmed by hemolysis and toxicity assays, which validated the reliability of the *in silico* predictions performed here. A variety of synthetic peptides have demonstrated the ability to bind to Aβ, resulting in modulation or inhibition of Aβ aggregation.^74,84−86^ Altogether, the quantum mechanical outcomes, *in silico* predictions, and previous reports of anti-Aβ peptides^74,84−86^ suggest that it is possible to obtain peptides with a mechanism of action similar to that of Aducanumab. While the synthetic peptides proposed here cannot achieve the typical focus of anti-Aβ immunotherapies, which are based on immune signaling and removal of Aβ aggregates through microglia-mediated phagocytosis, it is believed that they affect the perturbation of the secondary nucleation, mechanism previously linked to the Adu efficacy and specificity.^31^ Thus, we are confident that these findings suggest that these molecules should have potential applications in the therapy of Alzheimer’s disease. To confirm and measure the peptides’ ability to bind to Aβ and inhibit Aβ fibrilization/aggregation, thioflavin T fluorescence assays,^86^ and atomic force microscopy^74^ are being conducted. Peptides containing LC-S91Y will also be evaluated, which may elucidate whether this mutation affects the affinity of LCDR3 for Aβ. Further assays should be performed to confirm the mechanism of action and *in vivo* efficacy of the peptides.

## Conclusions

In summary, only the LC-S91Y mutation was selected for further testing by using more robust computational techniques. Computational outcomes revealed that Aβ_2–7_ has a minor binding affinity and is structurally less stable when bound to the mutated version of aducanumab. This could suggest a distinct binding mode or simply a minor propensity of Aβ_2–7_ to remain bound to Adu. Further assays should be conducted to confirm this behavior and to determine whether the affinity really decreases. The results of the classical simulations demonstrated the significance of the LMD in the context of a mutated complex lacking a crystallographic structure. Furthermore, the application of short MD was found to be an effective approach for achieving affinity levels comparable to those obtained through LMD. These findings highlight the potential of short MD simulations as a viable alternative to LMD, offering a more efficient method for studying complex molecular interactions in the study of Adu::Aβ_2–7_. Quantum biochemistry indicated the presence of three distinct segments (L3, H3, and H2) that could serve as templates for the design of synthetic peptides with a mechanism of action similar to that of Aducanumab. Although a reduction in the affinity of the mutated L3 for Aβ_2–7_ was observed, this segment was also employed in the design of mimetic peptides for the purpose of comparing the binding capacity of wild-type and mutated L3 derivatives through further experimental procedures. The new insights provided by the computational results indicate that the eight mimetic peptides based on Aducanumab’s energetic hot spots exhibit promising characteristics, suggesting that they may be a promising line of inquiry in further research. In addition, quantum biochemistry and bioinformatic predictive parameters suggest that peptides Pep-H3-L, Pep-H3-C, Pep-L3-L1, and Pep-L3-C1 are the most promising, making them the most appropriate candidates for future *in vitro* and *in vivo* testing.

## Methods

### Preparing Aducanumab::Aβ_2–7_

The crystallographic structure of Aducanumab::Aβ_2–7_ (ID 6CO3 - resolution 2.38 Å)^42^ was obtained from the Protein Data Bank.^87^ Essential residues that were missing in Aducanumab’s heavy chain (SER139-GLY145) were modeled using SWISSMODEL.^88^ Playmolecule ProteinPrepare^89^ carried out the protonation adjustments of the amino acid side chains at pH 7.4. This prepared complex was used for screening mutations (2.2) and molecular dynamics (2.3). For more details about this modeled structure and main surface stabilizing contacts, see Figure 1.

### Screening of Mutations

The first step in identifying potential mutations that could increase the affinity of Adu for Aβ_2–7_ was to conduct an initial screening of potential mutations in the heavy chain (HC) and light chain (LC) using BeAtMuSiC 1.0.^90^ The most promising mutations, according to BeAtMuSiC energetic parameters, were then scrutinized by DeepDDG^91^ and MutaBind2^92^ to evaluate potential changes in stability and affinity. DeepDDG utilized a nonintegrative model for its assays, while MutaBind2 predictions were based on a single mutation per run. These three predictive parameters were utilized to select mutations with significant potential to enhance the affinity of Aducanumab for Aβ_2–7_ without decreasing the stability.

### Molecular Dynamics (MD)

In line with a previous theoretical work,^47^ the Adu’s HC and LC were reduced to GLN1-SER126 and ASP1-THR109, corresponding to a variable fragment heavy chain (HC) and a variable fragment of light chain (LC), to diminish the computational cost during simulations (Figure 1B).^47^ Moreover, a mutated Adu::Aβ_2–7_ was created using the selected mutation (LC-S91Y), which was inserted in the Adu::Aβ_2–7_. Then, two Adu::Aβ_2–7_ complexes, wild and mutated, were submitted to three short and independent all-atom MD simulations of 100 ns and one long MD of 1 μs using the GROMACS 2023 package.^93^ The same scheme was repeated for a system containing only Aβ_2–7_ to compare the structural stability and folding of its bound and unbound states: three independent and short MDs and a single long MD. The force field CHARMM36 was used to set the interatomic potential during simulations.^94^ Each complex was initially inserted into a water box based on TIP3P CHARMM modified water model^95^ and neutralized with counterions Na^+^ and Cl^–^ (0.15 mol·L^–1^). The steepest descent algorithm was used to minimize the potential energy using the maximum force <10.0 kJ mol^–1^. An equilibration phase of 2 ns was performed using NVT (1 ns) and NPT (1 ns) ensembles to equilibrate the temperature and pressure of the system. Temperature coupling was set at 300 K through V-rescale thermostat^96^ and the Parrinello–Rahman barostat^97^ was configured to stabilize the pressure with a compressibility of 4.5 · 10^–5^. Subsequently, the position restraints were removed, and the molecular dynamics was performed. This step-by-step procedure was performed in triplicate for each complex. The Particle Mesh Ewald (PME) method was used to define long-range electrostatic interactions.^98^ Leapfrog integration was employed to integrate the differential equations of motion,^99^ and the LINCS algorithm was used to reset covalent bonds to their appropriate lengths.^100^ Furthermore, a time step of 2 fs was used. Finally, the *gmx trjconv* algorithm was used to extract the initial and final conformations of each MD assay. Also, *gmx rmsd* and *gmx rmsf* were used to obtain the RMSD and RMSF data.

### Structural Ensemble Generation

Two MD samples were used to generate the conformational ensemble: one from the wild-type and one from the mutant complex. The criterion choice was the level of structural flexibility, where the MD assays with highest deviations and fluctuations have been chosen. Both the initial conformation and the trajectory file of these simulations were used as input to generate the ensemble of representative conformations in EnGens.^62^ Furthermore, initial data of binding affinity was used to select the amino acid residues to be scrutinized in terms of dihedral angles of the backbone and pairwise distances between residues. The dimensionality reduction was performed through Uniform Manifold Approximation and Projection.^101^ The structural clustering was carried out using Gaussian Mixture Models^102^ and the silhouette method^103^ was performed to achieve the optimal number of representative conformations for each MD assay outlined. The conformations occupying the cluster’s hubs were then extracted and outlined through interactions description (2.5) and quantum biochemistry (2.6). The two ensembles generated were named representative conformations (RCs) of the wild (Wild-RC) and mutated (Mut-RC) complexes.

### Nonbonded Interactions Description

Hydrogen bonds and hydrophobic interactions were identified using LigPlot+ version 2.2.^104^ The distances between the hydrogen (H), acceptor atom (A), and donor atom (D) were analyzed to identify hydrogen bonds, which were confirmed when the distances between H–A and D–A were within the ranges of 2.70 and 3.35 Å, respectively. Hydrophobic interactions were detected based on the fulfillment of minimum and maximum distances of 2.90 and 3.90 Å between amino acid residues of the Aducanumab and Aβ_2–7_.

### Quantum Biochemistry

The final conformation of each molecular dynamics simulation and representative conformations obtained in Aducanumab::Aβ_2–7_ Energetic Hot Spots and Multiple Conformations section were submitted to the molecular fractionation with conjugated caps (MFCC), splitting the Adu::Aβ_2–7_ interface contacts into subsystems and decreasing the computational cost of the binding affinity calculations between Adu and Aβ amino acid residues.^49−54,105−108^ These MFCC calculations are based on DFT and allow us to obtain the interaction energies between residues (Ri and Rj) present in the different protein chains (HC-Adu/LC-Adu and Aβ), as shown in the following scheme:
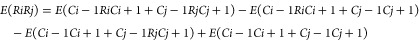
The initial term of the equation, E(Ci – 1RiCi + 1 + Cj – 1RjCj + 1), delineates the cumulative energy (E) associated with the interaction between residues Ri and Rj and their respective molecular caps. The subsequent component, E(Ci – 1RiCi + 1 + Cj – 1Cj + 1), corresponds to the energy of the system formed by Ri with its molecular caps and the caps of Rj. The third term, E(Ci – 1 Ci + 1 + Cj – 1RjCj + 1), indicates the total interaction energy from the ensemble comprising Rj, its molecular caps, and the Ri caps. In the last part, E(Ci – 1 Ci + 1 + Cj – 1Cj + 1) characterizes the interaction energy inherent to the system exclusively encompassing the molecular caps of both Ri and Rj. Herein, Ci – 1, Ci + 1, Cj – 1, and Cj + 1 symbolize the molecular caps, which encompass residues covalently linked to the amino or carboxyl groups of Ri and Rj, and hydrogen atoms filling the gaps caused by molecular fragmentation.

The quantum biochemistry study conducted energy interaction analyses focusing on noncovalent interactions between Adu (Ri) and Aβ_2–7_ (Rj) residues within a maximum distance threshold of 8.0 Å. Additionally, the energetic contributions of explicit water molecules positioned within 2.5 Å of these residues (Ri and Rj) were considered in the quantum mechanical calculations of the final conformations. The ensembles of Wild-RC and Mut-RC were outlined without considering the explicit water in their quantum calculations due to the high number of conformations present. In contrast, the RCs were delineated through the employment of the COSMO solvation model, which was utilized to represent the waters in proximity to surface interactions.^109^ Furthermore, the majority of FC (MD1, MD2, MD3, MD4, MD5, and MD6) were also subjected to DFT calculations employing only the COMO solvation model. This was done to gain insight into the real impact of explicit water in quantum calculations.

Based on a previous study by our research group, it was found that DFT calculations using a dielectric constant of 40 were more suitable for simulating the electrostatic environment around a protein::protein interaction surface than a lower dielectric constant of 4.^51^ Therefore, a dielectric constant of 40 was used for the DFT calculations carried out in this current study. For each distinct set of final atomic coordinates derived from molecular dynamics simulations, the MFCC scheme was initially applied, followed by Density Functional Theory calculations using the DMOL3 package.^110^ The DFT calculations carried out by using generalized gradient approximation (GGA) with functional PBE,^111^ and TS scheme.^112^ A homemade Phyton-based program was used to straightforwardly determine the interactions between the Adu (Ri) and Aβ_2–7_ (Rj) residues of interest through DFT calculations.

### Design of Aducanumab-Based Mimetic Peptides

The linear and cyclic peptides were designed following an adapted protocol established by Amaral et al.^52^ The linear and cyclic peptides were designed by using the energetic hot spots of HC and LC as templates. These critical regions were extracted from the first MD assay of each complex, corresponding to the MD1 (wild) and MD4 (mutated) final conformations. The linear peptides correspond precisely to the segments isolated from Adu. Cysteine residues were added to their N- and C-terminus, and a disulfide bond was subsequently formed between them to obtain the cyclic peptides. Thus, each linear peptide has a corresponding cyclic peptide. The cyclic peptides were optimized, and their sterically acceptable structures were predicted using the Dreiding-like force field.^113^ This force field employs elements, bond orders, number of bonds, and valence to calculate energy in a fast and efficient manner.

### *In Silico* Characterization of Mimetic Peptides

Various computational tools were used to perform sequence-based predictions regarding critical pharmacokinetic characteristics in drug discovery. These predictions are becoming increasingly important for saving time and costs in drug development research.^114,115^ PEP-FOLD3^116^ was used to predict potential tridimensional conformations of the linear mimetic peptides by using sOPEP to sort cluster models. The ExPASy ProtParam was used to predict the isoelectric point and molecular weight.^117^ The Tm Predictor (http://tm.life.nthu.edu.tw/) was used to predict the melting temperature of each peptide. The CamSol method^118^ and APD3^119^ were used to calculate the solubility and net charge of the peptides, respectively. The stability of each peptide in an intestinal-like environment was evaluated using the HLP web server.^120^ Additionally, susceptibility to chymotrypsin, pepsin, and trypsin was assessed using ExPASy PeptideCutter.^117^ The number of antigenic determinants for the peptide sequences were obtained using Predicting Antigenic Peptides (http://imed.med.ucm.es/Tools/antigenic.html) according to the method of *Kolaskar and Tongaonkar*.^121^ HemoPred^122^ and ToxinPred^123^ were used to evaluate the hemolytic and toxic potentials of these molecules, respectively. Additionally, the potential capacity to cross the blood–brain barrier was investigated using B3Pred.^124^

## Supporting Information Available

Detailed analyses and visualizations supplementing the main text. These include RMSD and RMSF values for molecular dynamics (MD) simulations of wild and mutated Aducanumab::Aβ2–7 complexes (Figures S1–S3), hydrogen bond interactions across multiple MD replicas (Figures S4 and S5), structural illustrations of final conformations (Figures S6 and S7), interaction schematics (Figures S8 and S9), energy distributions and solvation effects (Figures S10 and S11), and peptide solubility predictions (Figure S12). Nonbonded interaction data for various MD simulations are presented in Tables S1–S3. Additionally, details of quantum mechanics calculations are depicted in Tables S4–S29. (PDF)

## Abbreviations

AB: amyloid-β
ACH: amyloid cascade hypothesis
AD: Alzheimer’s disease
APP: amyloid precursor protein
Adu: aducanumab
CDR: complementarity determining region
FC: final conformations
FDA: Food and Drug Administration
HC: heavy chain
HCDR: heavy complementarity determining region
LC: light chain
LCDR: light complementarity determining region
LMD: long molecular dynamics
Lec: lecanemab
MD: molecular dynamics
mAb: monoclonal antibody
RC: representative conformations
RMSD: root-mean-square deviation
RMSF: root-mean-square fluctuations
THH: tau hyperphosphorylation hypothesis
