# Supplementary material for: Alzheimer’s Disease Immunotherapy and Mimetic Peptide Design for Drug Development: Mutation Screening, Molecular Dynamics, and a Quantum Biochemistry Approach Focusing on Aducanumab::Aβ2–7 Binding Affinity
Source: ACS Chem Neurosci. 2024 Sep 20;15(19):3543–62. doi: 10.1021/acschemneuro.4c00453 (PMC11450751; doi:10.1021/acschemneuro.4c00453)
Supplement: Supplementary file 1 — cn4c00453_si_001.pdf [file cn4c00453_si_001.pdf]

Supporting Information

Alzheimer's disease immunotherapy and mimetic peptides design for drug development: mutations screening, molecular dynamics, and a quantum biochemistry approach focusing on Aducanumab::Aβ2-7 binding affinity

Victor L.B. França*, Eveline M. Bezerra, Roner F. da Costa, Hernandes F. Carvalho, Valder N. Freire, and Geanne Matos

Author Information

Victor L.B. França* − *Department of Physiology and Pharmacology, Federal University of Ceará, 60430-270, Fortaleza, Ceará, Brazil*

Eveline M. Bezerra − *Department of Sciences, Mathematics and Statistics, Federal Rural University of Semi-Arid (UFERSA), 59625-900, Mossoró, RN, Brazil*

Roner F. da Costa − *Department of Sciences, Mathematics and Statistics, Federal Rural University of Semi-Arid (UFERSA), 59625-900, Mossoró, RN, Brazil*

Hernandes F. Carvalho − *Department of Structural and Functional Biology, Institute of Biology, State University of Campinas, 13083-864 Campinas, São Paulo, Brazil.*

Valder N. Freire − *Department of Physics, Federal University of Ceará, 60430-270, Fortaleza, Ceará, Brazil*

Geanne Matos − *Department of Physiology and Pharmacology, Federal University of Ceará, 60430-270, Fortaleza, Ceará, Brazil*

*Corresponding Author:

Victor L.B. França

Email address: victor.bernardesf@alu.ufc.br,

ORCID: https://orcid.org/0000-0002-2731-3897

Postal address: Department of Physics, Federal University of Ceará, CE, Brazil; Laboratory of Applied Biophysics, Av. Mister Hull, Caixa Postal 60451 Fortaleza, CE, Brazil. Tel: +55 85 33669822; Fax: +55 85 33669789.

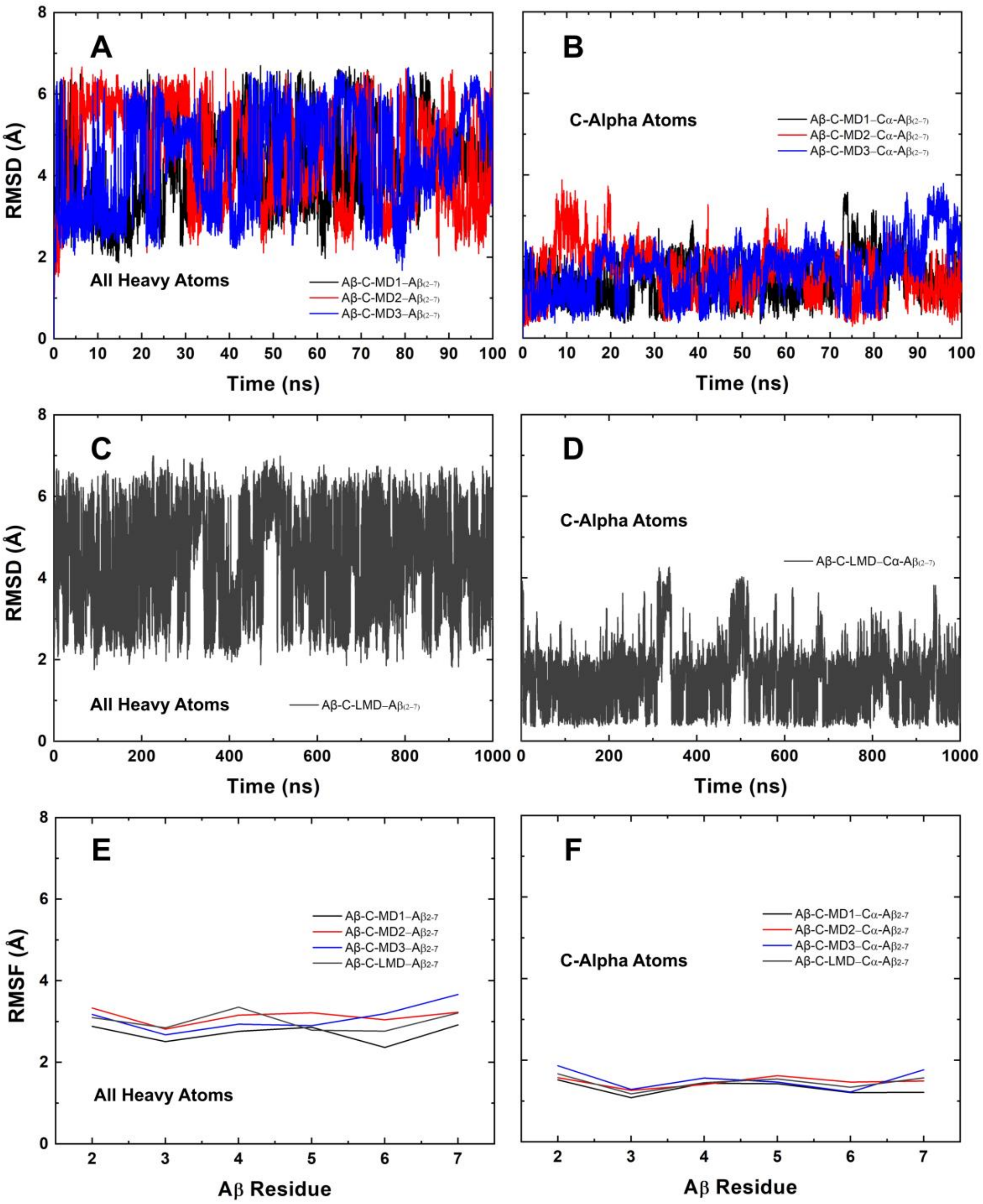

Figure S1 – (A-B) RMSD values over time for short simulation replicas. (C-D) RMSD values over time for long simulations of unbounded $A\beta_{2\text{-}7}$. (E-F) Comparison of RMSF values of residues in the unbounded state of $A\beta_{2\text{-}7}$, considering short MD replicas and long MD simulations.

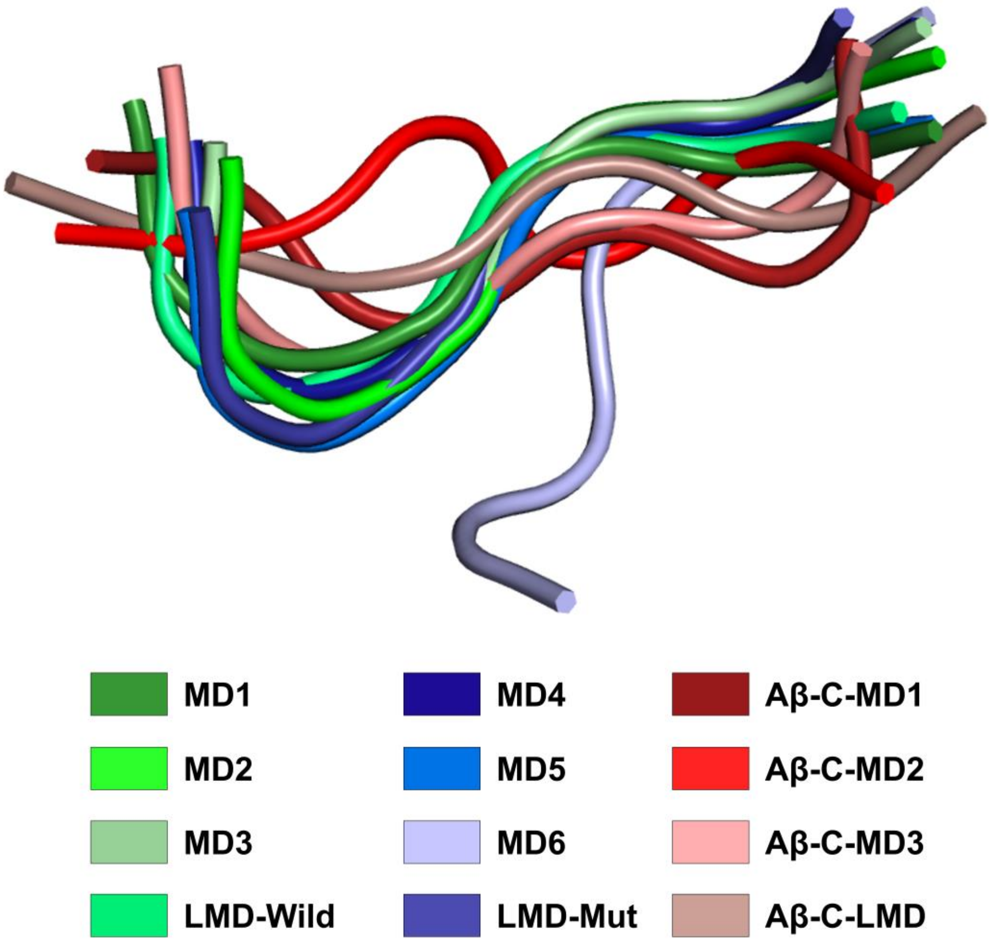

Figure S2 – Structural alignment between A$\beta_{2\text{-}7}$ conformations at the final atomic coordinates, as provided by molecular dynamics simulations of Adu-Wild::A$\beta_{2\text{-}7}$, Adu-Mut::A$\beta_{2\text{-}7}$, and A$\beta_{2\text{-}7}$ alone. The conformations are colored in shades of green, purple, and red, respectively.

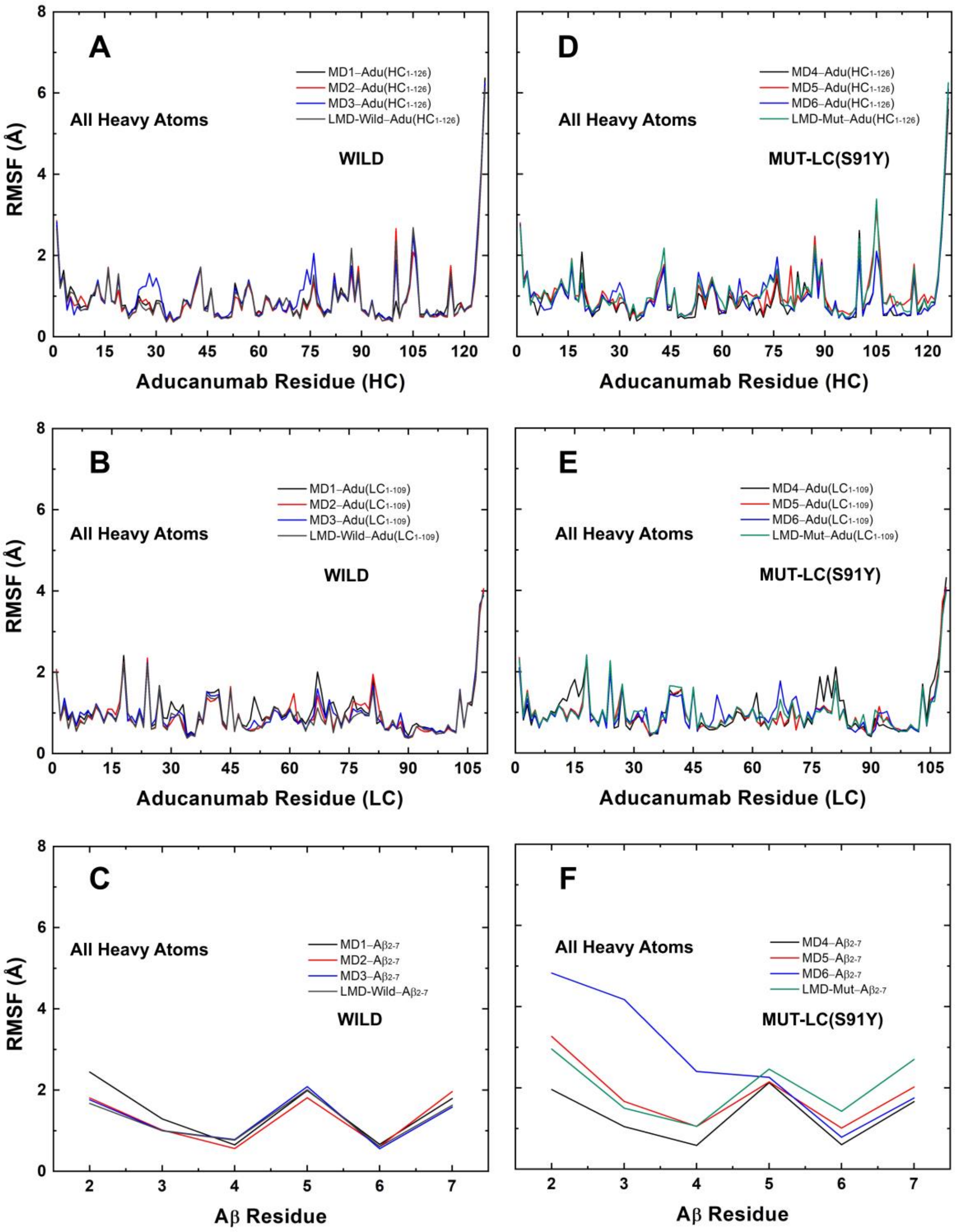

Figure S3 – RMSF of (A-C) wild and (D-F) mutated Aducanumab::Aβ_{2-7} based on all heavy atoms.

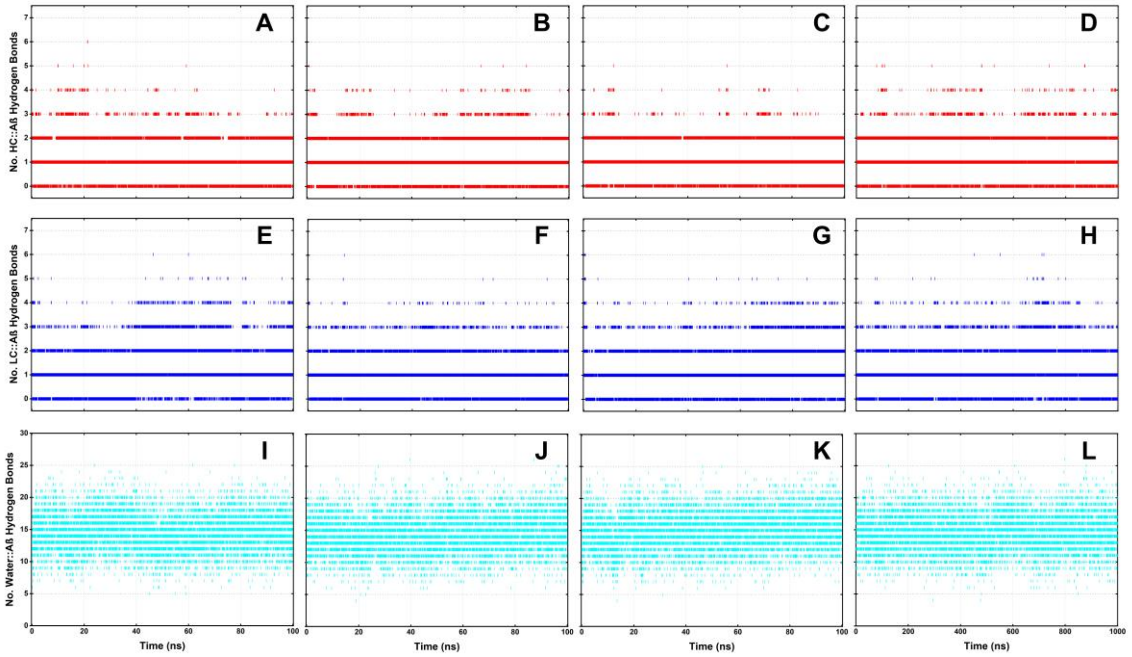

Figure S4 – Number of hydrogen bonds established in (A-D) Adu-HC::A$\beta_{2\text{-}7}$, (E-H) Adu-LC::A$\beta_{2\text{-}7}$, and (I-L) water::A$\beta_{2\text{-}7}$ interfaces. (A, E, and I) MD1, (B, F, and J) MD2, (C, G, and K) MD3, and LMD-Wild (D, H, and L) data are illustrated.

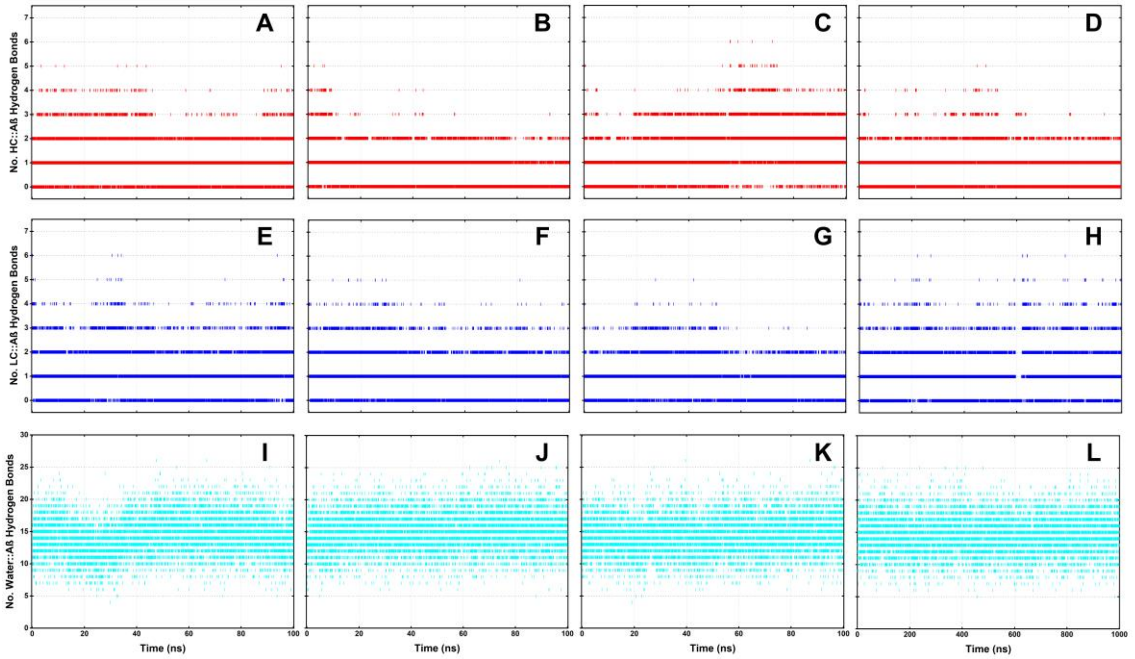

Figure S5 – Number of hydrogen bonds established in (A-D) Adu-HC::A$\beta_{2\text{-}7}$, (E-H) Adu-LC::A$\beta_{2\text{-}7}$, and (I-L) water::A$\beta_{2\text{-}7}$ interfaces. (A, E, and I) MD4, (B, F, and J) MD5, (C, G, and K) MD6, and LMD-Mut (D, H, and L) data are illustrated.

Table S1 – Description of nonbonded interactions calculated on the Aducanumab::Aβ_{2-7} interface comprise hydrophobic, salt bridge, and hydrogen bond contacts. These interactions were determined in the final atomic conformation of MD1, MD2, MD3, MD4, MD5, MD6, LMD-Wild (LMD-W), and LMD-Mut (LMD-M) samples.

Interaction	Adu Residue	Adu Chain	Aβ Residue	Sample (MD)	Distance (Å)
	TRP52	Heavy	PHE4	MD1/MD2/MD3/MD6/LMD-W/	
	LYS57	Heavy	GLU3	MD6	
	LYS57	Heavy	PHE4	MD6	
	TYR59	Heavy	GLU3	MD1/MD2/MD4/MD5/MD6/LMD-W/LMD-M	
	TYR59	Heavy	PHE4	MD4/MD5/MD6/LMD-W	
	LYS65	Heavy	GLU3	MD1/MD2/MD3/MD4/MD5/LMD-W/LMD-M	
	ILE102	Heavy	HIS6	MD1/MD3/MD4/MD6/LMD-W	
	ALA104	Heavy	ASP7	MD6	
	ARG105	Heavy	PHE4	MD2/MD4	
	ARG105	Heavy	ARG5	MD2/MD4	
	ARG105	Heavy	ASP7	MD2/MD4	
	GLY107	Heavy	PHE4	MD1/MD2/MD3/MD4/LMD-W	
	GLY107	Heavy	HIS6	MD1/MD2/MD4/MD5/MD6/LMD-W	
	PRO108	Heavy	PHE4	LMD-W/LMD-M	
	PRO108	Heavy	HIS6	MD1/MD2/MD3/MD4/MD5/MD6/LMD-M	
Hydrophobic	ASP1	Light	ALA2	MD1/MD4/ LMD-W	
	ILE2	Light	ARG5	MD3	
	GLN27	Light	ARG5	MD3	
	TYR32	Light	HIS6	MD1/MD2/MD5/MD6/LMD-W/LMD-M	
	TYR32	Light	ASP7	MD6	
	SER91	Light	HIS6	MD1/MD2/MD3/LMD-W	
	TYR91	Light	HIS6	MD4/MD5/MD6/LMD-M	
	TYR92	Light	ARG5	MD1/MD2/MD3/MD4/MD5/MD6/LMD-W/LMD-M	
	TYR92	Light	HIS6	MD2/MD3/MD4/MD5/MD6/LMD-W/LMD-M	
	TYR92	Light	ASP7	LMD-M	
	SER93	Light	ALA2	MD1/MD3/MD4/MD5/LMD-W/LMD-M	
	SER93	Light	PHE4	MD1/MD2/MD3/MD4/MD5/LMD-W/LMD-M	
	SER93	Light	ARG5	MD1/MD2/MD3/MD6/LMD-W	
	THR94	Light	ALA2	MD1/MD3/MD4/MD5/LMD-W/LMD-M	
	THR94	Light	GLU3	MD3/MD4/MD5/LMD-W	
	THR94	Light	PHE4	MD1/MD2/MD4/MD5/LMD-M	
	PRO95	Light	ALA2	MD1	
	LYS65(NZ)	Heavy	GLU3(OE1)	MD4	
Salt Bridge	LYS65(NZ)	Heavy	GLU3(OE2)	MD3	
	ARG105(NH1)	Heavy	ASP7(OD2)	MD2	
	ASP1(OD1)	Heavy	ALA2(N)	MD1/MD4/LMD-W	2.69 / 2.73 / 2.67
	LYS57(NZ)	Heavy	GLU3(OE1)	MD6	2.77
	TYR59(OH)	Heavy	GLU3(OE1)	MD6	2.66
	TYR59(OH)	Heavy	PHE4(N)	MD6	3.02
	LYS65(NZ)	Heavy	GLU3(OE2)	MD1/MD2/MD5/LMD-W/LMD-M	2.57 / 2.74 / 2.71 / 2.77 / 2.54

	LYS65(NZ)	Heavy	GLU3(OE1)	MD3	2.70
	ARG105(NH1)	Heavy	ARG5(O)	MD2/MD4	2.78 / 2.68
	ARG105(NH1)	Heavy	ASP7(OD1)	MD4	2.93
	PRO108(O)	Heavy	HIS6(NE2)	MD1/MD2/MD3/MD4/MD5/MD6/LMD -W/LMD-M	2.83 / 2.74 / 2.90 / 3.03 / 2.84 / 2.87 / 2.79 / 3.14
Hydrogen Bond	GLN27(OE1)	Light	ARG5(NH2)	MD3	2.87
	TYR92(O)	Light	HIS6(N)	MD2/MD3/MD4/MD5/MD6/LMD-W/LMD-M	2.98 / 3.26 / 3.10 / 3.18 / 2.88 / 3.18 / 2.84
	TYR92(OH)	Light	APS7(OD2)	LMD-M	2.69
	SER93(OG)	Light	ALA2(N)	MD1	2.70
	SER93(OG)	Light	PHE4(O)	MD1/MD5	2.79 / 2.75
	SER93(OG)	Light	ARG5(NE)	MD3	2.86
	SER93(OG)	Light	ARG5(NH1)	MD3	2.79
	THR94(O)	Light	ALA2(N)	MD1	2.62
	THR94(OG1)	Light	GLU3(N)	MD3/MD4	3.02 / 3.22
	THR94(OG1)	Light	PHE4(N)	MD1/MD2/MD5/LMD-W/LMD-M	3.06 / 3.28 / 3.06 / 3.22 / 3.01
	THE94(N)	Light	PHE4(O)	MD1/MD2/MD3/MD4/LMD-W/LMD-M	3.03 / 3.34 / 3.07 / 3.11 / 3.10 / 2.79

Table S2 – Description of nonbonded interactions calculated on the Aducanumab::$A\beta_{2-7}$ interface comprise hydrophobic, salt bridge, and hydrogen bond contacts. These interactions were determined in the representative conformations of MD2(Wild) conformational ensemble (#0-#8).

**Interaction**	**Adu Residue**	**Adu Chain**	**Aβ Residue**	**Sample (MD2)**	**Distance (Å)**
	TRP52	Heavy	PHE4	#1/#2/#4/#7/#8	
	TYR59	Heavy	ALA2	#3/#5	
	TYR59	Heavy	GLU3	#1/#2/#8	
	TYR59	Heavy	PHE4	#0/#1/#2/#3/#4/#5/#7	
	LYS65	Heavy	GLU3	#0/#1/#2/#3/#4/#5/#6/#7/#8	
	ILE102	Heavy	HIS6	#0/#1/#2/#3/#4/#5/#6/#7/#8	
	ALA104	Heavy	ASP7	#8	
	ARG105	Heavy	PHE4	#2/#3/#4/#5/#7/#8	
	ARG105	Heavy	ARG5	#2/#3#7	
	ARG105	Heavy	HIS6	#3/#7	
	ARG105	Heavy	ASP7	#2/#5/#7	
	GLY107	Heavy	PHE4	#0/#1/#2/#5/#7/#8	
	GLY107	Heavy	HIS6	#0/#1/#2/#3/#4/#5/#6/#7/#8	
Hydrophobic	PRO108	Heavy	PHE4	#2	
	PRO108	Heavy	HIS6	#0/#1/#2/#3/#4/#6/#7/#8	
	ASP1	Light	ALA2	#0/#1/#2/#4	
	TYR32	Light	HIS6	#0/#1/#2/#3/#4/#5/#6/#8	
	SER91	Light	HIS6	#0/#1/#2/#3/#4/#5/#6/#7/#8	
	TYR92	Light	ARG5	#0/#1/#2/#3/#4/#5/#6/#7/#8	
	TYR92	Light	HIS6	#1/#2/#3/#4/#5/#6/#7/#8	
	TYR92	Light	ASP7	#3/#4	
	SER93	Light	ALA2	#0/#1/#7	
	SER93	Light	PHE4	#0/#1/#2/#3/#4/#5/#6/#7/#8	

	SER93	Light	ARG5	#0/#3/#4/#6/#7/#8	
	THR94	Light	ALA2	#0/#1/#2/#4/#7/#8	
	THR94	Light	GLU3	#1/#2/#3/#4/#5/#6/#7/#8	
	THR94	Light	PHE4	#0/#1/#3/#4/#5/#7	
	PRO95	Light	GLU3	#3	
Salt Bridge	LYS65(NZ)	Heavy	GLU3(OE1)	#2	
	ARG105(NH1)	Heavy	ASP7(OD2)	#3	
	ARG105(NH2)	Heavy	ASP7(OD1)	#0	
Hydrogen Bond	ASP1(OD1)	Heavy	ALA2(N)	#0/#5	2.67/2.66
	ASP1(OD2)	Heavy	ALA2(N)	#1/#2	2.64/2.71
	LYS65(NZ)	Heavy	GLU3(OE2)	#3/#6/#7	2.63/2.72/2.68
	LYS65(NZ)	Heavy	GLU3(OE1)	#0/#1/#4/#5/#7	2.70/2.65/2.57/2.65/2.60
	ARG105(NH1)	Heavy	ARG5(O)	#2/#3/#7	2.64/2.71/2.87
	ARG105(NH1)	Heavy	ASP7(OD2)	#5	2.71
	ARG105(NH2)	Heavy	ASP7(OD2)	#2	2.87
	ARG105(NE)	Heavy	ASP7(OD1)	#5/#7	2.97/2.88
	PRO108(O)	Heavy	HIS6(NE2)	#0/#1/#2/#3/#4/#5/#6/#7/#8	2.64/2.75/2.85/2.64/2.63/3.02/2.70/2.68/2.96
	GLN27(OE1)	Light	ARG5(NH1)	#3	3.06
	TYR92(OH)	Light	ARG5(NH2)	#6	3.01
	TYR92(O)	Light	HIS6(N)	#0/#1/#2/#3/#4/#5/#6/#7/#8	3.03/3.13/2.94/2.89/3.31/3.05/3.33/2.94/3.32
	TYR92(OH)	Light	ASP7(OD1)	#4	3.18
	THR94(OG1)	Light	GLU3(OE1)	#3/#5/#6	2.59/2.68/2.79
	THR94(OG1)	Light	PHE4(N)	#0/#1/#2/#3/#4/#5/#6/#7/#8	3.25/3.31/3.27/2.95/2.88/2.92/3.05/3.00/3.11
	THR94(N)	Light	PHE4(O)	#0/#1/#2/#3/#4/#5/#6/#7	2.91/3.31/3.02/3.02/2.89/3.05/2.99/2.89

Table S3 – Description of nonbonded interactions calculated on the Aducanumab::A$\beta_{2\text{-}7}$ interface comprise hydrophobic, salt bridge, and hydrogen bond contacts. These interactions were determined in the representative conformations of MD6(Wild) conformational ensemble (#0-#7).

**Interaction**	**Adu Residue**	**Adu Chain**	**Aβ Residue**	**Sample (MD6)**	**Distance (Å)**
	TRP52	Heavy	PHE4	#1/#2/#3/#4/#6/#7	
	LYS57	Heavy	ALA2	#3	
	LYS57	Heavy	GLU3	#0/#3/#6	
	LYS57	Heavy	PHE4	#0/#1/#2/#3/#6	
	TYR59	Heavy	GLU3	#0/#3/#4/#6	
	TYR59	Heavy	PHE4	#1/#2/#4/#5	
	ILE102	Heavy	HIS6	#0/#1/#2/#3/#4/#5/#6/#7	
	ARG105	Heavy	PHE4	#0/#2/#3/#4/#6	
	ARG105	Heavy	ARG5	#1/#3	
	ARG105	Heavy	HIS6	#5	
	ARG105	Heavy	ASP7	#1/#2/#4/#5	
	GLY107	Heavy	PHE4	#4	

	GLY107	Heavy	HIS6	#0/#1/#2/#3/#4/#5/#6/#7	
Hydrophobic	PRO108	Heavy	HIS6	#0/#1/#2/#3/#4/#5/#6/#7	
	TYR32	Light	HIS6	#0/#1/#2/#3/#4/#5/#6/#7	
	TYR32	Light	ASP7	#0/#1/#2/#3/#5/#6	
	TYR91	Light	HIS6	#0/#1/#2/#3/#4/#5/#6/#7	
	TYR92	Light	ARG5	#1/#2/#3/#4/#5/#7	
	TYR92	Light	HIS6	#1/#2/#3/#4/#6/#7	
	TYR92	Light	ASP7	#6	
	SER93	Light	ALA2	#1	
	SER93	Light	GLU3	#2/#5	
	SER93	Light	PHE4	#4/#7	
	SER93	Light	ARG5	#4/#7	
	THR94	Light	GLU3	#7	
	THR94	Light	PHE4	#7	
Salt Bridge	LYS57(NZ)	Heavy	GLU3(OE2)	#0	
	LYS57(NZ)	Heavy	GLU3(OE1)	#3/#6	2.91/2.68
	TYR59(OH)	Heavy	GLU3(OE1)	#0/#6	2.74/2.44
	TYR59(OH)	Heavy	GLU3(OE2)	#3	2.56
	TYR59(OH)	Heavy	PHE4(N)	#0/#3/#6	3.30/3.05/3.20
	ARG105(NH1)	Heavy	ARG5(O)	#1/#2/#4/#5	2.72/3.04/3.33/2.93
	ARG105(NE)	Heavy	ASP7(OD1)	#4	2.95
	ARG105(NH1)	Heavy	ASP7(OD1)	#1/#5	2.79/2.66
	ARG105(NH1)	Heavy	ASP7(OD2)	#1/#2/#4	2.72/2.85/2.92
	ARG105(NH2)	Heavy	ASP7(OD2)	#2/#5	2.82/2.77
Hydrogen Bond	PRO108(O)	Heavy	HIS6(NE2)	#0/#1/#2/#3/#4/#5/#6/#7	3.01/2.83/2.79/2.87/2.63/2.90/2.98/2.96
	TYR32(OH)	Light	ASP7(O)	#1/#3	2.57/2.91
	TYR32(OH)	Light	ASP7(OXT)	#0/#2/#5/#6	2.63/2.84/2.63/2.76
	TYR92(OH)	Light	ARG5(NH1)	#4	2.88
	TYR92(O)	Light	HIS6(N)	#3/#4/#6/#7	3.25/2.93/3.25/2.97
	TYR92(OH)	Light	ASP7(O)	#6	2.73
	SER93(OG)	Light	GLU3(OE1)	#1/#2	2.91/2.77
	SER93(OG)	Light	GLU3(OE2)	#5	2.89
	SER93(OG)	Light	PHE4(O)	#7	2.80
	THR94(OG1)	Light	PHE4(N)	#4	3.23
	THR94(N)	Light	PHE4(O)	#4	3.07

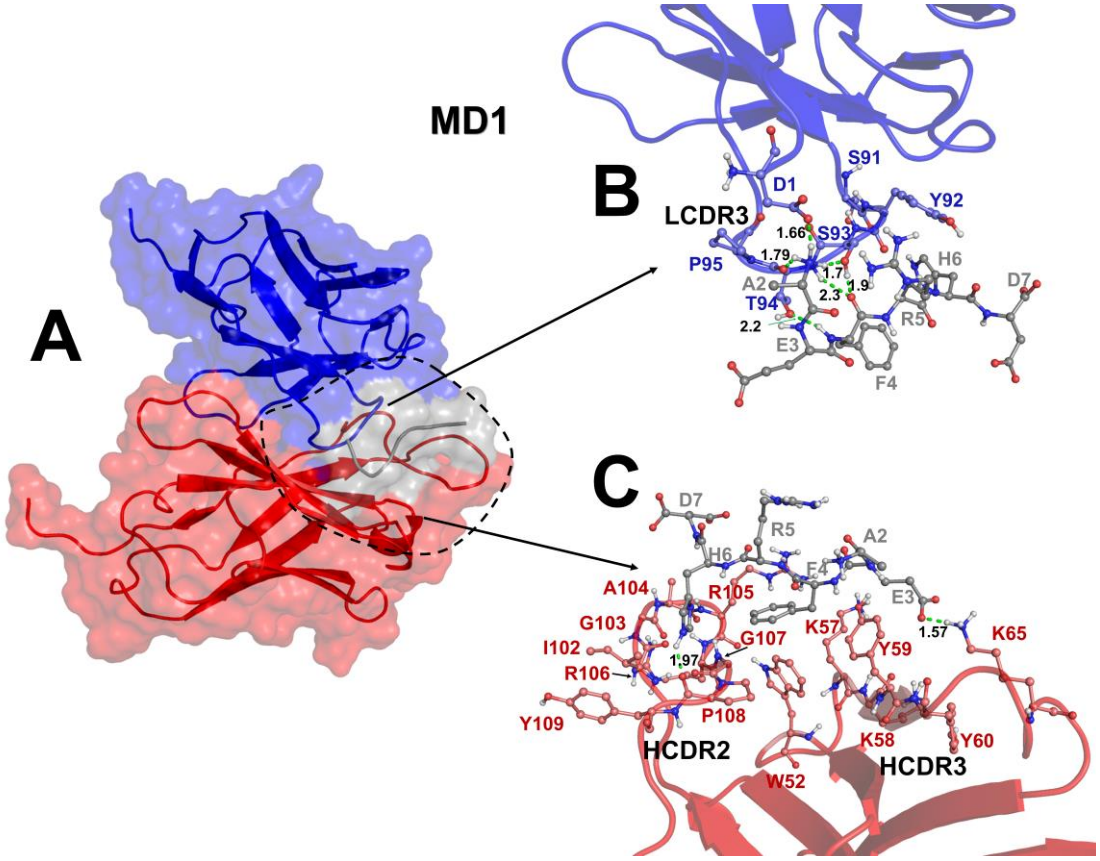

Figure S6 – Tridimensional illustration of final structural conformation MD1 of Aducanumab::Aβ_{2-7}. (A) Overview of the shortened version of Aducanumab bound to Aβ_{2-7}. (B) Critical contacts between LCDR3 and Aβ_{2-7}. (C) Critical contacts of Aβ_{2-7} with HCDR2 and HCDR3. Aducanumab's heavy chain and light chain are colored in red and blue, respectively, and Aβ2-7 is colored in grey.

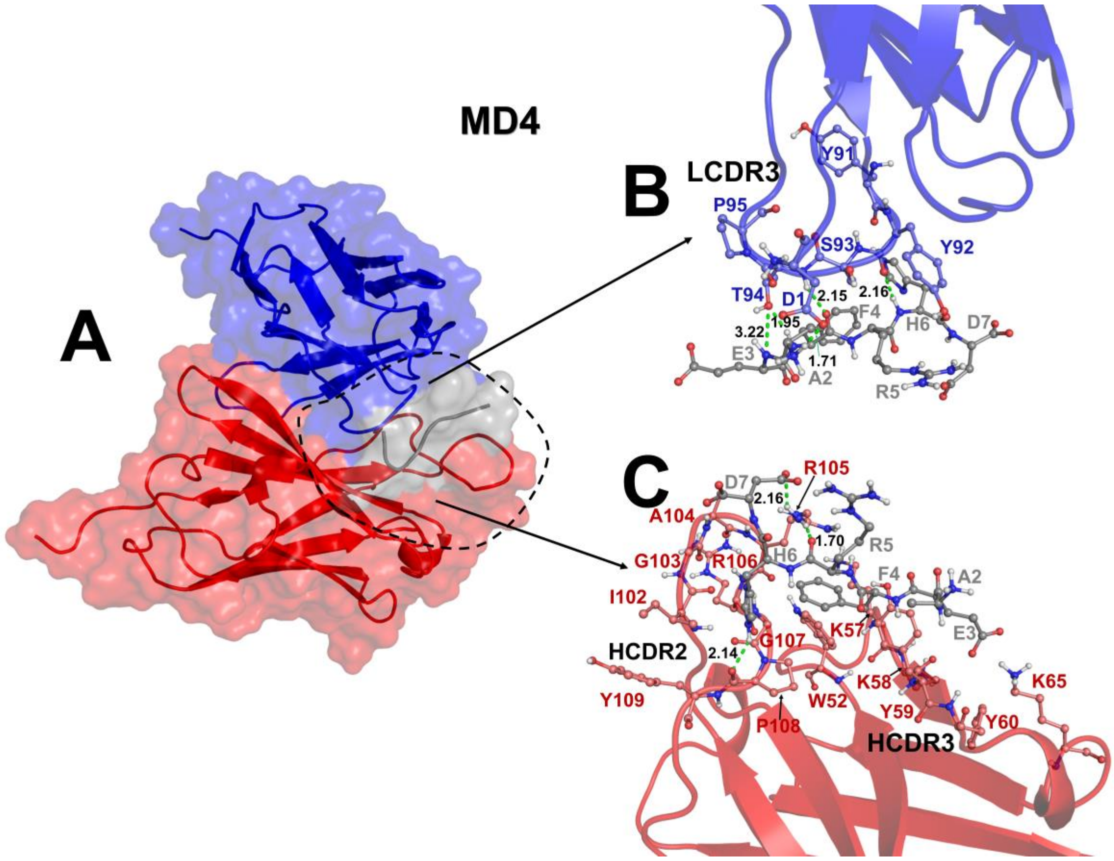

Figure S7 – Tridimensional illustration of final structural conformation MD1 of Aducanumab::A$\beta_{2\text{-}7}$. (A) Overview of the shortened version of Aducanumab bound to A$\beta_{2\text{-}7}$. (B) Critical contacts between LCDR3 and A$\beta_{2\text{-}7}$. (C) Critical contacts of A$\beta_{2\text{-}7}$ with HCDR2 and HCDR3. Aducanumab's heavy chain and light chain are colored in red and blue, respectively, and Aβ2-7 is colored in grey.

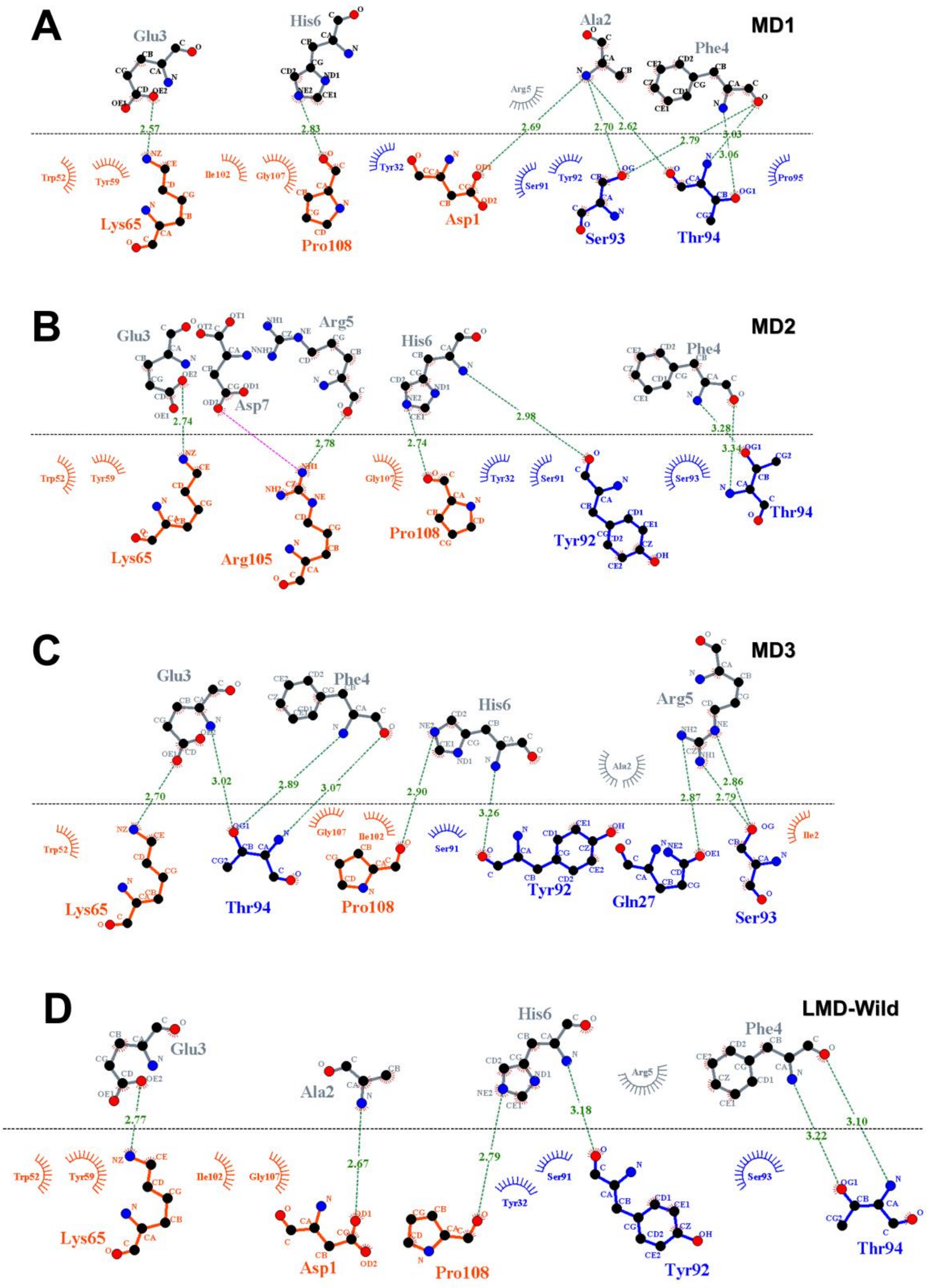

Figure S8 – Schematic representation of hydrogen bonds (dashed green lines), salt bridges (dashed magenta lines) and hydrophobic interactions (spoked arcs) detected between A$\beta_{2\text{-}7}$ (grey residues) and Heavy chain (orange residues) or Light chain (blue residues). These interactions were detected on FC (A) MD1, (B) MD2, (C) MD3, and (D) LMD-Wild.

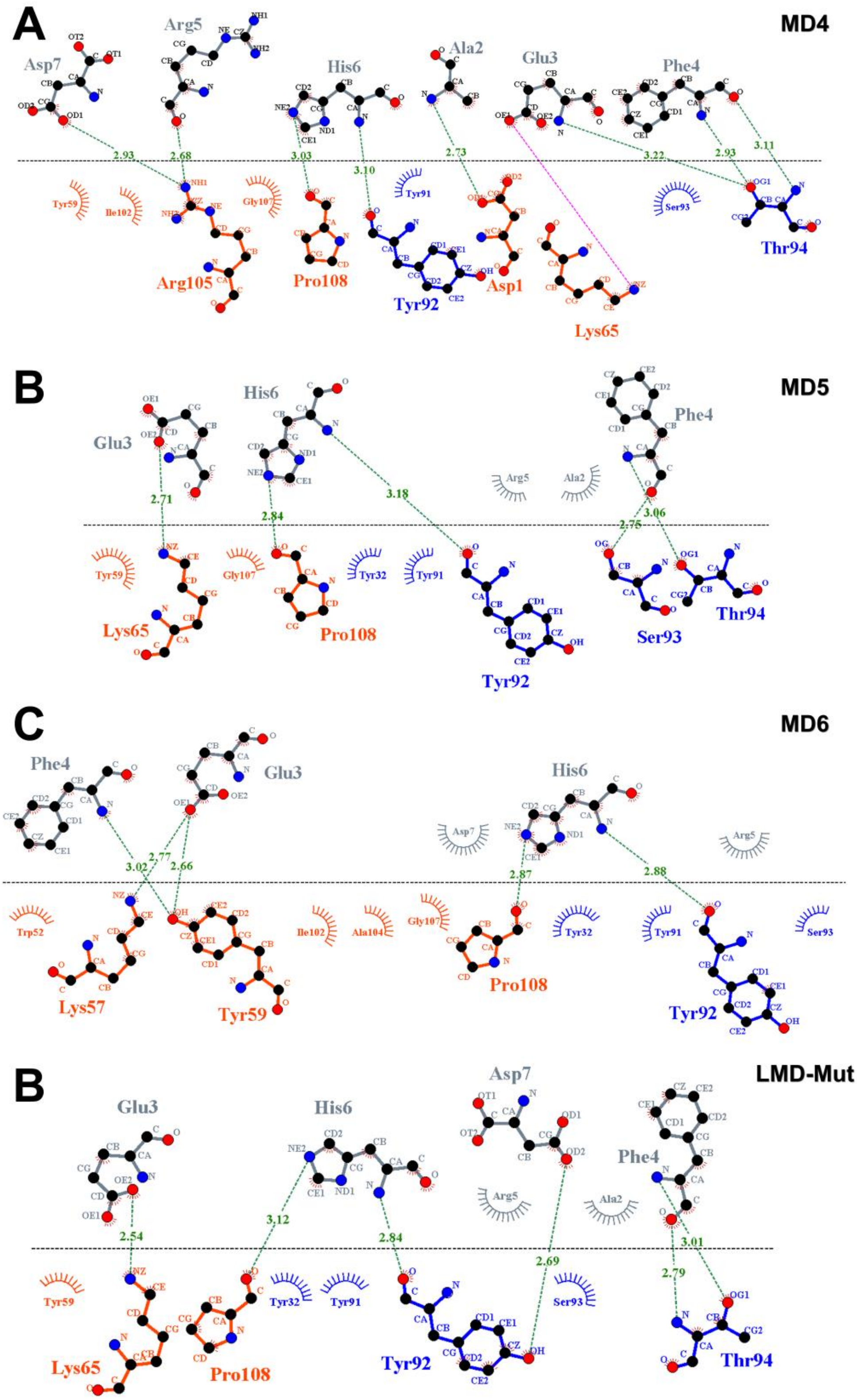

Figure S9 – Schematic representation of hydrogen bonds (dashed green lines), salt bridges (dashed magenta lines) and hydrophobic interactions (spoked arcs) detected between A$\beta_{2\text{-}7}$ (grey residues) and Heavy chain (orange residues) or Light chain (blue residues). These interactions were detected on FC (A) MD4, (B) MD5, and (C) MD6.

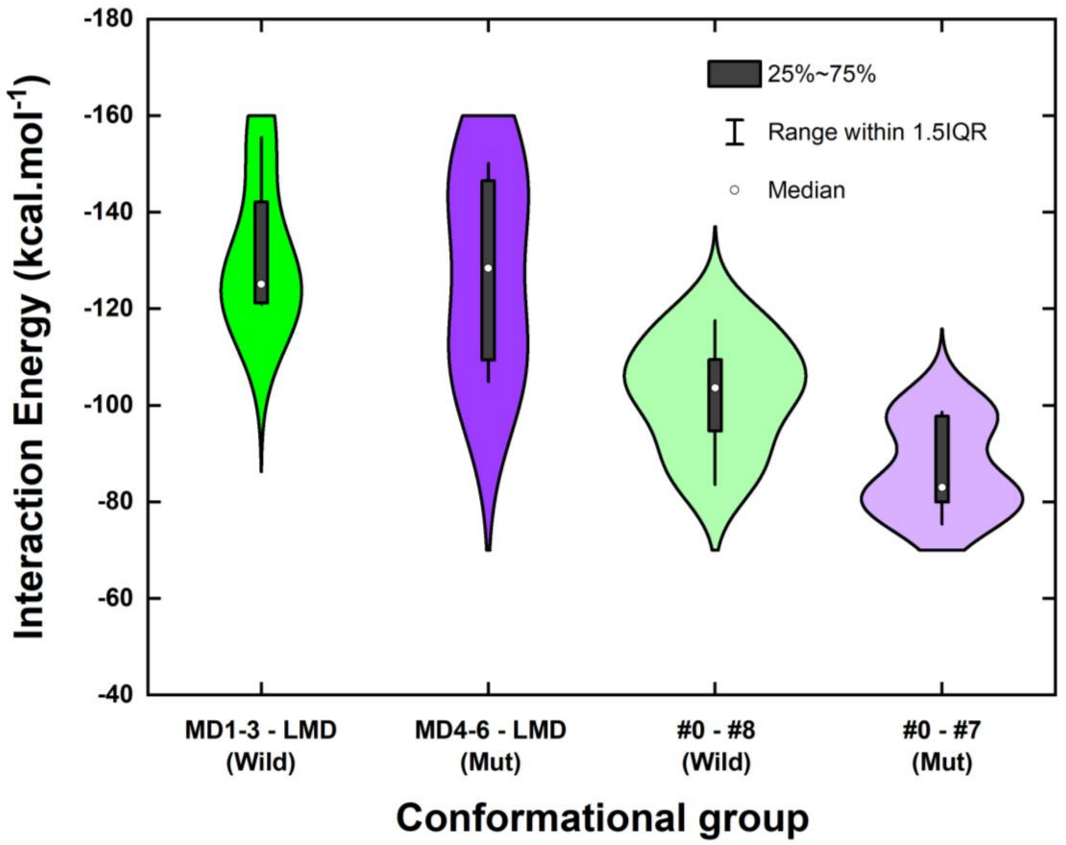

Figure S10 – Violin plot representing the distribution of interaction energy values within distinct conformational ensembles, namely Wild-FC, Mut-FC, Wild-RC, and Mut-RC. The distributions of values derived from wild and mutated complexes are colored in green and purple, respectively.

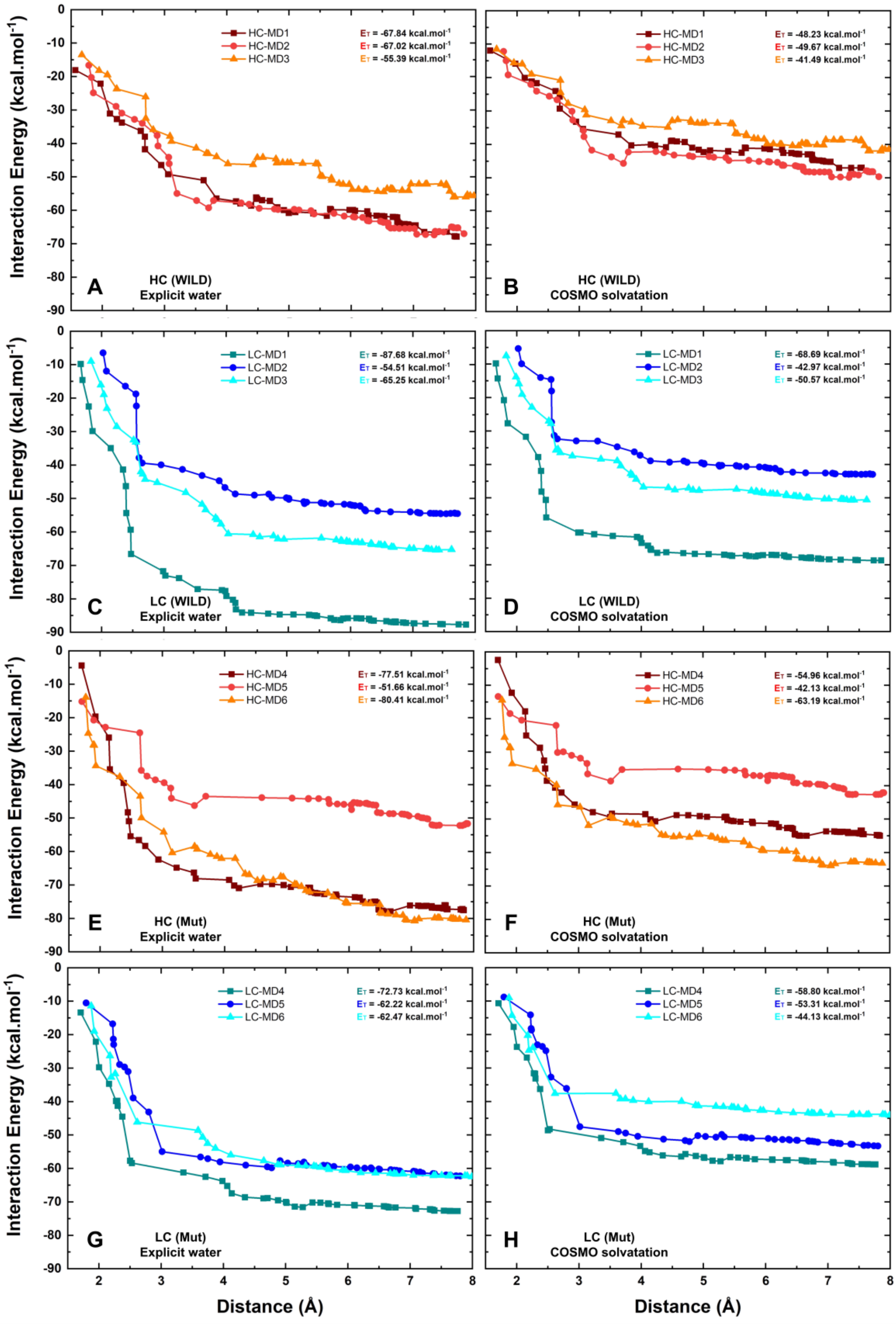

Figure S11 – Impact of explicit solvation on the calculation of interaction energy. Sum of the interaction energy as the interaction radius increases on surfaces (A-B) HC::A$\beta_{2\text{-}7}$ and (C-D) LC::A$\beta_{2\text{-}7}$ of FC MD1-MD3. Same for (E-F) HC::A$\beta_{2\text{-}7}$ and (G-H) LC::A$\beta_{2\text{-}7}$ of FC MD1-MD3. The charts displayed in the left and right columns illustrate the

quantum biochemistry results obtained through the employment of an explicit water and COSMO solvation model, respectively.

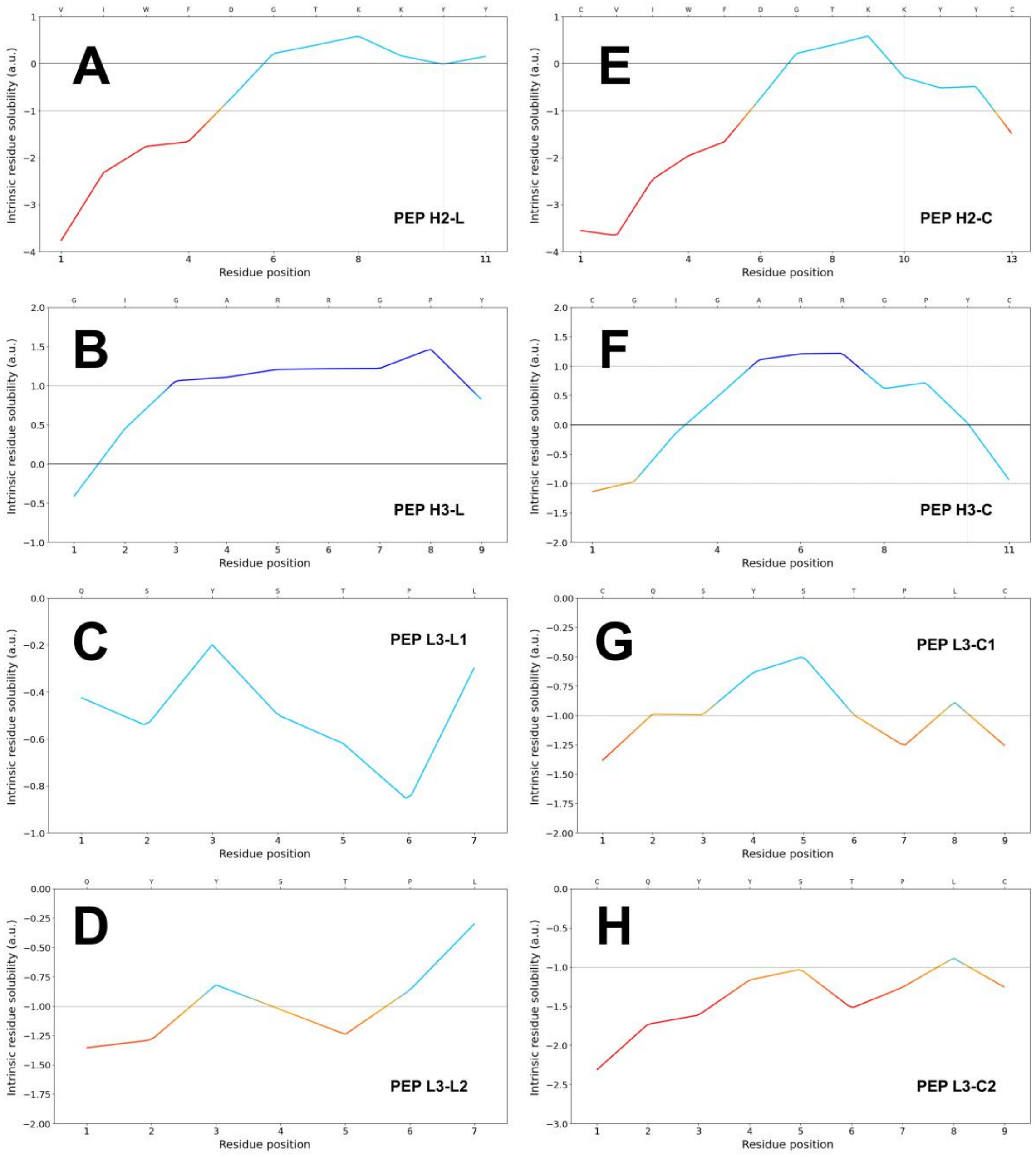

Figure S12 – Solubility of mimetic peptides (A) PEP-H2-L, (B) PEP-H3-L, (C) PEP-L3-L1, (D) PEP-L3-L2, (E) PEP-H2-C, (F) PEP-H3-C, (G) PEP-L3-C1, and (H) PEP-L3-C2 based on its amino acid sequences calculated using CAMSOL. Values higher than -1.0 suggest reasonable solubility.

Table S4 – Quantum biochemistry details of FC MD1.

Adu Residue	Adu Chain	Aβ Residue	Distance (Å)	Interaction Energy (kcal/mol)	Adu Residue Charge	Aβ Residue Charge	Adu Atom	Aβ Atom
LYS65	Heavy	GLU3	1.57	-18.05	1	-1	HZ1	OE2
PRO108	Heavy	HIS6	1.97	-4.05	0	0	O	HE2
TYR59	Heavy	GLU3	2.12	-8.95	0	-1	HE1	HB2
GLY107	Heavy	HIS6	2.24	-1.63	0	0	O	HE1
GLY107	Heavy	PHE4	2.32	-1.07	0	0	HA1	HE1
ILE102	Heavy	HIS6	2.62	-2.48	0	0	HG23	HE2
PRO108	Heavy	PHE4	2.68	-1.73	0	0	HD1	HE1
TRP52	Heavy	PHE4	2.69	-3.77	0	0	HZ2	CZ
TYR59	Heavy	PHE4	2.95	-4.71	0	0	CE2	HB1
ARG105	Heavy	PHE4	3.06	-2.76	1	0	HB1	HZ
TYR109	Heavy	HIS6	3.63	-1.78	0	0	HA	HE2
ARG105	Heavy	ASP7	3.84	-5.47	1	-2	HD2	OD2
TYR60	Heavy	GLU3	4.15	-0.85	0	-1	O	OE2
ARG106	Heavy	HIS6	4.22	-0.63	1	0	C	HE1
LYS57	Heavy	PHE4	4.39	-0.63	1	0	HG1	HE2
ASP62	Heavy	GLU3	4.47	2.40	-1	-1	OD1	OE2
ARG106	Heavy	PHE4	4.49	-0.40	1	0	C	HZ
GLY103	Heavy	HIS6	4.57	-0.40	0	0	HA1	HE1
GLY101	Heavy	HIS6	4.69	-0.20	0	0	HA2	HE1
ASP62	Heavy	ALA2	4.81	-1.92	-1	1	OD2	HB2
ALA104	Heavy	ASP7	4.92	-0.82	0	-2	HB3	OD2
ARG105	Heavy	HIS6	5.00	-0.87	1	0	HB1	HA
ARG105	Heavy	ARG5	5.10	0.28	1	1	HH22	O
VAL50	Heavy	PHE4	5.39	-0.22	0	0	HG13	HB2
ILE102	Heavy	PHE4	5.39	-0.18	0	0	O	HZ
GLY103	Heavy	ASP7	5.61	-0.73	0	-2	HA1	HN
LYS65	Heavy	ALA2	5.66	2.04	1	1	HZ2	HB3
TRP52	Heavy	HIS6	5.67	-0.18	0	0	HZ2	HE1
TRP47	Heavy	PHE4	5.97	-0.11	0	0	HH2	HB2
THR61	Heavy	GLU3	6.03	0.02	0	-1	HA	OE2
PRO108	Heavy	ARG5	6.06	-0.28	0	1	HD1	HA
TRP52	Heavy	ARG5	6.26	-0.15	0	1	HH2	HN
ARG105	Heavy	GLU3	6.28	-1.21	1	-1	HH21	O
GLY103	Heavy	PHE4	6.28	-0.05	0	0	HA1	HZ
TRP47	Heavy	GLU3	6.40	-0.05	0	-1	HZ3	OE2
LYS58	Heavy	PHE4	6.47	-0.23	1	0	O	HB1
TYR59	Heavy	ALA2	6.50	0.13	0	1	HD1	HB2
GLY107	Heavy	ARG5	6.52	-0.11	0	1	HA1	HA
GLY107	Heavy	ASP7	6.53	0.00	0	-2	HN	HN

ALA104	Heavy	HIS6	6.67	-0.16	0	0	N	HE1
THR61	Heavy	ALA2	6.71	-0.02	0	1	HA	HB2
TYR110	Heavy	HIS6	6.74	-0.15	0	0	N	HE2
LYS58	Heavy	GLU3	6.74	-1.13	1	-1	O	OE1
TYR59	Heavy	ARG5	6.74	-0.15	0	1	OH	HN
ASP99	Heavy	HIS6	6.75	-0.24	-1	0	OD2	HE2
TYR60	Heavy	ALA2	6.76	-0.25	0	1	O	HB2
ALA104	Heavy	PHE4	6.77	0.00	0	0	C	HZ
ILE102	Heavy	ASP7	6.85	-0.18	0	-2	O	HN
TYR109	Heavy	PHE4	6.91	-0.10	0	0	N	HE1
TRP47	Heavy	ALA2	6.92	-0.10	0	1	HZ3	HB2
LYS65	Heavy	PHE4	6.96	-0.21	1	0	HZ1	HN
GLY101	Heavy	PHE4	7.03	-0.03	0	0	HA2	HE1
ARG106	Heavy	ASP7	7.17	-1.97	1	-2	HN	HN
TRP52	Heavy	GLU3	7.37	-0.02	0	-1	HH2	C
ARG100	Heavy	HIS6	7.51	0.09	1	0	O	HE1
VAL50	Heavy	GLU3	7.53	0.01	0	-1	HG11	HB1
LYS57	Heavy	GLU3	7.66	-1.32	1	-1	HD2	HB2
TYR60	Heavy	PHE4	7.67	-0.09	0	0	HN	HB1
HIS35	Heavy	PHE4	7.69	-0.03	0	0	HE1	HD1
ASP1	Light	ALA2	1.66	-9.78	0	1	OD1	H1
SER93	Light	ALA2	1.69	-4.78	0	1	OG	H2
THR94	Light	ALA2	1.79	-7.93	0	1	O	H3
SER93	Light	PHE4	1.86	-7.34	0	0	HG1	O
THR94	Light	PHE4	2.15	-5.08	0	0	OG1	HN
SER93	Light	ARG5	2.34	-6.43	0	1	HB2	HB2
PRO95	Light	ALA2	2.39	-4.94	0	1	HD2	HB2
TYR92	Light	ARG5	2.40	-8.04	0	1	HH	HD1
TYR92	Light	HIS6	2.47	-5.00	0	0	HA	HD2
THR94	Light	GLU3	2.47	-7.25	0	-1	HG1	HB1
TYR32	Light	HIS6	2.99	-5.16	0	0	HE2	HB1
GLN27	Light	ARG5	3.02	-1.29	0	1	HE22	HH22
SER91	Light	HIS6	3.24	-0.75	0	0	O	HD2
ASP1	Light	ARG5	3.54	-3.28	0	1	OD1	HH11
ILE2	Light	ALA2	3.93	-0.30	0	1	HG11	H1
ILE2	Light	ARG5	3.98	-0.29	0	1	HD2	HH21
TYR92	Light	PHE4	4.01	-1.49	0	0	O	O
SER93	Light	HIS6	4.11	-1.11	0	0	HA	HN
THR94	Light	ARG5	4.15	-0.90	0	1	HN	HA
TYR32	Light	ASP7	4.16	-1.98	0	-2	HH	OT2
PRO95	Light	GLU3	4.26	-0.93	0	-1	HD2	HN
SER93	Light	GLU3	4.40	-0.05	0	-1	HG1	N
LEU96	Light	PHE4	4.67	-0.27	0	0	HD22	HD1
SER30	Light	HIS6	4.86	-0.26	0	0	HB2	HB1
GLN90	Light	ALA2	5.08	-0.01	0	1	HE22	H3
SER91	Light	PHE4	5.33	-0.11	0	0	O	HE1
PRO95	Light	PHE4	5.42	-0.16	0	0	N	HN
ILE29	Light	HIS6	5.46	-0.19	0	0	O	HD2

TYR92	Light	ASP7	5.68	-0.68	0	-2	HE1	N
GLN27	Light	ALA2	5.73	-0.46	0	1	OE1	H1
THR94	Light	HIS6	5.84	-0.09	0	0	HN	HN
TYR92	Light	ALA2	5.88	0.45	0	1	HE2	H2
SER91	Light	ARG5	5.95	0.13	0	1	O	HA
ASP1	Light	GLU3	6.09	0.00	0	-1	OD1	N
LEU96	Light	ARG5	6.17	-0.06	0	1	HD22	HA
LEU96	Light	ALA2	6.29	-0.11	0	1	HD21	H3
LEU96	Light	HIS6	6.30	-0.10	0	0	HD22	HE2
SER28	Light	ARG5	6.33	-0.39	0	1	O	HH22
GLN90	Light	PHE4	6.37	-0.07	0	0	HE22	O
TYR32	Light	ARG5	6.57	-0.16	0	1	HD2	HB1
ILE29	Light	ARG5	6.65	-0.23	0	1	HG21	HH22
ILE2	Light	PHE4	6.69	-0.07	0	0	HD2	O
SER30	Light	ASP7	6.71	0.23	0	-2	HB1	OT1
SER30	Light	ARG5	6.72	-0.06	0	1	HB2	HB1
PRO95	Light	ARG5	6.73	-0.22	0	1	HD1	HH11
ASN34	Light	HIS6	6.84	-0.03	0	0	HD22	HE2
THR97	Light	ALA2	6.84	-0.06	0	1	HN	H3
GLN90	Light	HIS6	6.89	-0.10	0	0	OE1	HD2
GLN90	Light	ARG5	7.01	-0.10	0	1	OE1	HA
ASP1	Light	PHE4	7.02	-0.03	0	0	OD1	O
SER31	Light	HIS6	7.20	-0.09	0	0	C	HD2
LEU33	Light	HIS6	7.44	-0.09	0	0	N	HD2
ILE2	Light	HIS6	7.48	-0.04	0	0	HD2	HN
TYR32	Light	PHE4	7.73	-0.07	0	0	HB2	HE1
TYR92	Light	GLU3	7.85	-0.05	0	-1	O	C

Table S5 – Quantum biochemistry details of FC MD2.

Adu Residue	Adu Chain	Aβ Residue	Distance (Å)	Interaction Energy (kcal/mol)	Adu Residue Charge	Aβ Residue Charge	Adu Atom	Aβ Atom
LYS65	Heavy	GLU3	1.79	-16.61	1	-1	HZ1	OE2
ARG105	Heavy	ARG5	1.82	-3.66	1	1	HH11	O
PRO108	Heavy	HIS6	1.86	-4.59	0	0	O	HE2
TYR59	Heavy	GLU3	2.22	-4.04	0	-1	HE1	HB2
GLY107	Heavy	HIS6	2.31	-2.07	0	0	HA1	HE1
GLY107	Heavy	PHE4	2.52	-1.83	0	0	HA1	HZ
ILE102	Heavy	HIS6	2.64	-1.17	0	0	HG22	HE1
ARG105	Heavy	PHE4	2.88	-3.67	1	0	HB1	HE2
ARG105	Heavy	HIS6	2.90	-3.11	1	0	HH12	HA
TRP52	Heavy	PHE4	3.07	-3.40	0	0	HZ2	HE2
PRO108	Heavy	PHE4	3.08	-1.91	0	0	HD1	HE1
ARG105	Heavy	ASP7	3.20	-8.88	1	-2	NH1	OD2
TYR109	Heavy	HIS6	3.51	-2.14	0	0	HA	HE2
TYR59	Heavy	PHE4	3.71	-2.17	0	0	CZ	HB1
ASP62	Heavy	GLU3	3.79	2.16	-1	-1	OD1	OE2

TYR60	Heavy	GLU3	4.23	-0.68	0	-1	O	OE2
LYS57	Heavy	PHE4	4.36	-0.42	1	0	HD1	HD2
ALA104	Heavy	ASP7	4.52	-1.23	0	-2	HB2	OD1
GLY101	Heavy	HIS6	4.77	-0.17	0	0	HA2	HE1
ARG106	Heavy	PHE4	4.82	-0.18	1	0	C	HZ
ARG106	Heavy	HIS6	5.09	0.05	1	0	C	HE1
VAL50	Heavy	PHE4	5.16	-0.19	0	0	HG12	HB2
ILE102	Heavy	PHE4	5.35	-0.19	0	0	O	HZ
ARG105	Heavy	GLU3	5.40	-0.97	1	-1	HH21	O
GLY103	Heavy	HIS6	5.72	0.15	0	0	N	HE1
GLY103	Heavy	ASP7	5.88	-0.82	0	-2	HA1	HB1
TRP47	Heavy	GLU3	6.03	-0.19	0	-1	HH2	OE2
TRP52	Heavy	HIS6	6.06	-0.11	0	0	HZ2	HE1
GLY107	Heavy	ARG5	6.18	-0.08	0	1	HA1	O
LYS58	Heavy	GLU3	6.26	-0.96	1	-1	O	HB2
THR61	Heavy	GLU3	6.27	0.11	0	-1	HA	OE2
GLY107	Heavy	ASP7	6.29	-0.23	0	-2	HA1	HN
TRP47	Heavy	PHE4	6.47	-0.09	0	0	HH2	HB2
TYR59	Heavy	ALA2	6.47	0.07	0	1	HE1	O
ILE102	Heavy	ASP7	6.48	-0.11	0	-2	HG23	HN
ASP99	Heavy	HIS6	6.52	-0.24	-1	0	OD2	HE2
TYR110	Heavy	HIS6	6.57	-0.13	0	0	N	HE2
TRP52	Heavy	ARG5	6.59	-0.08	0	1	HH2	HN
LYS57	Heavy	GLU3	6.60	-1.10	1	-1	HD1	O
PRO108	Heavy	ARG5	6.63	-0.41	0	1	HD1	HA
GLY103	Heavy	PHE4	6.69	-0.04	0	0	HA1	HZ
TYR109	Heavy	PHE4	6.77	-0.10	0	0	N	HE1
ALA104	Heavy	PHE4	6.86	0.03	0	0	C	HZ
GLY101	Heavy	PHE4	6.94	-0.01	0	0	HA1	HZ
TRP52	Heavy	GLU3	7.02	-0.06	0	-1	HH2	O
ASP62	Heavy	ALA2	7.05	-1.65	-1	1	OD2	HA
ALA104	Heavy	HIS6	7.19	-0.09	0	0	HN	ND1
LYS58	Heavy	PHE4	7.33	-0.10	1	0	O	HB1
ARG105	Heavy	ALA2	7.35	1.03	1	1	HH21	HB1
VAL50	Heavy	GLU3	7.41	0.00	0	-1	HG12	HB1
TYR59	Heavy	ARG5	7.48	-0.19	0	1	OH	HN
LYS65	Heavy	ALA2	7.61	1.55	1	1	HZ1	C
TYR60	Heavy	PHE4	7.63	-0.06	0	0	HN	HB2
LYS65	Heavy	PHE4	7.64	-0.22	1	0	HZ1	HN
HIS35	Heavy	HIS6	7.65	0.01	0	0	HE2	HE2
HIS35	Heavy	PHE4	7.69	-0.03	0	0	HE1	HD1
ARG100	Heavy	HIS6	7.71	0.03	1	0	C	HE1
ARG106	Heavy	ASP7	7.81	-1.78	1	-2	HN	OD1
TYR92	Light	HIS6	2.02	-6.48	0	0	HA	HD2
SER93	Light	ARG5	2.08	-5.45	0	1	HB1	HG1
THR94	Light	PHE4	2.38	-4.52	0	0	HG23	HB2
THR94	Light	GLU3	2.55	-2.31	0	-1	HG1	HB1
SER93	Light	PHE4	2.56	-3.60	0	0	HA	O

TYR92	Light	ARG5	2.56	-10.69	0	1	O	HA
TYR32	Light	HIS6	2.60	-4.75	0	0	HE2	HB1
SER91	Light	HIS6	2.65	-1.60	0	0	O	HD2
SER93	Light	ALA2	2.96	-0.57	0	1	HB2	HB1
THR94	Light	ALA2	3.30	-1.35	0	1	O	HB2
SER93	Light	HIS6	3.61	-1.83	0	0	HB1	HN
THR94	Light	ARG5	3.89	-1.57	0	1	HN	HA
ASP1	Light	ALA2	3.98	-1.98	0	1	OD2	HB3
TYR92	Light	PHE4	4.15	-1.93	0	0	O	HD1
LEU96	Light	PHE4	4.45	-0.37	0	0	HD23	HD1
TYR92	Light	ASP7	4.69	0.31	0	-2	HE1	OT1
TYR32	Light	ASP7	4.74	-1.00	0	-2	HH	OT2
GLN27	Light	ARG5	4.97	-0.13	0	1	HG2	HH12
ILE2	Light	ARG5	5.01	-0.15	0	1	HD1	HE
SER91	Light	PHE4	5.01	-0.29	0	0	O	HE1
PRO95	Light	GLU3	5.25	-0.73	0	-1	HD2	HN
PRO95	Light	ALA2	5.26	-0.54	0	1	HD2	HB2
SER93	Light	GLU3	5.29	0.39	0	-1	HB2	HN
ILE2	Light	ALA2	5.53	-0.12	0	1	HD1	HB3
LEU96	Light	HIS6	5.53	-0.12	0	0	HD21	HE2
SER30	Light	HIS6	5.58	-0.15	0	0	HB1	HB1
SER91	Light	ARG5	5.69	-0.09	0	1	O	HA
LEU96	Light	ARG5	5.90	-0.09	0	1	HD23	HA
THR94	Light	HIS6	5.99	-0.11	0	0	HN	HN
ILE29	Light	HIS6	6.02	-0.20	0	0	O	HD2
ILE29	Light	ARG5	6.07	-0.12	0	1	HG21	HG1
ASN34	Light	HIS6	6.15	-0.03	0	0	HD22	HE2
GLN90	Light	HIS6	6.15	-0.16	0	0	OE1	HD2
ASP1	Light	ARG5	6.22	-0.95	0	1	OD2	HH12
ASP1	Light	GLU3	6.23	-0.18	0	-1	OD2	HN
PRO95	Light	PHE4	6.24	-0.07	0	0	N	HN
TYR32	Light	ARG5	6.24	-0.16	0	1	HE2	HG2
TYR92	Light	ALA2	6.43	-0.03	0	1	O	HB1
SER28	Light	ARG5	6.64	-0.26	0	1	O	HG1
LEU96	Light	ALA2	6.96	-0.03	0	1	HD23	HB2
SER30	Light	ASP7	7.07	-0.12	0	-2	HB2	OT1
SER31	Light	HIS6	7.09	-0.14	0	0	HN	HB1
ILE2	Light	PHE4	7.10	-0.03	0	0	HD1	O
GLN90	Light	ARG5	7.11	-0.05	0	1	OE1	HA
TYR32	Light	PHE4	7.28	-0.07	0	0	HE2	HE1
GLN90	Light	PHE4	7.33	-0.05	0	0	HE22	O
ASP1	Light	PHE4	7.37	0.01	0	0	OD2	O
GLN90	Light	ALA2	7.44	0.01	0	1	HE22	HB2
ILE2	Light	HIS6	7.45	-0.09	0	0	HD1	HN
SER28	Light	HIS6	7.53	-0.02	0	0	O	HB1
GLN27	Light	ALA2	7.60	0.10	0	1	HG2	HB3
SER93	Light	ASP7	7.67	0.09	0	-2	HB1	N
LEU33	Light	HIS6	7.68	-0.07	0	0	N	HD2

LEU96	Light	GLU3	7.72	-0.06	0	-1	HD23	HB1

Table S6 – Quantum biochemistry details of FC MD3

Adu Residue	Adu Chain	Aβ Residue	Distance (Å)	Interaction Energy (kcal/mol)	Adu Residue Charge	Aβ Residue Charge	Adu Atom	Aβ Atom
LYS65	Heavy	GLU3	1.67	-13.47	1	-1	HZ2	OE1
PRO108	Heavy	HIS6	1.95	-4.71	0	0	O	HE2
ILE102	Heavy	HIS6	2.08	-1.29	0	0	HG21	HE1
ARG105	Heavy	PHE4	2.23	-4.19	1	0	HB1	HE2
GLY107	Heavy	PHE4	2.70	-2.40	0	0	HA1	CZ
TYR59	Heavy	GLU3	2.70	-6.41	0	-1	HE1	HB2
TRP52	Heavy	PHE4	2.82	-3.41	0	0	HH2	CG
PRO108	Heavy	PHE4	3.09	-1.94	0	0	HD1	HE1
GLY107	Heavy	HIS6	3.11	-1.53	0	0	O	HE1
TYR59	Heavy	PHE4	3.51	-2.01	0	0	HE2	HB1
TYR109	Heavy	HIS6	3.68	-1.58	0	0	HA	HE2
TYR60	Heavy	GLU3	3.70	-0.11	0	-1	O	OE1
GLY103	Heavy	HIS6	3.84	-0.91	0	0	HA1	HE1
ARG105	Heavy	GLU3	4.02	-2.09	1	-1	HH21	O
ILE102	Heavy	PHE4	4.42	-0.29	0	0	O	HZ
ASP62	Heavy	GLU3	4.49	2.12	-1	-1	OD1	OE1
ARG105	Heavy	ARG5	4.58	0.07	1	1	HH22	HN
ARG106	Heavy	PHE4	4.79	-0.47	1	0	HN	HZ
ALA104	Heavy	ASP7	4.81	-1.21	0	-2	HB1	OD1
GLY101	Heavy	HIS6	4.81	0.17	0	0	HA2	HE1
ARG106	Heavy	HIS6	4.90	-0.04	1	0	HA	HE1
GLY103	Heavy	PHE4	4.98	-0.07	0	0	HA1	HZ
THR61	Heavy	GLU3	5.03	-0.03	0	-1	HA	OE1
TRP47	Heavy	GLU3	5.22	-0.10	0	-1	HZ3	OE1
TRP52	Heavy	ARG5	5.43	-0.10	0	1	HH2	HN
VAL50	Heavy	PHE4	5.46	-0.12	0	0	HG12	HB2
ARG105	Heavy	ASP7	5.51	-3.49	1	-2	HG1	OD1
LYS57	Heavy	PHE4	5.54	-0.25	1	0	HG1	HB1
GLY103	Heavy	ASP7	5.68	-0.51	0	-2	HA1	HN
ARG105	Heavy	HIS6	5.69	-0.64	1	0	HB1	HA
LYS58	Heavy	GLU3	5.80	-0.96	1	-1	O	OE2
ALA104	Heavy	HIS6	5.87	-0.22	0	0	HN	ND1
ALA104	Heavy	PHE4	5.97	-0.05	0	0	C	HE2
LYS57	Heavy	GLU3	6.02	-1.56	1	-1	HE2	O
GLY107	Heavy	ARG5	6.12	0.04	0	1	HA1	HN
TRP52	Heavy	HIS6	6.22	-0.12	0	0	HZ2	HE1
TRP52	Heavy	GLU3	6.25	-0.09	0	-1	HH2	O
PRO108	Heavy	ARG5	6.42	-0.40	0	1	HD1	N
TYR109	Heavy	PHE4	6.52	-0.09	0	0	N	HE1
ARG105	Heavy	ALA2	6.57	0.91	1	1	HH21	O
TYR59	Heavy	ALA2	6.63	0.02	0	1	HH	O

TYR110	Heavy	HIS6	6.66	-0.08	0	0	N	HE2
ASP99	Heavy	HIS6	6.66	-0.25	-1	0	OD2	HE2
TRP47	Heavy	PHE4	6.68	-0.09	0	0	HH2	HB2
TYR59	Heavy	ARG5	6.82	-0.11	0	1	OH	HN
GLY101	Heavy	PHE4	6.92	-0.04	0	0	HA1	HZ
LYS65	Heavy	ALA2	6.96	2.01	1	1	HZ2	HA
ILE102	Heavy	ASP7	7.09	-0.06	0	-2	HG22	HN
HIS35	Heavy	PHE4	7.19	-0.03	0	0	HE1	HD1
VAL50	Heavy	GLU3	7.21	0.04	0	-1	HG13	HB1
HIS35	Heavy	HIS6	7.44	0.01	0	0	HE2	HE2
GLY103	Heavy	ARG5	7.52	-0.04	0	1	HA1	O
LYS65	Heavy	PHE4	7.53	-0.24	1	0	HZ2	HN
GLY107	Heavy	ASP7	7.54	-0.14	0	-2	HN	HN
ASP62	Heavy	ALA2	7.58	-1.76	-1	1	OD2	HA
ARG106	Heavy	ASP7	7.64	-1.78	1	-2	HN	HN
ARG100	Heavy	HIS6	7.66	0.06	1	0	C	HE1
ALA104	Heavy	ARG5	7.84	0.00	0	1	HB1	O
ARG106	Heavy	ARG5	7.86	0.63	1	1	HN	O
ILE102	Heavy	ARG5	7.91	-0.21	0	1	O	O
ALA49	Heavy	GLU3	7.97	-0.07	0	-1	HA	OE1
SER93	Light	ARG5	1.83	-8.99	0	1	OG	HH12
THR94	Light	PHE4	1.99	-7.04	0	0	OG1	HN
THR94	Light	GLU3	2.04	-2.88	0	-1	HG1	HN
GLN27	Light	ARG5	2.09	-4.16	0	1	OE1	HH21
TYR92	Light	HIS6	2.24	-5.43	0	0	HA	HD2
SER93	Light	PHE4	2.51	-4.00	0	0	HA	O
SER93	Light	ALA2	2.55	-0.64	0	1	HG1	HB2
TYR92	Light	ARG5	2.63	-8.85	0	1	O	HA
THR94	Light	ALA2	2.66	-0.73	0	1	HG1	HB1
ILE2	Light	ARG5	2.70	-1.56	0	1	HD3	HH12
SER91	Light	HIS6	2.89	-0.96	0	0	O	HD2
ASP1	Light	ALA2	3.35	-2.95	0	1	OD1	HB2
ASP1	Light	ARG5	3.61	-3.53	0	1	OD1	HH11
TYR92	Light	PHE4	3.67	-1.65	0	0	O	HE1
TYR32	Light	HIS6	3.83	-2.42	0	0	HE1	HB1
LEU96	Light	PHE4	3.86	-0.53	0	0	HD22	HD1
SER93	Light	HIS6	3.92	-1.33	0	0	HB2	HN
THR94	Light	ARG5	4.03	-2.92	0	1	HN	HA
SER91	Light	PHE4	4.43	-0.20	0	0	O	HE1
PRO95	Light	GLU3	4.54	-0.72	0	-1	HD2	HN
TYR92	Light	ASP7	4.76	0.30	0	-2	HE1	N
PRO95	Light	ALA2	4.83	-0.88	0	1	HD2	HA
ILE2	Light	ALA2	4.93	-0.13	0	1	HD3	HB2
SER93	Light	GLU3	5.53	0.39	0	-1	HG1	HN
SER28	Light	ARG5	5.77	-0.59	0	1	O	HH21
LEU96	Light	HIS6	5.85	-0.10	0	0	HD23	HD2
PRO95	Light	PHE4	5.86	-0.34	0	0	N	HN
TYR32	Light	ASP7	5.93	0.12	0	-2	HE1	OT1

GLN90	Light	ARG5	5.94	-0.20	0	1	HE22	HH12
THR94	Light	HIS6	5.98	-0.11	0	0	HN	HN
LEU96	Light	ARG5	6.07	-0.12	0	1	HD22	HA
ASP1	Light	GLU3	6.12	0.02	0	-1	OD1	HN
SER30	Light	HIS6	6.18	-0.11	0	0	HB1	HB1
SER91	Light	ARG5	6.19	0.00	0	1	O	HA
ILE29	Light	ARG5	6.33	-0.23	0	1	HG21	HH12
TYR92	Light	ALA2	6.33	-0.13	0	1	O	HB3
ILE29	Light	HIS6	6.34	-0.13	0	0	O	HB1
GLN27	Light	ALA2	6.45	-0.10	0	1	HE22	HB2
GLN90	Light	ALA2	6.48	-0.06	0	1	HE22	HB1
GLN90	Light	HIS6	6.54	-0.10	0	0	OE1	HD2
LEU96	Light	ALA2	6.55	-0.12	0	1	HD21	HB1
PRO95	Light	ARG5	6.65	-0.37	0	1	HD1	HH12
ILE2	Light	PHE4	6.66	-0.06	0	0	HD3	O
GLN90	Light	PHE4	6.69	-0.05	0	0	HE22	O
TYR32	Light	ARG5	6.94	-0.37	0	1	HD1	HG1
ASN34	Light	HIS6	7.01	-0.01	0	0	HD22	HE2
SER30	Light	ASP7	7.21	-0.17	0	-2	HB2	OT2
ASP1	Light	PHE4	7.22	-0.01	0	0	OD1	O
ILE2	Light	HIS6	7.25	-0.05	0	0	HD3	HN
TYR32	Light	PHE4	7.30	-0.08	0	0	CD2	HE1
SER31	Light	HIS6	7.37	-0.16	0	0	HN	HB1
SER26	Light	ARG5	7.37	0.13	0	1	HG1	HH11
LEU96	Light	GLU3	7.42	-0.06	0	-1	HG	HB1
SER93	Light	ASP7	7.62	0.11	0	-2	HB2	N

Table S7 – Quantum biochemistry details of FC LMD-Wild

Adu Residue	Adu Chain	Aβ Residue	Distance (Å)	Interaction Energy (kcal/mol)	Adu Residue Charge	Aβ Residue Charge	Adu Atom	Aβ Atom
LYS65	Heavy	GLU3	1.77	-15.48	1	-1	HZ2	OE2
PRO108	Heavy	HIS6	1.90	-5.48	0	0	O	HE2
ILE102	Heavy	HIS6	2.14	-0.87	0	0	HG23	HE1
TYR59	Heavy	GLU3	2.25	-6.91	0	-1	HE1	HB2
GLY107	Heavy	PHE4	2.51	-1.20	0	0	HA1	HZ
GLY107	Heavy	HIS6	2.67	-3.06	0	0	O	HE1
PRO108	Heavy	PHE4	2.74	-2.67	0	0	HD1	CE1
TYR59	Heavy	PHE4	2.78	-4.56	0	0	CZ	HB1
TRP52	Heavy	PHE4	2.92	-2.99	0	0	HH2	CD2
TYR60	Heavy	GLU3	3.71	-1.07	0	-1	O	OE2
TYR109	Heavy	HIS6	4.07	-1.84	0	0	HA	HE2
ARG105	Heavy	ASP7	4.09	-5.12	1	-2	HH22	OD1
GLY101	Heavy	HIS6	4.67	0.00	0	0	HA2	HE1
ASP62	Heavy	GLU3	4.78	1.48	-1	-1	OD2	OE2
ARG106	Heavy	HIS6	4.80	-0.52	1	0	C	HE1
ARG105	Heavy	PHE4	4.95	-0.22	1	0	HB1	HE2

VAL50	Heavy	PHE4	5.22	-0.20	0	0	HG12	HB2
LYS57	Heavy	PHE4	5.23	-0.25	1	0	HD2	HD2
TRP47	Heavy	GLU3	5.27	-0.29	0	-1	HH2	HB1
ILE102	Heavy	PHE4	5.42	-0.14	0	0	HG23	HZ
GLY103	Heavy	HIS6	5.44	-0.19	0	0	N	HE1
THR61	Heavy	GLU3	5.51	0.02	0	-1	HA	OE2
ARG106	Heavy	PHE4	5.71	-0.16	1	0	C	HZ
TYR59	Heavy	ARG5	6.00	-0.21	0	1	HH	HN
TRP52	Heavy	HIS6	6.11	-0.18	0	0	HH2	HE2
LYS58	Heavy	GLU3	6.28	-1.15	1	-1	O	HB2
TRP47	Heavy	PHE4	6.28	-0.14	0	0	HH2	HB2
ARG105	Heavy	HIS6	6.44	-0.43	1	0	HN	HE1
PRO108	Heavy	ARG5	6.44	-0.58	0	1	HD1	HA
TRP52	Heavy	ARG5	6.47	-0.13	0	1	HH2	HN
ASP99	Heavy	HIS6	6.50	-0.27	-1	0	OD2	HE2
TYR59	Heavy	ALA2	6.52	0.05	0	1	HE1	C
GLY107	Heavy	ARG5	6.59	-0.11	0	1	HA1	O
TYR109	Heavy	PHE4	6.67	-0.13	0	0	N	HE1
LYS58	Heavy	PHE4	6.69	-0.26	1	0	O	HB1
GLY107	Heavy	ASP7	6.82	-0.07	0	-2	HA1	HN
ILE102	Heavy	ASP7	6.85	-0.04	0	-2	HG21	HN
GLY101	Heavy	PHE4	6.87	-0.03	0	0	HA2	HZ
HIS35	Heavy	PHE4	6.87	-0.04	0	0	HE1	HE1
LYS57	Heavy	GLU3	6.99	-1.42	1	-1	HZ3	O
TYR110	Heavy	HIS6	7.00	-0.09	0	0	HN	HE2
ASP62	Heavy	ALA2	7.02	-1.68	-1	1	OD2	HA
LYS65	Heavy	ALA2	7.09	1.47	1	1	HZ3	HA
VAL50	Heavy	GLU3	7.09	0.05	0	-1	HG13	HB1
ALA104	Heavy	ASP7	7.12	-0.47	0	-2	HB2	HB1
HIS35	Heavy	HIS6	7.16	0.01	0	0	HE1	HE2
GLY103	Heavy	ASP7	7.22	-0.08	0	-2	HA1	OT1
ARG105	Heavy	ARG5	7.26	0.71	1	1	HH22	O
TYR60	Heavy	PHE4	7.30	-0.08	0	0	HN	HB2
LYS65	Heavy	PHE4	7.30	-0.24	1	0	HZ2	HN
ALA104	Heavy	HIS6	7.48	-0.06	0	0	N	HE1
TRP52	Heavy	GLU3	7.51	-0.02	0	-1	HH2	O
ARG100	Heavy	HIS6	7.56	0.03	1	0	C	HE1
LYS57	Heavy	ASP7	7.57	-2.38	1	-2	HZ2	OD1
TRP47	Heavy	ALA2	7.70	-0.10	0	1	HZ3	HA
LYS57	Heavy	ARG5	7.92	0.69	1	1	HZ3	HN
TRP52	Heavy	ASP7	7.99	-0.09	0	-2	HZ2	HN
ASP1	Light	ALA2	1.63	-13.97	0	1	OD1	H1
TYR92	Light	HIS6	2.07	-8.37	0	0	HA	HD2
THR94	Light	PHE4	2.16	-7.00	0	0	HN	O
SER93	Light	ARG5	2.20	-4.34	0	1	HB1	HA
SER93	Light	ALA2	2.36	-0.71	0	1	HB2	HB1
THR94	Light	GLU3	2.39	-4.95	0	-1	HG1	HN
SER93	Light	PHE4	2.45	-2.07	0	0	HB2	O

TYR32	Light	HIS6	2.49	-3.63	0	0	HE1	HB2
TYR92	Light	ARG5	2.54	-11.03	0	1	O	HA
THR94	Light	ALA2	2.64	-2.23	0	1	O	HB2
SER91	Light	HIS6	2.93	-0.61	0	0	O	HD2
TYR92	Light	PHE4	3.51	-1.94	0	0	O	HE1
SER93	Light	HIS6	3.59	-1.81	0	0	HB1	HN
LEU96	Light	PHE4	3.94	-0.55	0	0	HD21	HE1
THR94	Light	ARG5	3.99	-1.20	0	1	HN	HA
TYR92	Light	ASP7	4.22	-0.32	0	-2	HH	HA
PRO95	Light	ALA2	4.23	-0.71	0	1	HD2	HA
ASP1	Light	ARG5	4.26	-1.12	0	1	OD2	HB2
SER91	Light	PHE4	4.39	-0.30	0	0	O	HE1
PRO95	Light	GLU3	5.04	-0.86	0	-1	HD2	HN
ILE2	Light	ALA2	5.07	-0.44	0	1	HN	HB2
SER93	Light	GLU3	5.14	0.29	0	-1	HB2	HN
SER30	Light	HIS6	5.28	-0.29	0	0	HB2	HB2
LEU96	Light	HIS6	5.29	-0.11	0	0	HD21	HE2
TYR32	Light	PHE4	5.43	-0.12	0	0	HE1	HZ
ASP1	Light	GLU3	5.67	0.48	0	-1	OD1	HN
TYR32	Light	ASP7	5.68	-0.22	0	-2	OH	OT1
ASP1	Light	PHE4	5.80	-0.12	0	0	OD2	O
PRO95	Light	PHE4	5.96	-0.16	0	0	N	HN
THR94	Light	HIS6	6.07	-0.14	0	0	HN	HN
GLN90	Light	ALA2	6.08	-0.06	0	1	HE22	HB2
GLN90	Light	HIS6	6.11	-0.15	0	0	OE1	HD2
TYR92	Light	ALA2	6.17	-0.03	0	1	O	HB1
ILE2	Light	ARG5	6.28	-0.15	0	1	HG12	HB2
ILE29	Light	HIS6	6.31	-0.18	0	0	O	HB1
SER91	Light	ARG5	6.47	0.47	0	1	O	HA
GLN90	Light	PHE4	6.48	-0.11	0	0	HE22	O
TYR32	Light	ARG5	6.58	-0.09	0	1	HE1	C
SER28	Light	ARG5	6.62	-0.23	0	1	O	HH12
LEU96	Light	ARG5	6.66	-0.01	0	1	HD21	HA
GLN90	Light	ARG5	6.73	-0.14	0	1	OE1	HA
SER30	Light	ARG5	6.75	-0.14	0	1	HG1	HH12
SER30	Light	ASP7	6.78	0.15	0	-2	OG	OT1
GLN27	Light	ARG5	6.81	-0.01	0	1	HG1	HH12
ILE29	Light	ARG5	7.03	-0.18	0	1	HG22	HB1
LEU96	Light	ALA2	7.17	0.00	0	1	HG	HB1
ILE2	Light	PHE4	7.21	-0.06	0	0	HG12	O
SER93	Light	ASP7	7.35	0.09	0	-2	HB1	N
ASN34	Light	HIS6	7.39	-0.01	0	0	HD22	HE2
GLN3	Light	ALA2	7.49	-0.03	0	1	HE22	HB2
ILE2	Light	HIS6	7.62	-0.05	0	0	HG12	HN
LEU96	Light	GLU3	7.64	-0.07	0	-1	HG	HB1

Table S8 – Quantum biochemistry details of FC MD4

Adu Residue	Adu Chain	Aβ Residue	Distance (Å)	Interaction Energy (kcal/mol)	Adu Residue Charge	Aβ Residue Charge	Adu Atom	Aβ Atom
ARG105	Heavy	ARG5	1.70	-4.37	1	1	HH11	O
LYS65	Heavy	GLU3	1.93	-15.25	1	-1	HZ1	OE1
PRO108	Heavy	HIS6	2.14	-6.29	0	0	O	HE2
ARG105	Heavy	ASP7	2.16	-9.50	1	-2	HH12	OD1
GLY107	Heavy	HIS6	2.38	-4.10	0	0	HA1	HE1
TYR59	Heavy	GLU3	2.45	-8.76	0	-1	HE1	HB2
ILE102	Heavy	HIS6	2.46	-2.66	0	0	HG22	HE1
TYR59	Heavy	PHE4	2.49	-4.50	0	0	HH	HD2
ARG105	Heavy	PHE4	2.62	-1.13	1	0	HB1	HE2
GLY107	Heavy	PHE4	2.73	-1.77	0	0	HA1	HZ
ARG105	Heavy	HIS6	2.94	-4.06	1	0	HH11	HA
TRP52	Heavy	PHE4	3.23	-2.45	0	0	HH2	CE2
PRO108	Heavy	PHE4	3.51	-1.45	0	0	HD1	HE1
TYR60	Heavy	GLU3	3.54	-1.77	0	-1	O	OE1
ARG106	Heavy	HIS6	4.07	-0.41	1	0	C	HE1
TYR109	Heavy	HIS6	4.15	-1.71	0	0	N	HE2
GLY103	Heavy	HIS6	4.23	-0.75	0	0	HA1	HE1
ASP62	Heavy	GLU3	4.57	1.19	-1	-1	OD1	OE1
GLY101	Heavy	HIS6	4.76	0.01	0	0	HA2	HE1
ILE102	Heavy	PHE4	4.95	-0.29	0	0	O	HZ
ARG106	Heavy	PHE4	5.06	-0.58	1	0	HN	HZ
LYS57	Heavy	PHE4	5.34	-0.21	1	0	HG2	HD2
THR61	Heavy	GLU3	5.36	0.01	0	-1	HA	OE1
GLY103	Heavy	PHE4	5.37	-0.07	0	0	HA1	HZ
ARG105	Heavy	GLU3	5.39	-1.23	1	-1	HH21	O
GLY103	Heavy	ASP7	5.45	-0.27	0	-2	HA1	HN
TRP52	Heavy	HIS6	5.49	-0.15	0	0	HZ2	HE1
VAL50	Heavy	PHE4	5.60	-0.17	0	0	HG12	HB2
TRP47	Heavy	GLU3	5.79	-0.14	0	-1	HH2	OE1
ALA104	Heavy	ASP7	5.80	-0.51	0	-2	HN	HB1
ALA104	Heavy	HIS6	6.09	-0.31	0	0	N	HE1
TRP47	Heavy	PHE4	6.17	-0.10	0	0	HH2	HB2
LYS58	Heavy	GLU3	6.22	-1.16	1	-1	O	OE1
PRO108	Heavy	ARG5	6.35	-0.24	0	1	HD1	HA
ALA104	Heavy	PHE4	6.40	0.09	0	0	HN	HZ
TYR59	Heavy	ALA2	6.42	0.10	0	1	HE1	C
ASP99	Heavy	HIS6	6.44	-0.27	-1	0	OD2	HE2
ASP62	Heavy	ALA2	6.47	-1.95	-1	1	OD2	HA
GLY107	Heavy	ARG5	6.48	-0.18	0	1	HA1	C
TYR59	Heavy	ARG5	6.53	-0.32	0	1	OH	HN
ILE102	Heavy	ASP7	6.56	-0.22	0	-2	HG22	HN
HIS35	Heavy	HIS6	6.66	0.00	0	0	HE1	HE2

LYS65	Heavy	ALA2	6.98	1.81	1	1	HZ1	HA
TRP52	Heavy	ARG5	7.11	-0.05	0	1	HH2	N
TYR110	Heavy	HIS6	7.15	-0.13	0	0	N	HE2
MET111	Heavy	HIS6	7.20	-0.03	0	0	HE3	HE2
VAL50	Heavy	GLU3	7.24	0.06	0	-1	HG13	HB1
TYR109	Heavy	PHE4	7.32	-0.08	0	0	N	HE1
LYS65	Heavy	PHE4	7.33	-0.31	1	0	HZ1	HN
LYS58	Heavy	PHE4	7.33	-0.14	1	0	C	HB1
GLY101	Heavy	PHE4	7.38	-0.03	0	0	HA1	HZ
GLY107	Heavy	ASP7	7.44	0.00	0	-2	HA1	HN
TRP52	Heavy	GLU3	7.44	-0.03	0	-1	HH2	O
TYR60	Heavy	PHE4	7.50	-0.07	0	0	O	HN
ALA49	Heavy	GLU3	7.52	-0.09	0	-1	HA	OE1
ARG105	Heavy	ALA2	7.53	0.96	1	1	HH21	O
VAL50	Heavy	HIS6	7.55	-0.01	0	0	HG23	HE2
LYS57	Heavy	GLU3	7.56	-1.13	1	-1	HB2	HB2
ILE102	Heavy	ARG5	7.58	-0.07	0	1	HG22	C
ARG100	Heavy	HIS6	7.58	0.06	1	0	O	HE1
TRP47	Heavy	ALA2	7.79	-0.14	0	1	HZ3	HA
GLY103	Heavy	ARG5	7.81	-0.01	0	1	HA1	O
THR61	Heavy	ALA2	7.82	0.14	0	1	HA	HA
TYR60	Heavy	ALA2	7.83	-0.27	0	1	O	HA
HIS35	Heavy	PHE4	7.84	-0.04	0	0	HE1	HD1
ASP1	Light	ALA2	1.71	-13.36	0	1	OD1	H3
THR94	Light	PHE4	1.95	-8.74	0	0	OG1	HN
THR94	Light	GLU3	2.00	-7.65	0	-1	HG1	HN
TYR92	Light	HIS6	2.16	-4.95	0	0	O	HN
SER93	Light	ARG5	2.28	-5.12	0	1	HB1	HB2
SER93	Light	ALA2	2.30	0.07	0	1	HB2	HB3
TYR91	Light	HIS6	2.30	-1.52	0	0	O	HD2
SER93	Light	PHE4	2.38	-3.24	0	0	HA	O
TYR92	Light	ARG5	2.50	-13.16	0	1	O	HA
THR94	Light	ALA2	2.53	-0.72	0	1	HN	HB3
TYR32	Light	HIS6	3.36	-2.79	0	0	HH	HB2
SER93	Light	HIS6	3.71	-1.30	0	0	HA	HN
THR94	Light	ARG5	3.98	-1.26	0	1	HN	HA
TYR92	Light	PHE4	4.06	-1.47	0	0	O	HD1
TYR32	Light	ASP7	4.13	-2.23	0	-2	HH	OT2
PRO95	Light	ALA2	4.35	-1.15	0	1	HD1	HA
LEU96	Light	PHE4	4.64	-0.34	0	0	HD21	HD1
TYR92	Light	ASP7	4.71	0.08	0	-2	HE1	HA
ASP1	Light	ARG5	4.88	-0.66	0	1	OD1	HD2
ILE2	Light	ALA2	5.00	-0.46	0	1	HN	HB1
TYR91	Light	PHE4	5.01	-0.30	0	0	O	HE1
PRO95	Light	GLU3	5.15	-1.11	0	-1	N	HN
LEU96	Light	HIS6	5.27	-0.15	0	0	HD22	HD2
SER93	Light	GLU3	5.43	1.40	0	-1	HB2	N
TYR92	Light	ALA2	5.56	-0.07	0	1	HE2	HB1

THR94	Light	HIS6	5.68	-0.16	0	0	HN	HN
TYR91	Light	ARG5	5.69	-0.27	0	1	O	HA
ASP1	Light	GLU3	5.69	0.08	0	-1	OD2	HN
PRO95	Light	PHE4	5.83	-0.27	0	0	N	HN
GLN90	Light	ALA2	6.10	-0.06	0	1	HE22	HB1
SER30	Light	HIS6	6.11	-0.11	0	0	HB1	HB1
SER30	Light	ASP7	6.32	-0.14	0	-2	HB2	OT1
LEU96	Light	ARG5	6.35	-0.05	0	1	HD21	HA
ILE2	Light	ARG5	6.58	-0.07	0	1	HD3	HB2
GLN90	Light	PHE4	6.61	-0.06	0	0	HE22	O
ASP1	Light	PHE4	6.63	-0.12	0	0	HB1	O
GLN90	Light	HIS6	6.65	-0.10	0	0	OE1	HD2
ILE29	Light	HIS6	6.66	-0.09	0	0	O	HB1
LEU96	Light	ALA2	6.76	-0.01	0	1	HD23	HB3
GLN90	Light	ARG5	7.08	-0.16	0	1	OE1	HA
TYR32	Light	ARG5	7.09	-0.22	0	1	HE1	C
ILE29	Light	ARG5	7.32	-0.16	0	1	HG22	HB2
SER31	Light	HIS6	7.34	-0.09	0	0	HN	HB1
TYR32	Light	PHE4	7.37	-0.06	0	0	CE1	HE1
SER28	Light	ARG5	7.39	-0.24	0	1	O	HH11
ILE2	Light	PHE4	7.54	-0.04	0	0	HG12	O
SER93	Light	ASP7	7.64	-0.07	0	-2	HB1	N
THR97	Light	ALA2	7.73	-0.02	0	1	HG21	HB1
ASN34	Light	HIS6	7.76	-0.02	0	0	HD22	HE2

Table S9 – Quantum biochemistry details of FC MD5

Adu Residue	Adu Chain	Aβ Residue	Distance (Å)	Interaction Energy (kcal/mol)	Adu Residue Charge	Aβ Residue Charge	Adu Atom	Aβ Atom
LYS65	Heavy	GLU3	1.71	-15.15	1	-1	HZ1	OE2
PRO108	Heavy	HIS6	1.90	-5.56	0	0	O	HE2
TYR59	Heavy	PHE4	2.09	-2.14	0	0	HE2	HB1
GLY107	Heavy	HIS6	2.64	-1.65	0	0	O	HE1
TYR59	Heavy	GLU3	2.66	-11.26	0	-1	HB2	HB1
TYR60	Heavy	GLU3	2.76	-1.72	0	-1	HN	OE1
ILE102	Heavy	HIS6	2.89	-1.10	0	0	HG23	HE1
GLY107	Heavy	PHE4	3.03	-0.88	0	0	HA1	HZ
PRO108	Heavy	PHE4	3.14	-1.58	0	0	HD1	HE1
TRP52	Heavy	PHE4	3.15	-3.05	0	0	HH2	CD2
TYR109	Heavy	HIS6	3.52	-2.15	0	0	HA	HE2
ASP62	Heavy	GLU3	3.70	2.76	-1	-1	OD2	OE2
THR61	Heavy	GLU3	4.60	-0.40	0	-1	HA	OE1
TRP47	Heavy	GLU3	5.08	-0.15	0	-1	HH2	OE1
VAL50	Heavy	PHE4	5.35	-0.24	0	0	HG12	HB2
ARG106	Heavy	HIS6	5.35	0.16	1	0	C	HE1
GLY101	Heavy	HIS6	5.51	-0.06	0	0	HA2	HE1
TYR59	Heavy	ARG5	5.67	-0.27	0	1	HE2	HN

LYS58	Heavy	GLU3	5.68	-1.20	1	-1	O	HB2
TRP52	Heavy	HIS6	5.81	-0.15	0	0	HZ2	HE1
TRP47	Heavy	PHE4	5.92	-0.13	0	0	HH2	HB2
TRP52	Heavy	ARG5	6.03	-0.12	0	1	HH2	HN
LYS57	Heavy	GLU3	6.04	-1.43	1	-1	HD2	O
LYS65	Heavy	ALA2	6.07	2.12	1	1	HZ1	HA
ILE102	Heavy	PHE4	6.13	-0.09	0	0	HG21	HZ
GLY107	Heavy	ASP7	6.14	-0.10	0	-2	HA1	HN
TRP52	Heavy	GLU3	6.18	-0.05	0	-1	HH2	O
VAL50	Heavy	GLU3	6.26	-0.02	0	-1	HG13	HB1
ARG106	Heavy	PHE4	6.28	0.07	1	0	C	HZ
ASP99	Heavy	HIS6	6.30	-0.28	-1	0	OD2	HE2
ARG105	Heavy	PHE4	6.32	-0.05	1	0	HB1	HE2
LYS57	Heavy	PHE4	6.38	-0.19	1	0	HD2	HB1
TYR110	Heavy	HIS6	6.43	-0.13	0	0	N	HE2
ASP62	Heavy	ALA2	6.45	-2.15	-1	1	OD2	HA
GLY103	Heavy	HIS6	6.51	-0.13	0	0	HA1	HE1
TYR59	Heavy	ALA2	6.65	-0.21	0	1	HB2	HB1
HIS35	Heavy	HIS6	6.71	-0.01	0	0	HE1	HE2
GLY107	Heavy	ARG5	6.85	-0.11	0	1	HA1	O
HIS35	Heavy	PHE4	6.88	-0.04	0	0	HE1	HD1
ILE102	Heavy	ASP7	6.89	-0.06	0	-2	HG21	HN
TYR109	Heavy	PHE4	6.93	-0.11	0	0	HA	HE1
LYS65	Heavy	PHE4	6.96	-0.21	1	0	HZ1	HN
ALA49	Heavy	GLU3	6.96	-0.14	0	-1	HA	OE1
LYS58	Heavy	PHE4	7.10	-0.15	1	0	C	HB1
PRO108	Heavy	ARG5	7.16	-0.38	0	1	HD1	C
ARG105	Heavy	HIS6	7.19	-0.08	1	0	O	HE1
VAL64	Heavy	GLU3	7.25	-0.21	0	-1	HG23	OE1
TRP47	Heavy	ALA2	7.26	-0.09	0	1	HH2	HB1
ARG105	Heavy	ASP7	7.33	-1.79	1	-2	HB1	OD1
TYR60	Heavy	PHE4	7.36	-0.07	0	0	HN	HN
TRP52	Heavy	ASP7	7.40	-0.11	0	-2	HZ2	OD1
VAL48	Heavy	GLU3	7.45	0.11	0	-1	O	OE1
GLY103	Heavy	ASP7	7.78	-0.10	0	-2	HA1	HB1
GLY101	Heavy	PHE4	7.82	-0.01	0	0	HA1	HZ
THR61	Heavy	ALA2	7.85	0.22	0	1	HA	HB1
PRO108	Heavy	ASP7	7.88	0.35	0	-2	HD1	HN
VAL50	Heavy	HIS6	7.90	-0.01	0	0	HG21	HE2
SER93	Light	PHE4	1.80	-10.50	0	0	HG1	O
TYR92	Light	HIS6	2.22	-6.29	0	0	O	HN
THR94	Light	PHE4	2.23	-4.55	0	0	OG1	HN
THR94	Light	ALA2	2.24	-1.60	0	1	HG1	HB1
THR94	Light	GLU3	2.33	-5.96	0	-1	HG1	HN
SER93	Light	ALA2	2.41	-0.79	0	1	HG1	HB3
TYR91	Light	HIS6	2.47	-1.37	0	0	O	HD2
SER93	Light	ARG5	2.55	-7.87	0	1	HB2	HA
TYR32	Light	HIS6	2.80	-4.18	0	0	HD2	HD2

TYR92	Light	ARG5	3.02	-11.86	0	1	O	HA
SER93	Light	HIS6	3.63	-1.61	0	0	HA	HN
LEU96	Light	PHE4	3.75	-0.48	0	0	HD21	HD1
THR94	Light	ARG5	3.95	-1.00	0	1	HN	HA
TYR92	Light	PHE4	4.36	-0.93	0	0	O	HD1
PRO95	Light	ALA2	4.71	-0.53	0	1	N	HB1
TYR91	Light	PHE4	4.77	-0.32	0	0	O	HE1
SER93	Light	GLU3	4.91	2.13	0	-1	HG1	HN
TYR32	Light	ASP7	5.03	-0.69	0	-2	HH	OT1
LEU96	Light	HIS6	5.20	-0.17	0	0	HD21	HD2
TYR92	Light	ASP7	5.29	0.46	0	-2	HE2	OT2
PRO95	Light	GLU3	5.33	-0.93	0	-1	HD2	HN
ASP1	Light	ALA2	5.61	-0.02	0	1	HA	HB2
ILE2	Light	ARG5	5.67	-0.07	0	1	HD1	HG1
THR94	Light	HIS6	5.69	-0.15	0	0	HN	HN
TYR32	Light	PHE4	5.77	-0.15	0	0	HE2	HE1
ILE2	Light	ALA2	6.04	-0.11	0	1	HG11	HB2
PRO95	Light	PHE4	6.14	-0.28	0	0	N	HN
LEU96	Light	ARG5	6.27	-0.07	0	1	HD21	HA
TYR91	Light	ARG5	6.37	0.07	0	1	O	HA
GLN27	Light	ARG5	6.39	-0.31	0	1	OE1	HD2
ASN34	Light	HIS6	6.50	-0.04	0	0	HD22	HE2
SER30	Light	HIS6	6.51	-0.10	0	0	HB1	HB2
ILE29	Light	HIS6	6.69	-0.10	0	0	O	HB1
TYR92	Light	ALA2	6.70	-0.09	0	1	O	HB3
ILE2	Light	PHE4	6.74	-0.05	0	0	HD1	O
ILE2	Light	HIS6	6.78	-0.04	0	0	HD1	HN
GLN90	Light	ALA2	6.78	-0.02	0	1	HE22	HB2
GLN90	Light	HIS6	6.83	-0.12	0	0	C	HD2
GLN90	Light	PHE4	6.84	-0.07	0	0	HE22	O
GLN27	Light	ALA2	7.05	-0.04	0	1	HE22	HB2
GLN90	Light	ARG5	7.08	-0.11	0	1	HE22	HA
SER31	Light	HIS6	7.16	-0.16	0	0	HG1	HD2
SER30	Light	ASP7	7.20	-0.30	0	-2	HG1	OT2
ILE29	Light	ARG5	7.37	-0.14	0	1	HA	HG1
LEU96	Light	ALA2	7.39	-0.08	0	1	HD23	HB1
TYR32	Light	ARG5	7.58	-0.33	0	1	OH	C
SER93	Light	ASP7	7.65	-0.06	0	-2	HB2	N
SER28	Light	ARG5	7.74	-0.18	0	1	O	HD2
LEU33	Light	HIS6	7.80	-0.05	0	0	N	HD2

Table S10 – Quantum biochemistry details of FC MD6

**Adu Residue**	**Adu Chain**	**Aβ Residue**	**Distance (Å)**	**Interaction Energy (kcal/mol)**	**Adu Residue Charge**	**Aβ Residue Charge**	**Adu Atom**	**Aβ Atom**
LYS57	Heavy	GLU3	1.77	-13.88	1	-1	HZ2	OE1
TYR59	Heavy	GLU3	1.81	-10.78	0	-1	HH	OE1

TYR59	Heavy	PHE4	1.90	-3.48	0	0	HE2	HB1
ILE102	Heavy	HIS6	1.91	-0.08	0	0	HG21	HE1
PRO108	Heavy	HIS6	1.93	-6.15	0	0	O	HE2
GLY107	Heavy	HIS6	2.32	-3.29	0	0	O	HE1
LYS57	Heavy	PHE4	2.64	-5.82	1	0	HG1	CD2
TRP52	Heavy	PHE4	2.67	-6.44	0	0	CE2	HD2
ALA104	Heavy	ASP7	3.02	-4.26	0	-2	HB3	OD1
ARG105	Heavy	PHE4	3.16	-6.19	1	0	HD2	CE1
LYS57	Heavy	ALA2	3.52	1.90	1	1	HZ1	H3
THR56	Heavy	PHE4	3.56	-0.81	0	0	OG1	HE2
TYR109	Heavy	HIS6	3.77	-1.59	0	0	HA	HE2
ASP54	Heavy	PHE4	3.88	-0.61	-1	0	OD1	HE2
TRP52	Heavy	ARG5	3.95	-0.61	0	1	HH2	HA
ARG105	Heavy	ARG5	4.17	0.01	1	1	HG1	HN
ARG105	Heavy	ASP7	4.33	-4.60	1	-2	HN	OD1
GLY103	Heavy	HIS6	4.39	-0.27	0	0	HA1	HE1
ARG106	Heavy	HIS6	4.53	-1.74	1	0	C	HE1
GLY101	Heavy	HIS6	4.62	0.47	0	0	HA2	HE1
TYR59	Heavy	ALA2	4.78	-0.39	0	1	OH	O
ARG105	Heavy	ALA2	4.90	1.14	1	1	HH12	HB2
TYR59	Heavy	ARG5	4.94	-0.19	0	1	HE2	N
GLY103	Heavy	ASP7	5.13	-2.08	0	-2	HA1	HB1
GLY107	Heavy	PHE4	5.16	-0.16	0	0	HA1	HB2
TRP52	Heavy	HIS6	5.23	-0.56	0	0	HH2	N
ARG105	Heavy	HIS6	5.31	-1.17	1	0	HB1	HA
GLY107	Heavy	ARG5	5.36	-0.40	0	1	HA1	HA
PHE53	Heavy	PHE4	5.65	-0.32	0	0	HN	HD2
PRO108	Heavy	ARG5	5.66	-0.11	0	1	HD1	HA
LYS58	Heavy	GLU3	5.75	-1.06	1	-1	HN	OE1
ARG105	Heavy	GLU3	5.94	-1.60	1	-1	HG1	O
ILE102	Heavy	ASP7	5.96	-0.13	0	-2	HG21	HN
ALA104	Heavy	HIS6	5.97	-0.19	0	0	HN	HA
PRO108	Heavy	PHE4	6.27	-0.13	0	0	HD1	HB2
ALA104	Heavy	ARG5	6.34	-0.04	0	1	HB3	O
ASP99	Heavy	HIS6	6.49	-0.27	-1	0	OD2	HE2
ARG106	Heavy	ASP7	6.50	-2.39	1	-2	HN	OD1
ARG106	Heavy	PHE4	6.53	-0.23	1	0	N	HD2
GLY107	Heavy	ASP7	6.62	-0.20	0	-2	HN	HN
TRP52	Heavy	GLU3	6.75	-0.27	0	-1	HZ3	HB2
TYR110	Heavy	HIS6	6.86	-0.09	0	0	N	HE2
LYS65	Heavy	GLU3	6.90	-1.22	1	-1	HZ2	HG1
LYS58	Heavy	PHE4	6.93	-0.24	1	0	HN	HN
VAL50	Heavy	PHE4	7.05	-0.04	0	0	HG11	HB1
GLY55	Heavy	PHE4	7.05	-0.14	0	0	HN	HE2
LYS57	Heavy	ARG5	7.12	0.58	1	1	HE1	HN
ARG106	Heavy	ARG5	7.37	0.27	1	1	HN	O
GLY103	Heavy	ARG5	7.42	0.00	0	1	HA1	O
HIS35	Heavy	HIS6	7.43	0.00	0	0	HE1	HE2

TYR60	Heavy	GLU3	7.45	-0.03	0	-1	HN	HG1
TYR59	Heavy	HIS6	7.64	-0.05	0	0	HE2	N
ILE51	Heavy	PHE4	7.67	-0.05	0	0	C	HD2
ALA104	Heavy	PHE4	7.68	-0.19	0	0	HB3	HB2
ILE102	Heavy	ARG5	7.71	-0.09	0	1	O	O
ARG100	Heavy	HIS6	7.77	0.05	1	0	C	HE1
TRP52	Heavy	ASP7	7.88	-0.18	0	-2	HZ2	OD1
TYR32	Light	ASP7	1.88	-11.39	0	-2	HH	OT1
TYR92	Light	HIS6	1.92	-7.57	0	0	O	HN
TYR32	Light	HIS6	2.18	-7.36	0	0	HB2	HD2
SER93	Light	ARG5	2.20	-6.42	0	1	HB2	HB1
TYR91	Light	HIS6	2.26	1.07	0	0	HB1	HD2
TYR92	Light	ARG5	2.62	-14.50	0	1	O	HB1
TYR92	Light	ASP7	3.59	-2.46	0	-2	HE1	OT2
SER93	Light	HIS6	3.67	-2.03	0	0	HA	HN
THR94	Light	PHE4	3.75	-1.86	0	0	HG1	O
THR94	Light	ARG5	3.87	-1.45	0	1	HN	HB1
SER30	Light	ASP7	4.12	-2.00	0	-2	HG1	OT1
SER93	Light	PHE4	4.65	-1.72	0	0	HA	O
TYR91	Light	ARG5	4.87	-1.17	0	1	O	HA
THR94	Light	GLU3	4.92	-0.08	0	-1	HG1	HB2
LEU96	Light	HIS6	5.20	-0.16	0	0	HD22	HD2
GLN90	Light	HIS6	5.32	-0.10	0	0	C	HD2
ILE2	Light	ARG5	5.45	-0.10	0	1	HD3	HD2
THR94	Light	HIS6	5.49	-0.18	0	0	HN	HN
SER30	Light	HIS6	5.56	-0.17	0	0	HB1	HB2
ILE29	Light	HIS6	5.68	-0.21	0	0	O	HB1
TYR92	Light	PHE4	5.70	-0.44	0	0	O	O
LEU33	Light	HIS6	5.91	-0.24	0	0	N	HD2
LEU96	Light	ARG5	5.96	-0.05	0	1	HD21	HA
SER31	Light	ASP7	6.21	-0.69	0	-2	HN	OT1
SER31	Light	HIS6	6.44	-0.08	0	0	HG1	HB2
LEU96	Light	PHE4	6.68	-0.11	0	0	HD21	O
SER93	Light	GLU3	6.74	-0.08	0	-1	OG	HB2
GLN27	Light	ARG5	6.75	-0.07	0	1	HE22	HH21
ASN34	Light	HIS6	6.78	-0.09	0	0	HD22	HE2
ILE29	Light	ASP7	6.87	0.08	0	-2	O	OT1
TYR91	Light	PHE4	6.89	-0.06	0	0	O	O
TYR32	Light	ARG5	7.05	-0.32	0	1	HH	O
GLN90	Light	ARG5	7.37	-0.10	0	1	OE1	HB1
TYR91	Light	ASP7	7.40	0.08	0	-2	O	N
ILE2	Light	HIS6	7.50	-0.04	0	0	HD3	HN
ILE29	Light	ARG5	7.62	-0.17	0	1	HG22	HD2
SER93	Light	ASP7	7.65	0.06	0	-2	HB2	N
ALA50	Light	HIS6	7.88	0.00	0	0	HA	HE2
GLN89	Light	HIS6	7.92	-0.05	0	0	O	HD2
PRO95	Light	ARG5	7.99	-0.25	0	1	N	HB1

Table S11 – Quantum biochemistry details of FC LMD-Mut

Adu Residue	Adu Chain	Aβ Residue	Distance (Å)	Interaction Energy (kcal/mol)	Adu Residue Charge	Aβ Residue Charge	Adu Atom	Aβ Atom
LYS65	Heavy	GLU3	1.58	-13.71	1	-1	HZ2	OE2
PRO108	Heavy	HIS6	2.22	-5.12	0	0	O	HE2
TYR59	Heavy	GLU3	2.23	-4.87	0	-1	HD2	HB2
PRO108	Heavy	PHE4	2.26	-2.19	0	0	HD1	HZ
TRP52	Heavy	PHE4	2.82	-2.16	0	0	HH2	HZ
GLY107	Heavy	PHE4	3.07	-0.30	0	0	HA1	HZ
TYR59	Heavy	PHE4	3.23	-4.15	0	0	CE2	HB1
ILE102	Heavy	HIS6	3.29	-0.96	0	0	HG21	HE1
GLY107	Heavy	HIS6	3.57	-0.91	0	0	HA1	HE1
TYR60	Heavy	GLU3	3.84	-0.52	0	-1	HN	OE2
TYR109	Heavy	HIS6	4.01	-1.38	0	0	HA	HE2
ASP62	Heavy	GLU3	4.39	1.38	-1	-1	OD1	OE2
VAL50	Heavy	PHE4	4.80	-0.32	0	0	HG11	HD1
TRP47	Heavy	GLU3	5.34	-0.29	0	-1	HH2	OE2
GLY103	Heavy	HIS6	5.35	-0.19	0	0	HA1	HE1
TRP52	Heavy	HIS6	5.44	-0.16	0	0	HH2	HE1
TRP47	Heavy	PHE4	5.45	-0.18	0	0	HH2	HB2
THR61	Heavy	GLU3	5.70	0.17	0	-1	HA	OE2
GLY101	Heavy	HIS6	5.86	0.00	0	0	HA2	HE2
LYS57	Heavy	PHE4	5.86	-0.27	1	0	HE2	HB1
ARG106	Heavy	HIS6	5.87	0.04	1	0	C	HE1
LYS58	Heavy	GLU3	5.89	-1.08	1	-1	O	HB2
LYS57	Heavy	GLU3	5.99	-1.95	1	-1	HE2	HB2
ILE102	Heavy	PHE4	6.12	-0.07	0	0	HG21	HZ
ARG106	Heavy	PHE4	6.30	0.13	1	0	C	HZ
TYR59	Heavy	ALA2	6.33	0.03	0	1	HE2	O
HIS35	Heavy	PHE4	6.43	-0.04	0	0	HE1	HE1
ARG105	Heavy	PHE4	6.60	-0.04	1	0	HB1	HE2
LYS65	Heavy	ALA2	6.60	1.89	1	1	HZ2	HB3
ASP99	Heavy	HIS6	6.66	-0.21	-1	0	OD1	HE2
ASP62	Heavy	ALA2	6.67	-1.62	-1	1	OD2	HA
TYR109	Heavy	PHE4	6.68	-0.09	0	0	N	HE1
TYR59	Heavy	ARG5	6.71	-0.35	0	1	OH	HN
TYR110	Heavy	HIS6	6.80	-0.10	0	0	N	HE2
LYS65	Heavy	PHE4	6.82	-0.28	1	0	HZ2	HN
PRO108	Heavy	ARG5	6.84	-0.18	0	1	HG2	HA
LYS58	Heavy	PHE4	6.92	-0.16	1	0	O	HB1
TRP52	Heavy	ARG5	7.02	-0.13	0	1	HH2	HN
VAL50	Heavy	GLU3	7.06	0.01	0	-1	HG12	HB1
TRP47	Heavy	ALA2	7.09	-0.09	0	1	HZ3	HB3
GLY101	Heavy	PHE4	7.29	-0.01	0	0	HA2	HZ
ARG105	Heavy	HIS6	7.44	-0.06	1	0	C	HE1
ASP99	Heavy	PHE4	7.47	-0.02	-1	0	OD2	HE1

TYR60	Heavy	PHE4	7.61	-0.07	0	0	O	HB2
GLY103	Heavy	PHE4	7.64	-0.01	0	0	HA1	HZ
GLY107	Heavy	ARG5	7.70	0.04	0	1	HA1	O
TRP52	Heavy	GLU3	7.70	0.00	0	-1	HZ3	O
HIS35	Heavy	HIS6	7.82	0.00	0	0	HE1	HE2
TYR60	Heavy	ALA2	7.85	-0.24	0	1	O	HB3
ALA104	Heavy	HIS6	7.89	-0.02	0	0	HN	HE1
ALA49	Heavy	GLU3	7.91	-0.14	0	-1	HA	OE2
THR61	Heavy	ALA2	7.96	-0.05	0	1	HA	HB3
TYR92	Light	ASP7	1.78	-8.72	0	-2	HH	OD2
THR94	Light	PHE4	1.80	-4.78	0	0	HN	O
TYR92	Light	HIS6	1.87	-8.34	0	0	O	HN
SER93	Light	ARG5	2.16	-3.89	0	1	HA	HA
TYR91	Light	HIS6	2.31	-1.62	0	0	O	HD2
TYR92	Light	ARG5	2.40	-9.59	0	1	O	HA
SER93	Light	PHE4	2.71	-5.45	0	0	HA	O
THR94	Light	ALA2	2.87	-1.93	0	1	HG1	HB3
THR94	Light	GLU3	2.88	-2.05	0	-1	HG1	HB1
TYR32	Light	HIS6	2.95	-5.52	0	0	OH	HB2
SER93	Light	ALA2	3.11	-0.98	0	1	OG	HB2
LEU96	Light	PHE4	3.26	-0.75	0	0	HD22	HD1
THR94	Light	ARG5	3.29	-2.22	0	1	HN	HA
SER93	Light	HIS6	3.69	-1.43	0	0	HA	HN
PRO95	Light	ALA2	4.21	-0.27	0	1	HD2	HB3
TYR92	Light	PHE4	4.48	-2.23	0	0	O	O
TYR91	Light	PHE4	4.76	-0.25	0	0	O	HE1
TYR32	Light	ASP7	4.80	-0.40	0	-2	HH	HA
LEU96	Light	HIS6	5.19	-0.12	0	0	HD23	HD2
TYR91	Light	ARG5	5.30	-0.13	0	1	O	HA
LEU96	Light	ARG5	5.39	-0.11	0	1	HD22	HA
THR94	Light	HIS6	5.48	-0.18	0	0	HN	HN
PRO95	Light	GLU3	5.54	-0.29	0	-1	HD2	HN
SER93	Light	GLU3	5.86	-0.01	0	-1	HG1	HN
SER30	Light	HIS6	5.95	-0.17	0	0	HB1	HB1
PRO95	Light	PHE4	5.96	-0.29	0	0	N	O
ASP1	Light	ALA2	6.26	0.06	0	1	HA	HB1
ILE2	Light	ARG5	6.33	-0.03	0	1	HD1	HB1
ILE29	Light	HIS6	6.46	-0.11	0	0	O	HB1
SER93	Light	ASP7	6.51	0.01	0	-2	HB2	OD2
ILE2	Light	ALA2	6.87	-0.05	0	1	HD1	HB1
GLN90	Light	ARG5	6.92	-0.10	0	1	OE1	HB1
GLN90	Light	PHE4	6.96	-0.06	0	0	HE22	O
TYR32	Light	ARG5	6.97	-0.44	0	1	HE1	HB1
TYR92	Light	ALA2	7.00	-0.18	0	1	O	HB2
GLN90	Light	HIS6	7.00	-0.11	0	0	C	HD2
ILE29	Light	ARG5	7.04	-0.06	0	1	HA	HB1
ASN34	Light	HIS6	7.08	-0.01	0	0	HD22	HE2
SER30	Light	ASP7	7.32	-0.19	0	-2	HB2	HA

TYR32	Light	PHE4	7.33	-0.07	0	0	HB2	HE1
SER31	Light	HIS6	7.37	-0.33	0	0	HN	HB1
PRO95	Light	ARG5	7.64	-0.18	0	1	N	HB2
GLN90	Light	ALA2	7.66	-0.02	0	1	HE22	HB3
SER28	Light	ARG5	7.72	-0.12	0	1	O	HB1
ILE2	Light	PHE4	7.73	-0.03	0	0	HD1	O
LEU33	Light	HIS6	7.75	-0.05	0	0	N	HD2
LEU96	Light	GLU3	7.85	-0.05	0	-1	HD22	HB1
LEU96	Light	ALA2	7.87	-0.06	0	1	N	HB3

Table S12 – Quantum biochemistry details of RC MD2-#0

Adu Residue	Adu Chain	Aβ Residue	Distance (Å)	Interaction Energy (kcal/mol)	Adu Residue Charge	Aβ Residue Charge	Adu Atom	Aβ Atom
LYS65	Heavy	GLU3	1.69	-10.56	1	-1	HZ3	OE1
PRO108	Heavy	HIS6	1.70	-3.27	0	0	O	HE2
GLY107	Heavy	HIS6	2.25	-1.25	0	0	HA3	HE1
TYR59	Heavy	GLU3	2.25	-2.93	0	-1	HE1	HB2
ILE102	Heavy	HIS6	2.47	-1.70	0	0	HG21	HE1
ARG105	Heavy	ASP7	2.61	-4.49	1	-2	HH21	HB3
TYR59	Heavy	PHE4	2.71	-4.39	0	0	HH	HD2
GLY107	Heavy	PHE4	2.72	-1.27	0	0	HA3	HE1
ARG105	Heavy	PHE4	2.76	-1.64	1	0	HB3	HE2
TYR109	Heavy	HIS6	3.06	-2.47	0	0	HA	HE2
TRP52	Heavy	PHE4	3.16	-3.28	0	0	HH2	CD1
PRO108	Heavy	PHE4	3.26	-1.50	0	0	HD3	HD1
TYR60	Heavy	GLU3	4.25	0.12	0	-1	H	OE1
ASP62	Heavy	GLU3	4.27	1.93	-1	-1	OD1	OE1
ARG106	Heavy	HIS6	4.37	-0.07	1	0	C	HE1
LYS57	Heavy	PHE4	4.72	-0.29	1	0	HE2	HD2
ARG106	Heavy	PHE4	4.75	0.01	1	0	N	HZ
GLY101	Heavy	HIS6	4.85	-0.23	0	0	HA2	HE1
GLY103	Heavy	HIS6	5.04	-0.06	0	0	HA3	HE1
ILE102	Heavy	PHE4	5.19	-0.19	0	0	O	HZ
ARG105	Heavy	HIS6	5.37	-0.43	1	0	HD3	HA
GLY103	Heavy	ASP7	5.50	-0.33	0	-2	HA3	OXT
TRP52	Heavy	HIS6	5.58	-0.14	0	0	HZ2	HE1
ARG105	Heavy	ARG5	5.66	0.85	1	1	HD3	O
VAL50	Heavy	PHE4	5.73	-0.15	0	0	HG11	HD1
ALA104	Heavy	ASP7	5.80	-0.50	0	-2	H	OXT
TYR59	Heavy	ARG5	5.92	-0.26	0	1	HH	H
ILE102	Heavy	ASP7	6.02	-0.07	0	-2	HG22	H
THR61	Heavy	GLU3	6.03	0.16	0	-1	HA	OE1
GLY103	Heavy	PHE4	6.11	-0.05	0	0	HA3	HZ
TYR110	Heavy	HIS6	6.14	-0.18	0	0	N	HE2
TRP47	Heavy	PHE4	6.15	-0.10	0	0	HH2	HB2
TRP52	Heavy	ARG5	6.18	-0.13	0	1	HH2	H

ASP99	Heavy	HIS6	6.25	-0.23	-1	0	OD2	HE2
TRP47	Heavy	GLU3	6.26	-0.01	0	-1	HH2	HB3
TYR59	Heavy	ALA2	6.27	0.11	0	1	HE1	C
LYS57	Heavy	GLU3	6.37	-1.49	1	-1	HZ2	HB2
LYS58	Heavy	GLU3	6.38	-0.86	1	-1	O	OE2
GLY107	Heavy	ARG5	6.38	-0.04	0	1	HA3	C
ALA104	Heavy	PHE4	6.66	0.06	0	0	C	HZ
PRO108	Heavy	ARG5	6.69	-0.29	0	1	HD3	HA
ASP62	Heavy	ALA2	6.75	-1.62	-1	1	OD1	HA
HIS35	Heavy	PHE4	6.81	-0.03	0	0	HE1	HD1
HIS35	Heavy	HIS6	6.83	-0.01	0	0	HE1	HE2
GLY107	Heavy	ASP7	6.88	-0.13	0	-2	H	H
ALA104	Heavy	HIS6	6.95	-0.09	0	0	N	HE1
LYS65	Heavy	ALA2	6.95	1.59	1	1	HZ3	HA
LYS58	Heavy	PHE4	6.98	-0.11	1	0	O	HB3
TYR109	Heavy	PHE4	7.07	-0.10	0	0	HA	HE1
TRP52	Heavy	GLU3	7.20	-0.03	0	-1	HH2	O
LYS65	Heavy	PHE4	7.31	-0.18	1	0	HZ3	H
ARG106	Heavy	ASP7	7.38	-1.34	1	-2	H	H
TYR60	Heavy	PHE4	7.42	-0.08	0	0	H	H
GLY101	Heavy	PHE4	7.46	-0.01	0	0	HA2	HE1
THR61	Heavy	ALA2	7.86	-0.03	0	1	HA	HA
VAL50	Heavy	GLU3	7.90	0.03	0	-1	HG12	HB3
TYR60	Heavy	ALA2	7.93	-0.22	0	1	O	HA
ARG100	Heavy	HIS6	7.93	-0.02	1	0	C	HE1
TRP47	Heavy	ALA2	7.96	-0.16	0	1	HZ3	HA
ARG105	Heavy	GLU3	7.99	-0.67	1	-1	HG2	O
THR94	Light	PHE4	2.04	-3.29	0	0	H	O
TYR92	Light	HIS6	2.06	-4.86	0	0	O	H
ASP1	Light	ALA2	2.06	-3.88	0	1	OD1	H2
SER93	Light	ALA2	2.11	-1.14	0	1	HB2	HB1
SER93	Light	ARG5	2.17	-4.24	0	1	HA	HA
SER93	Light	PHE4	2.43	-3.16	0	0	HB2	O
TYR92	Light	ARG5	2.44	-6.73	0	1	O	HA
TYR32	Light	HIS6	2.48	-5.19	0	0	HH	HB2
SER91	Light	HIS6	2.53	-0.23	0	0	O	HD2
THR94	Light	GLU3	2.64	-1.17	0	-1	HG1	HB3
THR94	Light	ALA2	2.70	-1.85	0	1	O	HB1
SER93	Light	HIS6	3.70	-1.48	0	0	HA	H
THR94	Light	ARG5	3.83	-1.59	0	1	H	HA
PRO95	Light	ALA2	4.29	-0.53	0	1	HD2	HA
LEU96	Light	PHE4	4.34	-0.32	0	0	HD22	HD1
TYR92	Light	PHE4	4.45	-2.00	0	0	O	O
TYR32	Light	ASP7	4.48	-0.49	0	-2	HH	H
TYR92	Light	ASP7	4.70	0.23	0	-2	HE1	HA
ILE2	Light	ALA2	4.82	-0.46	0	1	H	HB2
PRO95	Light	GLU3	5.07	-0.33	0	-1	HD2	H
ASP1	Light	ARG5	5.11	-0.74	0	1	OD2	HB2

SER91	Light	PHE4	5.15	-0.22	0	0	O	HE1
SER93	Light	GLU3	5.30	0.15	0	-1	HB2	N
SER91	Light	ARG5	5.54	-0.03	0	1	O	HA
TYR92	Light	ALA2	5.69	-0.15	0	1	HE2	HB3
THR94	Light	HIS6	5.76	-0.10	0	0	H	H
ASP1	Light	GLU3	5.79	-0.06	0	-1	H3	H
ILE2	Light	ARG5	5.80	-0.11	0	1	HD12	HB3
PRO95	Light	PHE4	5.82	-0.09	0	0	N	H
LEU96	Light	HIS6	6.03	-0.12	0	0	HD22	HE2
LEU96	Light	ARG5	6.13	-0.07	0	1	HD22	HA
LEU96	Light	ALA2	6.22	-0.06	0	1	HD21	HB1
GLN90	Light	ALA2	6.23	-0.05	0	1	HE22	HB2
SER30	Light	HIS6	6.42	-0.08	0	0	HB3	HB3
ILE29	Light	HIS6	6.49	-0.13	0	0	O	HD2
TYR32	Light	ARG5	6.49	-0.18	0	1	HE1	C
ASN34	Light	HIS6	6.50	-0.02	0	0	HD22	HE2
ASP1	Light	PHE4	6.60	-0.09	0	0	OD2	O
GLN90	Light	HIS6	6.66	-0.10	0	0	OE1	HD2
GLN90	Light	PHE4	6.80	-0.06	0	0	HE22	O
GLN90	Light	ARG5	6.94	-0.09	0	1	OE1	HA
ILE2	Light	PHE4	7.03	-0.04	0	0	HG12	O
ILE29	Light	ARG5	7.20	-0.04	0	1	HG23	HB3
TYR32	Light	PHE4	7.25	-0.08	0	0	OH	HE1
ILE2	Light	HIS6	7.27	-0.07	0	0	HD12	H
SER31	Light	HIS6	7.33	-0.10	0	0	C	HD2
SER30	Light	ASP7	7.43	-0.13	0	-2	HB2	O
GLN27	Light	ALA2	7.46	-0.16	0	1	OE1	HB2
LEU33	Light	HIS6	7.58	-0.05	0	0	N	HD2
THR97	Light	ALA2	7.95	-0.05	0	1	H	HB2
SER93	Light	ASP7	7.97	-0.04	0	-2	HB3	N

Table S13 – Quantum biochemistry details of RC MD2-#1

**Adu Residue**	**Adu Chain**	**Aβ Residue**	**Distance (Å)**	**Interaction Energy (kcal/mol)**	**Adu Residue Charge**	**Aβ Residue Charge**	**Adu Atom**	**Aβ Atom**
LYS65	Heavy	GLU3	1.61	-14.19	1	-1	HZ3	OE1
PRO108	Heavy	HIS6	2.06	-3.17	0	0	O	HE2
ILE102	Heavy	HIS6	2.31	-1.05	0	0	HG22	HE1
GLY107	Heavy	PHE4	2.33	-1.13	0	0	HA3	HE1
GLY107	Heavy	HIS6	2.37	-1.57	0	0	O	HE1
TYR59	Heavy	GLU3	2.39	-2.89	0	-1	HD1	HB2
TYR109	Heavy	HIS6	2.49	-2.23	0	0	HA	HE2
PRO108	Heavy	PHE4	2.69	-2.00	0	0	HD3	HE1
TRP52	Heavy	PHE4	2.92	-2.85	0	0	HH2	CE2
TYR59	Heavy	PHE4	3.04	-4.81	0	0	CZ	HB3
ARG105	Heavy	PHE4	3.28	-1.17	1	0	HB3	HZ
ARG105	Heavy	ASP7	3.61	-2.83	1	-2	HB3	OD1

TYR60	Heavy	GLU3	3.90	0.38	0	-1	O	OE1
GLY101	Heavy	HIS6	4.00	0.06	0	0	HA2	HE1
ASP62	Heavy	GLU3	4.21	2.33	-1	-1	OD2	OE1
ARG106	Heavy	PHE4	4.35	-0.32	1	0	C	HZ
GLY103	Heavy	ASP7	4.69	-0.51	0	-2	HA3	HB3
ARG106	Heavy	HIS6	4.72	0.03	1	0	C	HE1
ALA104	Heavy	ASP7	4.94	-0.45	0	-2	HB1	OD1
ILE102	Heavy	ASP7	5.23	-0.11	0	-2	HG23	H
LYS57	Heavy	PHE4	5.28	-0.26	1	0	HB2	HD2
ILE102	Heavy	PHE4	5.40	-0.14	0	0	O	HZ
THR61	Heavy	GLU3	5.48	0.22	0	-1	HA	OE1
GLY103	Heavy	HIS6	5.54	0.00	0	0	N	HE1
ARG105	Heavy	HIS6	5.62	-0.23	1	0	HB3	HA
VAL50	Heavy	PHE4	5.63	-0.21	0	0	HG12	HB2
LYS58	Heavy	GLU3	5.63	-0.92	1	-1	O	HB2
TYR110	Heavy	HIS6	5.65	-0.30	0	0	N	HE2
GLY107	Heavy	ARG5	5.81	-0.17	0	1	HA3	O
TRP47	Heavy	PHE4	5.84	-0.12	0	0	HH2	HB2
TRP47	Heavy	GLU3	5.85	-0.05	0	-1	HH2	HB3
GLY107	Heavy	ASP7	6.00	-0.06	0	-2	H	OD1
LYS58	Heavy	PHE4	6.03	-0.14	1	0	O	HB3
ARG106	Heavy	ASP7	6.04	-1.84	1	-2	H	OD1
ARG105	Heavy	ARG5	6.15	0.64	1	1	HB3	O
PRO108	Heavy	ARG5	6.24	-0.38	0	1	HD3	HA
TYR109	Heavy	PHE4	6.27	-0.12	0	0	HB3	HE1
ASP99	Heavy	HIS6	6.31	-0.26	-1	0	OD2	HE2
GLY103	Heavy	PHE4	6.51	-0.03	0	0	HA3	HZ
TYR59	Heavy	ALA2	6.51	-0.07	0	1	HE1	C
TRP52	Heavy	ARG5	6.53	-0.08	0	1	HH2	H
TRP52	Heavy	HIS6	6.58	-0.10	0	0	HH2	HE1
LYS57	Heavy	GLU3	6.68	-1.29	1	-1	HE2	O
TYR59	Heavy	ARG5	6.69	-0.20	0	1	CZ	H
GLY101	Heavy	PHE4	6.77	-0.03	0	0	HA3	HZ
LYS65	Heavy	ALA2	6.83	1.58	1	1	HZ3	HA
TYR60	Heavy	PHE4	6.99	-0.09	0	0	H	HB3
ARG100	Heavy	HIS6	7.08	-0.01	1	0	C	HE1
LYS65	Heavy	PHE4	7.09	-0.17	1	0	HZ3	H
TRP52	Heavy	ASP7	7.13	-0.08	0	-2	HZ2	OD1
ALA104	Heavy	PHE4	7.14	0.04	0	0	C	HZ
TRP52	Heavy	GLU3	7.16	-0.03	0	-1	HH2	O
ARG105	Heavy	GLU3	7.21	-0.81	1	-1	HH11	O
ASP62	Heavy	ALA2	7.36	-1.37	-1	1	OD2	HA
VAL50	Heavy	GLU3	7.36	0.03	0	-1	HG13	HB3
HIS35	Heavy	HIS6	7.39	0.00	0	0	HE1	HE2
HIS35	Heavy	PHE4	7.65	-0.03	0	0	HE1	HD1
TRP47	Heavy	ALA2	7.82	-0.13	0	1	HH2	HB1
ALA104	Heavy	HIS6	7.84	-0.03	0	0	H	ND1
ASP1	Light	ALA2	1.62	-13.33	0	1	OD2	H3

THR94	Light	PHE4	2.09	-4.84	0	0	HG21	HB2
THR94	Light	ALA2	2.13	-0.54	0	1	H	HB1
TYR92	Light	HIS6	2.14	-5.79	0	0	O	H
THR94	Light	GLU3	2.27	-1.67	0	-1	HG1	HB3
TYR92	Light	ARG5	2.30	-8.41	0	1	OH	HD2
SER93	Light	ALA2	2.32	-1.34	0	1	HB2	HB3
SER91	Light	HIS6	2.44	-0.06	0	0	O	HD2
SER93	Light	PHE4	2.50	-4.26	0	0	HA	O
SER93	Light	ARG5	2.54	-4.58	0	1	HA	HA
TYR32	Light	HIS6	2.65	-4.00	0	0	OH	HB2
SER93	Light	HIS6	3.80	-1.67	0	0	HA	H
TYR32	Light	ASP7	3.80	-0.01	0	-2	HH	OXT
PRO95	Light	GLU3	4.05	-0.64	0	-1	HD2	OE1
TYR92	Light	PHE4	4.16	-1.57	0	0	O	HD1
THR94	Light	ARG5	4.17	-0.94	0	1	H	HA
LEU96	Light	PHE4	4.30	-0.38	0	0	HD22	HD1
TYR92	Light	ASP7	4.63	0.32	0	-2	HE1	N
ASP1	Light	GLU3	4.65	-0.26	0	-1	OD1	H
PRO95	Light	ALA2	4.65	-0.75	0	1	HD2	HA
GLN27	Light	ALA2	5.03	0.01	0	1	HE22	H3
ILE2	Light	ALA2	5.05	-0.43	0	1	H	HB2
SER93	Light	GLU3	5.10	0.41	0	-1	HB2	H
SER91	Light	PHE4	5.24	-0.21	0	0	O	HE1
ASP1	Light	ARG5	5.38	-0.65	0	1	OD2	HD3
GLN27	Light	ARG5	5.40	0.00	0	1	HE22	HH22
TYR32	Light	ARG5	5.53	-0.48	0	1	HE2	HG3
LEU96	Light	HIS6	5.64	-0.14	0	0	HD23	HE2
PRO95	Light	PHE4	5.76	-0.13	0	0	HD2	H
ASN34	Light	HIS6	5.77	-0.03	0	0	HD22	HE2
SER91	Light	ARG5	5.77	-0.01	0	1	O	HA
TYR92	Light	ALA2	5.78	-0.17	0	1	O	HB3
ILE2	Light	ARG5	5.81	-0.07	0	1	HD13	HD3
THR94	Light	HIS6	6.02	-0.08	0	0	H	H
LEU96	Light	ARG5	6.12	-0.07	0	1	HD22	HA
GLN90	Light	ALA2	6.14	-0.11	0	1	HE22	HB2
SER30	Light	HIS6	6.26	-0.08	0	0	HB3	HB3
GLN90	Light	HIS6	6.28	-0.11	0	0	OE1	HD2
ASP1	Light	PHE4	6.61	-0.07	0	0	OD1	H
GLN90	Light	PHE4	6.70	-0.06	0	0	HE22	O
LEU96	Light	ALA2	6.71	-0.07	0	1	HD21	HB1
ILE29	Light	HIS6	6.83	-0.10	0	0	O	HD2
TYR32	Light	PHE4	6.95	-0.08	0	0	HE1	HE1
GLN90	Light	ARG5	7.03	-0.15	0	1	OE1	HA
ILE29	Light	ARG5	7.08	-0.09	0	1	HA	HD2
SER30	Light	ARG5	7.10	-0.04	0	1	HB3	HD2
SER28	Light	ARG5	7.24	-0.21	0	1	O	HD2
ALA50	Light	HIS6	7.36	-0.01	0	0	HB3	HE2
ILE2	Light	PHE4	7.61	-0.03	0	0	HG12	O

LEU33	Light	HIS6	7.77	-0.06	0	0	N	HD2
LEU96	Light	GLU3	7.82	-0.07	0	-1	HG	HB3
SER31	Light	HIS6	7.85	-0.07	0	0	C	HD2
ILE2	Light	GLU3	7.89	0.04	0	-1	H	H

Table S14 – Quantum biochemistry details of RC MD2-#2

Adu Residue	Adu Chain	Aβ Residue	Distance (Å)	Interaction Energy (kcal/mol)	Adu Residue Charge	Aβ Residue Charge	Adu Atom	Aβ Atom
ARG105	Heavy	ARG5	1.68	-3.02	1	1	HH12	O
LYS65	Heavy	GLU3	1.94	-10.33	1	-1	HZ3	OE2
ARG105	Heavy	ASP7	1.94	-14.36	1	-2	HH21	OD2
PRO108	Heavy	HIS6	2.01	-5.56	0	0	O	HE2
GLY107	Heavy	HIS6	2.11	-1.21	0	0	O	HE1
ARG105	Heavy	PHE4	2.16	-2.24	1	0	HD2	HE2
GLY107	Heavy	PHE4	2.23	-0.72	0	0	HA3	HE1
TYR59	Heavy	GLU3	2.35	-2.82	0	-1	HD1	HB2
PRO108	Heavy	PHE4	2.41	-1.90	0	0	HD3	HE1
TYR59	Heavy	PHE4	2.55	-5.21	0	0	OH	HD2
ILE102	Heavy	HIS6	2.55	-1.33	0	0	HG21	HE1
TRP52	Heavy	PHE4	2.69	-3.50	0	0	HZ2	HZ
ARG105	Heavy	HIS6	2.84	-2.74	1	0	HH12	HA
TYR109	Heavy	HIS6	2.85	-2.25	0	0	HA	HE2
TYR60	Heavy	GLU3	3.78	0.71	0	-1	O	OE1
ASP62	Heavy	GLU3	4.14	2.39	-1	-1	OD2	OE1
GLY101	Heavy	HIS6	4.25	0.17	0	0	HA2	HE1
ARG106	Heavy	PHE4	4.54	-0.06	1	0	C	HZ
ARG106	Heavy	HIS6	4.89	0.04	1	0	C	HE1
LYS57	Heavy	PHE4	5.06	-0.29	1	0	HG3	HE2
THR61	Heavy	GLU3	5.25	0.19	0	-1	HA	OE1
VAL50	Heavy	PHE4	5.38	-0.21	0	0	HG12	HB2
GLY103	Heavy	HIS6	5.48	-0.08	0	0	N	HE1
GLY107	Heavy	ARG5	5.48	-0.24	0	1	HA3	O
TRP47	Heavy	GLU3	5.58	-0.06	0	-1	HH2	HB3
ILE102	Heavy	PHE4	5.68	-0.13	0	0	O	HZ
TRP47	Heavy	PHE4	5.77	-0.15	0	0	HH2	HB2
TRP52	Heavy	ARG5	5.91	-0.13	0	1	HH2	H
TYR110	Heavy	HIS6	6.00	-0.15	0	0	N	HE2
TRP52	Heavy	HIS6	6.14	-0.10	0	0	HZ2	HE1
LYS58	Heavy	GLU3	6.14	-1.05	1	-1	O	HB2
GLY103	Heavy	ASP7	6.18	-0.20	0	-2	HA3	H
TYR59	Heavy	ARG5	6.19	-0.28	0	1	HE1	H
TYR59	Heavy	ALA2	6.27	-0.01	0	1	HE1	O
PRO108	Heavy	ARG5	6.29	-0.49	0	1	HD3	HA
ASP99	Heavy	HIS6	6.29	-0.26	-1	0	OD2	HE2
LYS58	Heavy	PHE4	6.32	-0.20	1	0	O	HB3
ILE102	Heavy	ASP7	6.38	-0.07	0	-2	HG22	H

GLY101	Heavy	PHE4	6.43	-0.02	0	0	HA2	HE1
GLY107	Heavy	ASP7	6.46	0.03	0	-2	HA3	H
TYR109	Heavy	PHE4	6.62	-0.11	0	0	N	HE1
ALA104	Heavy	ASP7	6.91	-0.38	0	-2	H	HB3
GLY103	Heavy	PHE4	6.93	-0.03	0	0	HA3	HZ
TRP47	Heavy	ALA2	6.99	-0.17	0	1	HH2	HB3
ALA104	Heavy	PHE4	7.12	0.05	0	0	C	HZ
HIS35	Heavy	PHE4	7.20	-0.03	0	0	HE1	HD1
ARG105	Heavy	GLU3	7.24	-0.75	1	-1	HH12	O
LYS57	Heavy	GLU3	7.24	-1.14	1	-1	HD2	O
TYR60	Heavy	PHE4	7.25	-0.08	0	0	H	HB3
HIS35	Heavy	HIS6	7.34	0.01	0	0	HE1	HE2
LYS65	Heavy	ALA2	7.35	1.46	1	1	HZ1	HA
ARG100	Heavy	HIS6	7.35	0.01	1	0	C	HE1
LYS65	Heavy	PHE4	7.38	-0.24	1	0	HZ3	H
VAL50	Heavy	GLU3	7.39	0.02	0	-1	HG13	HB3
TRP52	Heavy	GLU3	7.40	0.01	0	-1	HH2	O
ASP62	Heavy	ALA2	7.45	-1.37	-1	1	OD2	HA
ALA104	Heavy	HIS6	7.66	-0.04	0	0	H	ND1
PHE53	Heavy	PHE4	7.73	-0.05	0	0	HE2	HZ
ILE102	Heavy	ARG5	7.87	-0.02	0	1	HG22	O
ASP1	Light	ALA2	1.71	-11.31	0	1	OD2	H
TYR92	Light	HIS6	1.96	-5.15	0	0	O	H
THR94	Light	PHE4	2.08	-6.29	0	0	H	O
SER93	Light	ARG5	2.23	-4.42	0	1	HA	HA
THR94	Light	GLU3	2.32	-1.87	0	-1	HG1	H
SER93	Light	ALA2	2.39	-1.55	0	1	HB2	HB2
THR94	Light	ALA2	2.42	-0.76	0	1	H	HB3
TYR92	Light	ARG5	2.49	-7.98	0	1	O	HA
SER93	Light	PHE4	2.66	-3.67	0	0	HA	O
SER91	Light	HIS6	2.75	-0.65	0	0	O	HE2
TYR32	Light	HIS6	2.79	-4.80	0	0	CD1	HD2
SER93	Light	HIS6	3.70	-1.54	0	0	HA	H
THR94	Light	ARG5	3.95	-1.58	0	1	H	HA
LEU96	Light	PHE4	4.28	-0.41	0	0	HD22	HD1
TYR92	Light	ASP7	4.52	0.31	0	-2	HH	OXT
PRO95	Light	ALA2	4.53	-0.68	0	1	N	HB3
TYR92	Light	PHE4	4.60	-1.58	0	0	O	HD1
PRO95	Light	GLU3	4.61	-0.48	0	-1	HD2	H
ILE2	Light	ALA2	4.85	-0.37	0	1	HD12	HB1
TYR32	Light	ASP7	5.04	-0.31	0	-2	HH	O
ASP1	Light	GLU3	5.49	-0.01	0	-1	OD1	H
SER93	Light	GLU3	5.55	0.47	0	-1	HB2	H
LEU96	Light	ARG5	5.69	-0.09	0	1	HD22	HA
ASP1	Light	ARG5	5.74	-0.38	0	1	OD2	HB2
ILE29	Light	HIS6	5.78	-0.18	0	0	O	HD2
GLN90	Light	ALA2	5.83	-0.04	0	1	HE22	HB1
LEU96	Light	ALA2	5.86	-0.09	0	1	HD21	HB3

THR94	Light	HIS6	5.90	-0.06	0	0	H	H
PRO95	Light	PHE4	5.93	-0.09	0	0	N	H
ASN34	Light	HIS6	5.99	-0.03	0	0	HD22	HE2
SER30	Light	HIS6	5.99	-0.10	0	0	HB3	HB3
SER91	Light	PHE4	6.03	-0.10	0	0	O	HE1
SER91	Light	ARG5	6.10	0.22	0	1	O	HA
LEU96	Light	HIS6	6.15	-0.12	0	0	HD11	HE2
TYR92	Light	ALA2	6.18	-0.08	0	1	O	HB2
ASP1	Light	PHE4	6.35	-0.05	0	0	OD2	O
SER30	Light	ASP7	6.45	-0.11	0	-2	HB2	OXT
GLN90	Light	HIS6	6.48	-0.15	0	0	OE1	HD2
ILE2	Light	ARG5	6.55	-0.06	0	1	HD11	HB3
GLN90	Light	PHE4	6.67	-0.06	0	0	HE22	O
TYR32	Light	ARG5	6.74	-0.31	0	1	HE2	HH12
GLN90	Light	ARG5	6.94	-0.10	0	1	OE1	HA
SER31	Light	HIS6	6.99	-0.11	0	0	H	HD2
ILE2	Light	PHE4	7.07	-0.03	0	0	HD13	O
SER30	Light	ARG5	7.11	-0.01	0	1	HB2	HH12
LEU33	Light	HIS6	7.30	-0.07	0	0	N	HD2
ALA50	Light	HIS6	7.30	-0.02	0	0	HB2	HE2
ILE2	Light	HIS6	7.34	-0.06	0	0	HD13	H
SER93	Light	ASP7	7.34	-0.03	0	-2	HB3	N
ILE29	Light	ARG5	7.39	-0.07	0	1	HA	HB3
LEU96	Light	GLU3	7.50	-0.06	0	-1	HD21	H
TYR32	Light	PHE4	7.59	-0.05	0	0	HE1	HE1
SER28	Light	ARG5	7.82	-0.20	0	1	O	HH12

Table S15 – Quantum biochemistry details of RC MD2-#3

Adu Residue	Adu Chain	Aβ Residue	Distance (Å)	Interaction Energy (kcal/mol)	Adu Residue Charge	Aβ Residue Charge	Adu Atom	Aβ Atom
LYS65	Heavy	GLU3	1.59	-12.01	1	-1	HZ3	OE2
PRO108	Heavy	HIS6	1.64	-5.31	0	0	O	HE2
ARG105	Heavy	ARG5	1.73	-2.63	1	1	HH11	O
ILE102	Heavy	HIS6	2.15	-0.64	0	0	HG21	HE1
TYR59	Heavy	ALA2	2.44	-0.68	0	1	HE1	O
GLY107	Heavy	HIS6	2.45	-2.51	0	0	O	HE1
TYR59	Heavy	PHE4	2.60	-4.26	0	0	CZ	HB3
ARG105	Heavy	HIS6	2.73	-3.82	1	0	HH11	HA
ARG105	Heavy	PHE4	2.83	-3.14	1	0	HD2	HE2
GLY107	Heavy	PHE4	2.90	-1.39	0	0	HA3	HE1
ARG105	Heavy	ASP7	2.94	-5.25	1	-2	HH12	H
PRO108	Heavy	PHE4	3.02	-1.59	0	0	HD3	HE1
TYR59	Heavy	GLU3	3.14	-2.22	0	-1	HD1	HA
TRP52	Heavy	PHE4	3.38	-2.69	0	0	HZ2	HE2
TYR60	Heavy	GLU3	3.47	3.47	0	-1	O	OE2
TYR109	Heavy	HIS6	3.49	-3.45	0	0	HA	HE2

LYS57	Heavy	PHE4	3.62	-0.60	1	0	HD3	HD2
ARG106	Heavy	HIS6	4.09	-0.69	1	0	C	HE1
GLY103	Heavy	HIS6	4.09	-0.41	0	0	HA3	HE1
ASP62	Heavy	GLU3	4.12	2.06	-1	-1	OD1	OE2
THR61	Heavy	GLU3	4.57	0.64	0	-1	HA	OE2
GLY101	Heavy	HIS6	4.61	-0.05	0	0	HA2	HE2
ALA104	Heavy	ASP7	5.08	-0.39	0	-2	H	OD1
ARG106	Heavy	PHE4	5.13	0.03	1	0	H	HZ
GLY103	Heavy	ASP7	5.14	-0.62	0	-2	HA2	OD1
TRP47	Heavy	GLU3	5.18	-0.14	0	-1	HH2	OE1
ILE102	Heavy	PHE4	5.28	-0.17	0	0	O	HZ
GLY103	Heavy	PHE4	5.56	-0.05	0	0	HA3	HZ
LYS65	Heavy	ALA2	5.78	1.46	1	1	HE3	HA
ILE102	Heavy	ASP7	5.91	-0.13	0	-2	HG22	H
LYS58	Heavy	GLU3	5.99	-0.90	1	-1	O	HA
TRP52	Heavy	HIS6	6.03	-0.17	0	0	HH2	HE1
LYS58	Heavy	ALA2	6.07	0.65	1	1	O	O
ASP99	Heavy	HIS6	6.12	-0.27	-1	0	OD1	HE2
VAL50	Heavy	PHE4	6.15	-0.10	0	0	HG11	HB2
ARG105	Heavy	GLU3	6.17	-0.85	1	-1	HH21	O
LYS58	Heavy	PHE4	6.27	-0.15	1	0	O	HB3
TRP52	Heavy	ARG5	6.28	-0.08	0	1	HH2	H
TYR59	Heavy	ARG5	6.30	-0.20	0	1	OH	H
PRO108	Heavy	ARG5	6.36	-0.36	0	1	HD3	HA
TYR110	Heavy	HIS6	6.40	-0.13	0	0	N	HE2
GLY107	Heavy	ARG5	6.41	-0.13	0	1	HA3	C
GLY107	Heavy	ASP7	6.44	0.05	0	-2	H	H
LYS57	Heavy	ALA2	6.58	0.95	1	1	HB2	O
ALA104	Heavy	HIS6	6.59	-0.07	0	0	N	HE1
TRP47	Heavy	PHE4	6.63	-0.08	0	0	HH2	HB2
TYR60	Heavy	PHE4	6.82	-0.02	0	0	H	H
VAL50	Heavy	GLU3	6.99	0.02	0	-1	HG12	OE1
TYR109	Heavy	PHE4	7.06	-0.09	0	0	N	HE1
ARG106	Heavy	ASP7	7.10	-1.39	1	-2	H	H
LYS65	Heavy	PHE4	7.14	-0.22	1	0	HZ3	H
LYS57	Heavy	ARG5	7.19	0.62	1	1	HD3	H
ALA104	Heavy	PHE4	7.24	0.05	0	0	C	HZ
HIS35	Heavy	HIS6	7.28	-0.01	0	0	HE1	HE2
GLY101	Heavy	PHE4	7.40	-0.02	0	0	HA2	HE1
LYS57	Heavy	GLU3	7.41	-0.97	1	-1	HB2	HA
TYR60	Heavy	ALA2	7.43	-0.25	0	1	H	C
ARG100	Heavy	HIS6	7.52	0.03	1	0	C	HE1
HIS35	Heavy	PHE4	7.59	-0.02	0	0	HE1	HD1
ARG105	Heavy	ALA2	7.73	0.76	1	1	HH21	O
VAL48	Heavy	GLU3	7.86	0.12	0	-1	O	OE1
TRP52	Heavy	GLU3	7.91	-0.03	0	-1	HH2	C
SER63	Heavy	GLU3	7.98	0.03	0	-1	H	OE2
THR94	Light	GLU3	1.65	-11.79	0	-1	HG1	OE1

TYR92	Light	HIS6	1.97	-7.00	0	0	O	H
THR94	Light	PHE4	2.01	-6.89	0	0	OG1	H
SER93	Light	ARG5	2.01	-4.61	0	1	HB3	HG3
TYR92	Light	ASP7	2.30	-2.43	0	-2	HH	OXT
TYR92	Light	ARG5	2.39	-8.32	0	1	HE2	HG3
GLN27	Light	ARG5	2.41	-1.55	0	1	OE1	HH11
SER93	Light	PHE4	2.51	-3.07	0	0	HA	O
TYR32	Light	HIS6	2.62	-4.06	0	0	HE1	HB2
SER91	Light	HIS6	2.77	-0.74	0	0	O	HD2
PRO95	Light	GLU3	2.81	-1.74	0	-1	HD2	HG3
SER93	Light	HIS6	3.39	-2.47	0	0	HB3	H
THR94	Light	ARG5	3.65	-1.91	0	1	H	HA
TYR92	Light	PHE4	3.67	-1.95	0	0	O	HE1
TYR32	Light	ASP7	3.98	-0.68	0	-2	HE1	H
SER30	Light	ASP7	4.01	-0.41	0	-2	HB2	O
ILE2	Light	ARG5	4.07	-0.15	0	1	HD12	HH12
SER30	Light	HIS6	4.23	-0.34	0	0	HB3	HB3
LEU96	Light	PHE4	4.49	-0.27	0	0	HD22	HD1
SER93	Light	GLU3	4.82	-0.36	0	-1	HB2	HB2
SER91	Light	PHE4	4.86	-0.27	0	0	O	HE1
THR94	Light	HIS6	5.42	-0.16	0	0	H	H
LEU96	Light	HIS6	5.51	-0.11	0	0	HD22	HD2
PRO95	Light	PHE4	5.59	-0.04	0	0	N	H
THR94	Light	ALA2	5.67	-0.26	0	1	HG1	C
SER28	Light	ARG5	5.80	-0.37	0	1	O	HH11
TYR32	Light	PHE4	5.89	-0.10	0	0	HD1	HE1
SER31	Light	HIS6	5.90	-0.12	0	0	H	HB3
LEU96	Light	ARG5	5.91	-0.09	0	1	HD22	HA
ILE29	Light	HIS6	6.00	-0.16	0	0	O	HB3
SER91	Light	ARG5	6.21	0.16	0	1	O	HA
SER31	Light	ASP7	6.34	-0.26	0	-2	H	O
ILE29	Light	ARG5	6.38	-0.06	0	1	HA	HG3
GLN90	Light	HIS6	6.38	-0.14	0	0	OE1	HD2
LEU96	Light	GLU3	6.50	-0.02	0	-1	H	OE1
ASP1	Light	GLU3	6.57	0.24	0	-1	OD2	HG3
LEU33	Light	HIS6	6.70	-0.13	0	0	N	HD2
TYR32	Light	ARG5	6.74	-0.08	0	1	HD1	C
GLN90	Light	PHE4	6.92	-0.06	0	0	HE22	O
GLN90	Light	ARG5	6.99	-0.07	0	1	OE1	HA
SER30	Light	ARG5	7.05	-0.05	0	1	HB2	HG2
ASN34	Light	HIS6	7.14	-0.03	0	0	HD22	HE2
ILE2	Light	PHE4	7.16	-0.03	0	0	HD13	O
SER93	Light	ASP7	7.29	0.17	0	-2	HB3	N
ILE2	Light	HIS6	7.37	-0.04	0	0	HD13	H
ILE29	Light	ASP7	7.45	-0.21	0	-2	C	O
SER28	Light	ASP7	7.58	0.19	0	-2	OG	O
PRO95	Light	ALA2	7.70	-0.21	0	1	HD2	C
ASP1	Light	ARG5	7.71	0.11	0	1	HA	HH12

GLN90	Light	GLU3	7.84	-0.01	0	-1	HE22	HB2
PRO95	Light	ARG5	7.91	-0.18	0	1	N	HA
TYR92	Light	GLU3	7.92	0.00	0	-1	O	C
SER28	Light	HIS6	7.94	-0.01	0	0	O	H
SER26	Light	ARG5	7.96	0.10	0	1	HG	HH11

Table S16 – Quantum biochemistry details of RC MD2-#4

**Adu Residue**	**Adu Chain**	**Aβ Residue**	**Distance (Å)**	**Interaction Energy (kcal/mol)**	**Adu Residue Charge**	**Aβ Residue Charge**	**Adu Atom**	**Aβ Atom**
LYS65	Heavy	GLU3	1.55	-10.95	1	-1	HZ1	OE1
PRO108	Heavy	HIS6	1.65	-5.56	0	0	O	HE2
GLY107	Heavy	HIS6	1.82	-0.49	0	0	HA3	HE1
PRO108	Heavy	PHE4	2.38	-1.44	0	0	HD3	HE1
ARG105	Heavy	PHE4	2.46	-2.07	1	0	HD2	HE2
TYR59	Heavy	GLU3	2.54	-3.45	0	-1	HE1	HB2
TRP52	Heavy	PHE4	2.70	-3.56	0	0	HZ2	HE2
GLY107	Heavy	PHE4	2.74	-0.96	0	0	HA3	HZ
ILE102	Heavy	HIS6	2.74	-2.27	0	0	HG22	HE1
TYR59	Heavy	PHE4	2.83	-4.40	0	0	CZ	HB3
ARG105	Heavy	HIS6	3.11	-1.47	1	0	HH11	O
TYR109	Heavy	HIS6	3.79	-2.23	0	0	HA	HE2
TYR60	Heavy	GLU3	3.82	0.52	0	-1	O	OE1
ARG105	Heavy	ASP7	3.84	-2.77	1	-2	HH11	O
ARG105	Heavy	ARG5	4.04	0.85	1	1	HH21	O
ARG106	Heavy	HIS6	4.48	0.19	1	0	C	HE1
GLY101	Heavy	HIS6	5.23	-0.06	0	0	HA2	HE2
LYS57	Heavy	PHE4	5.36	-0.29	1	0	HG3	HE2
VAL50	Heavy	PHE4	5.43	-0.18	0	0	HG11	HB2
ARG106	Heavy	PHE4	5.52	0.09	1	0	C	HZ
ILE102	Heavy	PHE4	5.65	-0.11	0	0	HG22	HE1
TRP52	Heavy	HIS6	5.81	-0.18	0	0	HH2	HE1
THR61	Heavy	GLU3	5.84	0.29	0	-1	HA	OE1
PRO108	Heavy	ARG5	5.98	-0.40	0	1	HD3	HA
ASP62	Heavy	GLU3	6.00	1.46	-1	-1	OD2	OE1
TRP52	Heavy	ARG5	6.11	-0.14	0	1	HH2	N
TRP47	Heavy	GLU3	6.15	-0.03	0	-1	HH2	OE1
LYS58	Heavy	GLU3	6.18	-1.04	1	-1	O	HB2
ASP99	Heavy	HIS6	6.21	-0.18	-1	0	OD2	HE2
TYR59	Heavy	ARG5	6.47	-0.19	0	1	OH	H
LYS65	Heavy	ALA2	6.54	1.65	1	1	HZ1	HA
TRP47	Heavy	PHE4	6.56	-0.08	0	0	HH2	HB2
TYR110	Heavy	HIS6	6.66	-0.11	0	0	N	HE2
TYR59	Heavy	ALA2	6.67	-0.03	0	1	HE1	C
HIS35	Heavy	HIS6	6.68	-0.02	0	0	HE1	HE2
GLY107	Heavy	ARG5	6.71	-0.10	0	1	HA3	O
LYS58	Heavy	PHE4	6.72	-0.14	1	0	O	HB3

TYR109	Heavy	PHE4	7.00	-0.08	0	0	N	HE1
GLY103	Heavy	HIS6	7.01	-0.10	0	0	N	HE1
TRP52	Heavy	GLU3	7.07	-0.04	0	-1	HH2	C
HIS35	Heavy	PHE4	7.20	-0.03	0	0	HE1	HD1
LYS65	Heavy	PHE4	7.21	-0.16	1	0	HZ1	H
TYR60	Heavy	PHE4	7.31	-0.06	0	0	H	H
LYS57	Heavy	GLU3	7.34	-1.16	1	-1	HD2	O
ASP62	Heavy	ALA2	7.39	-1.32	-1	1	OD2	HA
GLY101	Heavy	PHE4	7.44	0.00	0	0	HA2	HE1
VAL50	Heavy	GLU3	7.56	0.03	0	-1	HG12	HB3
ARG105	Heavy	GLU3	7.67	-0.69	1	-1	HH21	O
ILE102	Heavy	ASP7	7.87	-0.03	0	-2	HG21	N
TYR60	Heavy	ALA2	7.90	-0.25	0	1	O	HA
ILE102	Heavy	ARG5	7.90	-0.04	0	1	HG21	C
TRP47	Heavy	ALA2	7.92	-0.14	0	1	HH2	HB3
VAL50	Heavy	HIS6	7.92	-0.01	0	0	HG22	HE2
PHE53	Heavy	PHE4	7.98	-0.07	0	0	HE1	HZ
ASP1	Light	ALA2	1.69	-9.00	0	1	OD1	H3
THR94	Light	PHE4	1.88	-5.34	0	0	OG1	H
SER93	Light	ARG5	2.18	-4.03	0	1	HB2	HB2
TYR92	Light	ASP7	2.27	-5.28	0	-2	HH	OD1
THR94	Light	GLU3	2.29	-2.97	0	-1	HG1	H
TYR92	Light	HIS6	2.41	-6.09	0	0	O	H
TYR92	Light	ARG5	2.51	-8.01	0	1	OH	HD2
SER93	Light	PHE4	2.54	-5.44	0	0	HA	O
THR94	Light	ALA2	2.60	-1.28	0	1	HG1	HB3
SER93	Light	ALA2	2.81	-1.50	0	1	HB2	HB2
TYR32	Light	HIS6	3.01	-4.68	0	0	HE2	HB3
SER91	Light	HIS6	3.26	-0.46	0	0	O	HD2
SER93	Light	HIS6	3.72	-0.87	0	0	HB3	H
THR94	Light	ARG5	3.73	-1.49	0	1	H	HA
ASP1	Light	ARG5	3.99	-0.94	0	1	OD2	HD3
LEU96	Light	PHE4	4.16	-0.38	0	0	HD21	HD1
PRO95	Light	ALA2	4.28	-0.52	0	1	HD2	HA
TYR92	Light	PHE4	4.37	-1.79	0	0	O	O
PRO95	Light	GLU3	4.53	-0.70	0	-1	HD2	H
ILE2	Light	ALA2	5.12	-0.41	0	1	H	HB1
SER93	Light	ASP7	5.35	-0.22	0	-2	HB3	H
LEU96	Light	ARG5	5.37	-0.12	0	1	HD21	HA
ASP1	Light	GLU3	5.44	0.29	0	-1	OD1	H
SER30	Light	ASP7	5.49	0.00	0	-2	HG	OD1
SER91	Light	PHE4	5.50	-0.15	0	0	O	HE1
TYR32	Light	ASP7	5.52	-0.24	0	-2	HE2	OD2
ILE2	Light	ARG5	5.66	-0.12	0	1	HD13	HD2
GLN27	Light	ARG5	5.75	0.05	0	1	HG2	HH22
SER91	Light	ARG5	5.76	0.02	0	1	O	HA
SER93	Light	GLU3	5.81	0.52	0	-1	HB2	H
GLN90	Light	ALA2	5.92	-0.03	0	1	HE22	HB1

PRO95	Light	PHE4	5.93	-0.15	0	0	N	H
LEU96	Light	HIS6	5.94	-0.12	0	0	HD21	HE2
THR94	Light	HIS6	5.96	-0.12	0	0	H	H
ASP1	Light	PHE4	6.01	-0.08	0	0	OD2	O
SER30	Light	HIS6	6.11	-0.09	0	0	HB2	HB3
SER28	Light	ARG5	6.13	-0.26	0	1	O	HD2
ILE29	Light	HIS6	6.21	-0.15	0	0	O	HD2
TYR92	Light	ALA2	6.33	-0.20	0	1	O	HB2
SER28	Light	ASP7	6.44	0.20	0	-2	HG	OD1
LEU96	Light	ALA2	6.50	-0.08	0	1	HD23	HB3
GLN90	Light	PHE4	6.55	-0.06	0	0	HE22	O
ILE29	Light	ARG5	6.81	-0.10	0	1	HG22	HB3
GLN90	Light	ARG5	6.89	-0.11	0	1	OE1	HA
TYR32	Light	ARG5	6.94	-0.25	0	1	HD2	HB3
GLN90	Light	HIS6	6.96	-0.12	0	0	OE1	HD2
ILE29	Light	ASP7	7.02	-0.05	0	-2	HA	OD1
ILE2	Light	PHE4	7.04	-0.03	0	0	HG12	O
ASN34	Light	HIS6	7.23	-0.02	0	0	HD22	HE2
SER31	Light	HIS6	7.34	-0.08	0	0	H	HD2
TYR32	Light	PHE4	7.59	-0.06	0	0	HE1	HE1
LEU33	Light	HIS6	7.59	-0.05	0	0	N	HD2
SER28	Light	HIS6	7.63	-0.05	0	0	O	H
ILE2	Light	HIS6	7.73	-0.03	0	0	HG13	H
PRO95	Light	ARG5	7.83	-0.16	0	1	N	HB2
THR97	Light	ALA2	7.88	-0.06	0	1	H	HB1
THR94	Light	ASP7	7.97	0.22	0	-2	H	H

Table S17 – Quantum biochemistry details of RC MD2-#5

Adu Residue	Adu Chain	Aβ Residue	Distance (Å)	Interaction Energy (kcal/mol)	Adu Residue Charge	Aβ Residue Charge	Adu Atom	Aβ Atom
LYS65	Heavy	GLU3	1.63	-10.71	1	-1	HZ3	OE1
ARG105	Heavy	ASP7	1.78	-16.00	1	-2	HH12	OD2
PRO108	Heavy	HIS6	2.06	-4.61	0	0	O	HE2
TYR59	Heavy	PHE4	2.38	-4.28	0	0	CE2	HB3
GLY107	Heavy	HIS6	2.40	-2.64	0	0	O	HE1
ILE102	Heavy	HIS6	2.42	0.06	0	0	O	HE1
TYR59	Heavy	ALA2	2.66	-0.50	0	1	HE1	O
ARG105	Heavy	PHE4	2.76	-1.57	1	0	HH21	HD2
GLY107	Heavy	PHE4	2.83	-1.58	0	0	HA3	CZ
PRO108	Heavy	PHE4	2.85	-2.18	0	0	HD3	CD1
TRP52	Heavy	PHE4	2.90	-3.52	0	0	HH2	HB3
ARG105	Heavy	ARG5	2.97	0.87	1	1	HH12	O
TYR59	Heavy	GLU3	3.04	-2.76	0	-1	HD1	HA
TYR109	Heavy	HIS6	3.22	-2.12	0	0	HA	HE2
ALA104	Heavy	ASP7	3.55	-0.96	0	-2	HB2	OD1
ARG105	Heavy	HIS6	3.99	-0.22	1	0	HH12	HA

ASP62	Heavy	GLU3	4.02	2.38	-1	-1	OD1	OE1
GLY103	Heavy	HIS6	4.08	-0.04	0	0	HA3	ND1
TYR60	Heavy	GLU3	4.19	1.84	0	-1	O	OE1
ARG106	Heavy	HIS6	4.25	-0.37	1	0	C	HE1
TRP47	Heavy	GLU3	4.27	-0.16	0	-1	HH2	OE2
GLY101	Heavy	HIS6	4.46	0.14	0	0	HA2	HE1
ILE102	Heavy	PHE4	4.66	-0.28	0	0	O	HZ
THR61	Heavy	GLU3	4.92	0.04	0	-1	HA	OE2
GLY103	Heavy	ASP7	4.98	-0.45	0	-2	HA3	H
VAL50	Heavy	PHE4	5.17	-0.19	0	0	HG12	HB2
ARG106	Heavy	PHE4	5.29	0.09	1	0	H	HZ
GLY103	Heavy	PHE4	5.45	-0.06	0	0	HA3	HZ
TYR59	Heavy	ARG5	5.55	-0.23	0	1	HH	H
ARG105	Heavy	GLU3	5.60	-0.99	1	-1	HH11	O
TRP52	Heavy	HIS6	5.72	-0.16	0	0	HZ2	HE1
TRP52	Heavy	ARG5	5.85	-0.11	0	1	HH2	H
TRP47	Heavy	PHE4	5.95	-0.12	0	0	HH2	HB2
LYS57	Heavy	PHE4	5.98	-0.11	1	0	HB2	HD2
ALA104	Heavy	HIS6	6.08	-0.17	0	0	H	ND1
LYS65	Heavy	ALA2	6.10	1.36	1	1	HE2	HA
TYR109	Heavy	PHE4	6.14	-0.07	0	0	HA	HE1
ILE102	Heavy	ASP7	6.17	-0.04	0	-2	HG21	H
LYS58	Heavy	PHE4	6.28	-0.15	1	0	C	HB3
TYR110	Heavy	HIS6	6.32	-0.16	0	0	N	HE2
PRO108	Heavy	ARG5	6.35	-0.35	0	1	HD3	N
GLY107	Heavy	ARG5	6.39	-0.13	0	1	HA3	C
ASP99	Heavy	HIS6	6.40	-0.25	-1	0	OD2	HE2
TYR60	Heavy	PHE4	6.46	-0.08	0	0	H	H
LYS65	Heavy	PHE4	6.56	-0.15	1	0	HZ3	H
ARG105	Heavy	ALA2	6.66	0.95	1	1	HH21	O
ALA104	Heavy	PHE4	6.66	-0.01	0	0	N	HZ
ARG106	Heavy	ASP7	6.66	-1.45	1	-2	H	OD1
GLY101	Heavy	PHE4	6.72	-0.03	0	0	HA2	HE1
TRP52	Heavy	GLU3	6.80	-0.01	0	-1	HH2	C
LYS58	Heavy	GLU3	6.81	-1.03	1	-1	O	HA
VAL50	Heavy	GLU3	6.92	0.01	0	-1	HG13	OE2
HIS35	Heavy	PHE4	7.09	-0.03	0	0	HE1	HD1
GLY107	Heavy	ASP7	7.24	-0.08	0	-2	H	H
LYS58	Heavy	ALA2	7.39	0.59	1	1	O	O
ARG100	Heavy	HIS6	7.43	0.02	1	0	C	HE1
HIS35	Heavy	HIS6	7.54	0.01	0	0	HE2	HE2
ALA49	Heavy	GLU3	7.63	-0.09	0	-1	HA	OE2
LYS57	Heavy	ALA2	7.74	0.87	1	1	HD2	O
TYR60	Heavy	ALA2	7.83	-0.22	0	1	H	O
ILE102	Heavy	ARG5	7.86	-0.10	0	1	HG21	C
ASP62	Heavy	ALA2	7.93	-1.16	-1	1	OD1	HA
GLY103	Heavy	ARG5	7.95	0.02	0	1	HA3	O
THR94	Light	GLU3	1.72	-11.31	0	-1	HG1	OE2

THR94	Light	PHE4	1.97	-5.93	0	0	OG1	H
TYR92	Light	HIS6	2.12	-6.52	0	0	O	H
SER93	Light	ARG5	2.37	-4.57	0	1	HB3	HG3
SER93	Light	PHE4	2.39	-1.74	0	0	HB2	O
TYR92	Light	ARG5	2.43	-9.05	0	1	OH	HD2
TYR32	Light	HIS6	2.71	-4.33	0	0	HH	HB2
PRO95	Light	GLU3	2.84	-0.73	0	-1	HD2	HG3
SER91	Light	HIS6	2.86	-0.56	0	0	O	HD2
TYR92	Light	PHE4	3.46	-2.10	0	0	O	HE1
LEU96	Light	PHE4	3.64	-0.50	0	0	HD21	HD1
SER93	Light	HIS6	3.74	-1.75	0	0	HA	H
THR94	Light	ARG5	3.90	-1.62	0	1	H	HA
TYR32	Light	ASP7	4.06	-0.24	0	-2	HH	O
SER93	Light	GLU3	4.31	-0.46	0	-1	HB2	HB2
TYR92	Light	ASP7	4.34	0.11	0	-2	HE1	N
ASP1	Light	GLU3	4.65	-0.02	0	-1	OD1	HG3
SER91	Light	PHE4	4.80	-0.26	0	0	O	HE1
ILE2	Light	ARG5	5.13	-0.08	0	1	HD11	HG3
PRO95	Light	PHE4	5.48	0.03	0	0	N	H
SER30	Light	HIS6	5.49	-0.13	0	0	HB3	HB3
THR94	Light	HIS6	5.72	-0.09	0	0	H	H
TYR32	Light	ARG5	5.82	-0.34	0	1	HE2	HG2
THR94	Light	ALA2	5.88	-0.79	0	1	HG1	C
LEU96	Light	HIS6	5.99	-0.10	0	0	HD22	HD2
LEU96	Light	ARG5	6.09	-0.07	0	1	HD21	HA
LEU96	Light	GLU3	6.27	-0.12	0	-1	H	OE2
SER91	Light	ARG5	6.38	0.12	0	1	O	HA
ILE29	Light	HIS6	6.42	-0.13	0	0	O	HD2
GLN90	Light	PHE4	6.56	-0.08	0	0	HE22	O
GLN90	Light	HIS6	6.68	-0.10	0	0	OE1	HD2
ILE29	Light	ARG5	6.72	-0.06	0	1	HA	HG3
ILE2	Light	PHE4	6.81	-0.04	0	0	HD12	O
ASN34	Light	HIS6	6.84	0.00	0	0	HD22	HE2
GLN90	Light	ARG5	6.86	-0.12	0	1	OE1	HA
TYR32	Light	PHE4	6.87	-0.13	0	0	HB2	HE1
SER30	Light	ARG5	6.96	-0.03	0	1	HB2	HD2
SER28	Light	ARG5	6.96	-0.09	0	1	O	HG3
SER31	Light	HIS6	6.98	-0.16	0	0	H	HB3
GLN90	Light	GLU3	7.37	-0.03	0	-1	HE22	HB2
ILE2	Light	HIS6	7.37	-0.05	0	0	HD12	H
SER30	Light	ASP7	7.61	-0.09	0	-2	HB2	O
LEU33	Light	HIS6	7.73	-0.04	0	0	N	HD2
PRO95	Light	ALA2	7.80	-0.41	0	1	HD2	HB3
ASP1	Light	PHE4	7.87	-0.04	0	0	HA	O
SER93	Light	ASP7	7.93	0.02	0	-2	HB3	N
TYR92	Light	GLU3	7.94	-0.03	0	-1	O	C
PRO95	Light	ARG5	7.94	-0.09	0	1	N	HA
SER93	Light	ALA2	7.95	0.08	0	1	HB2	C

Table S18 – Quantum biochemistry details of RC MD2-#6

Adu Residue	Adu Chain	Aβ Residue	Distance (Å)	Interaction Energy (kcal/mol)	Adu Residue Charge	Aβ Residue Charge	Adu Atom	Aβ Atom
LYS65	Heavy	GLU3	1.70	-11.86	1	-1	HZ2	OE2
PRO108	Heavy	HIS6	1.83	-5.15	0	0	O	HE2
ILE102	Heavy	HIS6	2.27	-1.11	0	0	HG23	HE1
GLY107	Heavy	HIS6	2.56	-1.78	0	0	O	HE1
ARG105	Heavy	PHE4	2.68	-1.79	1	0	HG3	HE2
TYR59	Heavy	GLU3	2.82	-2.14	0	-1	HD1	HA
TYR59	Heavy	PHE4	2.87	-4.13	0	0	CZ	HB3
TYR59	Heavy	ALA2	2.91	-0.63	0	1	HE1	O
PRO108	Heavy	PHE4	3.12	-1.97	0	0	HD3	HD1
TRP52	Heavy	PHE4	3.18	-3.03	0	0	HZ2	HE2
GLY107	Heavy	PHE4	3.28	-1.13	0	0	HA3	CE1
ALA104	Heavy	ASP7	3.34	-0.83	0	-2	HB2	OD1
GLY103	Heavy	HIS6	3.55	-0.49	0	0	HA3	ND1
ASP62	Heavy	GLU3	3.90	2.73	-1	-1	OD1	OE2
TYR109	Heavy	HIS6	3.99	-1.74	0	0	HA	HE2
TYR60	Heavy	GLU3	4.04	1.36	0	-1	H	OE1
GLY103	Heavy	ASP7	4.30	-0.60	0	-2	HA3	H
ARG105	Heavy	ASP7	4.46	-2.72	1	-2	HG2	OD1
GLY101	Heavy	HIS6	4.50	0.09	0	0	C	HE1
ARG106	Heavy	HIS6	4.55	-0.16	1	0	C	HE1
ARG106	Heavy	PHE4	4.60	0.21	1	0	H	HZ
TRP47	Heavy	GLU3	4.68	-0.16	0	-1	HH2	OE1
LYS65	Heavy	ALA2	4.89	1.53	1	1	HE2	H
LYS57	Heavy	ALA2	4.96	1.23	1	1	HZ3	O
ILE102	Heavy	PHE4	5.04	-0.22	0	0	O	HZ
GLY103	Heavy	PHE4	5.25	-0.09	0	0	HA3	HZ
THR61	Heavy	GLU3	5.30	0.04	0	-1	HA	OE1
LYS57	Heavy	PHE4	5.53	-0.16	1	0	HE2	HD2
ARG105	Heavy	HIS6	5.60	-0.35	1	0	HB3	HE1
ALA104	Heavy	HIS6	5.73	-0.17	0	0	H	ND1
VAL50	Heavy	PHE4	5.95	-0.14	0	0	HG11	HB2
ILE102	Heavy	ASP7	5.96	-0.15	0	-2	HG21	H
TYR59	Heavy	ARG5	5.97	-0.23	0	1	HH	H
ALA104	Heavy	PHE4	6.02	0.05	0	0	C	HZ
TRP52	Heavy	ARG5	6.08	-0.11	0	1	HH2	H
PRO108	Heavy	ARG5	6.08	-0.35	0	1	HD3	HA
ARG105	Heavy	ARG5	6.12	0.64	1	1	HG2	O
ASP99	Heavy	HIS6	6.17	-0.24	-1	0	OD2	HE2
TRP52	Heavy	HIS6	6.34	-0.13	0	0	HZ2	HE1
LYS58	Heavy	ALA2	6.59	0.65	1	1	O	O
TRP47	Heavy	PHE4	6.62	-0.07	0	0	HH2	H
TYR60	Heavy	ALA2	6.66	-0.22	0	1	H	O

TYR60	Heavy	PHE4	6.80	-0.04	0	0	H	H
TYR110	Heavy	HIS6	6.81	-0.11	0	0	H	HE2
GLY107	Heavy	ARG5	6.82	-0.05	0	1	HA3	HA
LYS57	Heavy	GLU3	6.85	-1.47	1	-1	HZ3	HA
LYS58	Heavy	GLU3	6.90	-1.03	1	-1	O	HA
LYS58	Heavy	PHE4	6.90	-0.12	1	0	O	HB3
TYR109	Heavy	PHE4	6.96	-0.09	0	0	N	HE1
LYS65	Heavy	PHE4	6.96	-0.16	1	0	HZ2	H
GLY107	Heavy	ASP7	7.00	-0.08	0	-2	H	H
ARG106	Heavy	ASP7	7.07	-1.66	1	-2	H	OD1
ARG100	Heavy	HIS6	7.17	0.00	1	0	O	HE1
GLY101	Heavy	PHE4	7.18	-0.02	0	0	HA2	HE1
TRP52	Heavy	GLU3	7.24	-0.01	0	-1	HH2	C
VAL50	Heavy	GLU3	7.25	0.03	0	-1	HG12	OE1
HIS35	Heavy	HIS6	7.31	-0.01	0	0	HE1	HE2
GLY103	Heavy	ARG5	7.40	0.01	0	1	HA3	O
HIS35	Heavy	PHE4	7.70	-0.02	0	0	HE1	HD1
VAL50	Heavy	HIS6	7.71	-0.01	0	0	HG11	HE2
PRO108	Heavy	GLU3	7.87	-0.04	0	-1	HG2	HB2
ILE102	Heavy	ARG5	7.90	-0.05	0	1	HG21	C
ASP62	Heavy	ALA2	7.95	-1.22	-1	1	OD1	HA
ALA104	Heavy	ARG5	7.96	0.07	0	1	HB2	O
THR94	Light	GLU3	1.85	-11.37	0	-1	HG1	OE1
THR94	Light	PHE4	2.05	-7.12	0	0	OG1	H
TYR92	Light	HIS6	2.05	-4.24	0	0	HD1	HB3
SER93	Light	ARG5	2.13	-4.02	0	1	HB2	HB2
TYR92	Light	ARG5	2.14	-8.81	0	1	OH	HH22
SER93	Light	PHE4	2.30	-2.87	0	0	HA	O
PRO95	Light	GLU3	2.73	-1.24	0	-1	HD2	HG3
TYR32	Light	HIS6	2.98	-3.82	0	0	HE1	HB3
SER91	Light	HIS6	2.98	-0.65	0	0	O	HD2
TYR32	Light	ASP7	3.62	-0.47	0	-2	HH	OXT
THR94	Light	ARG5	3.98	-1.51	0	1	H	HA
TYR92	Light	PHE4	3.98	-2.38	0	0	O	HD1
TYR92	Light	ASP7	4.16	0.18	0	-2	HE1	O
SER93	Light	HIS6	4.35	-0.99	0	0	HA	H
LEU96	Light	PHE4	4.38	-0.30	0	0	HD21	HD1
SER93	Light	GLU3	4.70	-0.21	0	-1	HB2	HB2
SER91	Light	PHE4	5.07	-0.23	0	0	O	HD1
SER30	Light	HIS6	5.35	-0.13	0	0	HB3	HB3
THR94	Light	ALA2	5.38	-0.78	0	1	HG1	C
PRO95	Light	PHE4	5.60	-0.06	0	0	N	H
SER91	Light	ARG5	5.60	0.02	0	1	O	HA
LEU96	Light	HIS6	5.63	-0.10	0	0	HD22	HE2
ILE2	Light	ARG5	5.64	-0.08	0	1	HD12	HD2
ILE29	Light	HIS6	5.88	-0.17	0	0	O	HB3
THR94	Light	HIS6	6.12	-0.12	0	0	H	H
LEU96	Light	ARG5	6.19	-0.07	0	1	HD21	HA

ASP1	Light	GLU3	6.25	0.02	0	-1	HA	HG3
ILE29	Light	ARG5	6.38	-0.10	0	1	HG22	HB3
SER30	Light	ASP7	6.40	-0.15	0	-2	HB2	O
GLN90	Light	PHE4	6.42	-0.08	0	0	HE22	O
GLN90	Light	HIS6	6.43	-0.13	0	0	OE1	HD2
LEU96	Light	GLU3	6.55	-0.12	0	-1	H	OE1
GLN90	Light	ARG5	6.72	-0.12	0	1	HE22	HB2
SER28	Light	ARG5	6.72	-0.18	0	1	O	HD2
SER31	Light	HIS6	6.74	-0.13	0	0	H	HB3
TYR32	Light	ARG5	6.80	-0.22	0	1	HE1	C
ILE2	Light	PHE4	7.11	-0.04	0	0	HD12	O
GLN90	Light	GLU3	7.18	-0.02	0	-1	HE22	HB2
GLN27	Light	ARG5	7.22	0.15	0	1	HB2	HD2
ASN34	Light	HIS6	7.25	-0.01	0	0	HD22	HE2
TYR32	Light	PHE4	7.32	-0.09	0	0	CZ	HE1
ILE2	Light	HIS6	7.40	-0.06	0	0	HD12	H
TYR92	Light	GLU3	7.47	0.01	0	-1	O	HB2
PRO95	Light	ALA2	7.54	-0.25	0	1	HD2	C
PRO95	Light	ARG5	7.68	-0.13	0	1	HD3	HB2
SER30	Light	ARG5	7.81	-0.02	0	1	HB2	HH22
ILE2	Light	GLU3	7.88	0.00	0	-1	HG13	HB2
LEU33	Light	HIS6	7.97	-0.05	0	0	N	HD2

Table S19 – Quantum biochemistry details of RC MD2-#7

Adu Residue	Adu Chain	Aβ Residue	Distance (Å)	Interaction Energy (kcal/mol)	Adu Residue Charge	Aβ Residue Charge	Adu Atom	Aβ Atom
LYS65	Heavy	GLU3	1.63	-11.62	1	-1	HZ1	OE1
PRO108	Heavy	HIS6	1.74	-3.87	0	0	O	HE2
ARG105	Heavy	ASP7	1.88	-12.29	1	-2	HE	OD1
ARG105	Heavy	ARG5	2.11	-1.31	1	1	HH11	O
ILE102	Heavy	HIS6	2.25	-1.53	0	0	HG22	HE1
GLY107	Heavy	HIS6	2.32	-0.65	0	0	O	HE1
ARG105	Heavy	HIS6	2.41	-0.94	1	0	HH12	HA
GLY107	Heavy	PHE4	2.47	-1.11	0	0	HA3	HE1
ARG105	Heavy	PHE4	2.61	-2.24	1	0	HH21	HE2
TYR59	Heavy	PHE4	2.63	-4.38	0	0	HH	HD2
TYR59	Heavy	GLU3	2.76	-3.20	0	-1	HE1	HB2
TRP52	Heavy	PHE4	3.08	-3.67	0	0	HZ2	HE2
PRO108	Heavy	PHE4	3.28	-1.58	0	0	HD3	HE1
TYR109	Heavy	HIS6	3.39	-2.45	0	0	HA	HE2
ASP62	Heavy	GLU3	3.83	3.18	-1	-1	OD2	OE1
ARG106	Heavy	HIS6	4.11	-0.29	1	0	C	HE1
TYR60	Heavy	GLU3	4.13	0.32	0	-1	O	OE1
GLY101	Heavy	HIS6	4.51	-0.08	0	0	HA2	HE1
ALA104	Heavy	ASP7	4.67	-0.43	0	-2	HB1	OD1
ARG106	Heavy	PHE4	4.68	0.23	1	0	C	HZ

LYS57	Heavy	PHE4	5.12	-0.24	1	0	HD2	HD2
GLY103	Heavy	HIS6	5.39	-0.05	0	0	HA3	HE1
TRP47	Heavy	GLU3	5.61	-0.04	0	-1	HH2	HB3
ILE102	Heavy	PHE4	5.65	-0.14	0	0	O	HZ
TRP52	Heavy	HIS6	5.68	-0.17	0	0	HZ2	HE1
VAL50	Heavy	PHE4	5.69	-0.15	0	0	HG11	HB2
LYS58	Heavy	GLU3	5.70	-1.13	1	-1	O	OE2
ARG105	Heavy	GLU3	5.73	-0.85	1	-1	HH21	O
GLY103	Heavy	ASP7	5.81	-0.34	0	-2	HA3	OD1
THR61	Heavy	GLU3	5.91	0.06	0	-1	HA	OE1
TRP47	Heavy	PHE4	5.94	-0.12	0	0	HH2	HB2
GLY107	Heavy	ARG5	5.96	-0.17	0	1	HA3	C
PRO108	Heavy	ARG5	6.24	-0.38	0	1	HD3	HA
ILE102	Heavy	ASP7	6.26	-0.05	0	-2	HG23	H
GLY107	Heavy	ASP7	6.33	-0.04	0	-2	HA3	H
ASP99	Heavy	HIS6	6.41	-0.22	-1	0	OD2	HE2
TYR110	Heavy	HIS6	6.45	-0.14	0	0	N	HE2
TYR59	Heavy	ARG5	6.48	-0.25	0	1	OH	H
ARG106	Heavy	ASP7	6.49	-1.51	1	-2	H	OD1
ASP62	Heavy	ALA2	6.50	-1.65	-1	1	OD1	H3
TRP52	Heavy	ARG5	6.54	-0.11	0	1	HH2	N
LYS58	Heavy	PHE4	6.57	-0.19	1	0	O	HB3
GLY103	Heavy	PHE4	6.81	-0.03	0	0	HA3	HZ
LYS57	Heavy	GLU3	6.83	-1.25	1	-1	HZ3	O
TYR59	Heavy	ALA2	6.89	-0.14	0	1	HE1	C
ALA104	Heavy	PHE4	6.93	0.00	0	0	C	HZ
LYS65	Heavy	PHE4	7.10	-0.17	1	0	HZ1	H
TYR60	Heavy	PHE4	7.17	-0.08	0	0	H	H
TYR109	Heavy	PHE4	7.23	-0.09	0	0	N	HE1
TRP52	Heavy	GLU3	7.30	-0.02	0	-1	HH2	C
GLY101	Heavy	PHE4	7.35	-0.02	0	0	HA2	HE1
ALA104	Heavy	HIS6	7.35	-0.04	0	0	N	HE1
HIS35	Heavy	HIS6	7.41	-0.01	0	0	HE1	HE2
LYS65	Heavy	ALA2	7.50	1.57	1	1	HZ1	C
ARG100	Heavy	HIS6	7.58	-0.02	1	0	C	HE1
HIS35	Heavy	PHE4	7.62	-0.02	0	0	HE1	HD1
VAL50	Heavy	GLU3	7.63	0.03	0	-1	HG12	HB3
ARG105	Heavy	ALA2	7.89	0.77	1	1	HH11	HB3
PHE53	Heavy	PHE4	7.96	-0.05	0	0	HE2	HE2
THR94	Light	PHE4	1.92	-5.30	0	0	H	O
TYR92	Light	HIS6	1.93	-6.23	0	0	O	H
THR94	Light	GLU3	2.12	-2.28	0	-1	HG1	H
SER93	Light	ARG5	2.23	-4.93	0	1	HA	HA
SER93	Light	PHE4	2.40	-4.47	0	0	HA	O
SER93	Light	ALA2	2.43	-0.02	0	1	HG	HB1
TYR92	Light	ARG5	2.46	-8.72	0	1	O	HA
SER91	Light	HIS6	2.60	-0.77	0	0	O	HD2
TYR32	Light	HIS6	2.73	-3.18	0	0	HE2	HB3

THR94	Light	ALA2	2.84	-1.97	0	1	O	HA
THR94	Light	ARG5	3.66	-1.35	0	1	H	HA
SER93	Light	HIS6	3.77	-1.74	0	0	HA	H
TYR32	Light	ASP7	4.01	-0.39	0	-2	HH	OXT
TYR92	Light	PHE4	4.13	-2.16	0	0	O	HD1
LEU96	Light	PHE4	4.17	-0.42	0	0	HD22	HD1
ASP1	Light	ALA2	4.23	-0.50	0	1	OD2	H2
TYR92	Light	ASP7	4.41	-0.29	0	-2	HE1	N
PRO95	Light	ALA2	4.51	-0.63	0	1	HD3	H2
PRO95	Light	GLU3	4.75	-0.64	0	-1	HD2	H
ILE2	Light	ALA2	5.14	-0.16	0	1	HD11	HB1
SER91	Light	PHE4	5.32	-0.16	0	0	O	HE1
LEU96	Light	HIS6	5.34	-0.17	0	0	HD23	HE2
SER30	Light	HIS6	5.41	-0.12	0	0	HB3	HB3
LEU96	Light	ARG5	5.46	-0.12	0	1	HD22	HA
THR94	Light	HIS6	5.56	-0.12	0	0	H	H
SER91	Light	ARG5	5.56	-0.03	0	1	O	HA
SER93	Light	GLU3	5.60	0.40	0	-1	OG	H
PRO95	Light	PHE4	5.83	-0.05	0	0	N	H
ILE2	Light	ARG5	5.96	-0.05	0	1	HD11	HB2
TYR92	Light	ALA2	6.07	-0.22	0	1	O	HB3
ASP1	Light	GLU3	6.12	0.22	0	-1	OD2	H
ILE29	Light	HIS6	6.40	-0.11	0	0	O	HD2
GLN90	Light	HIS6	6.44	-0.12	0	0	OE1	HD2
TYR32	Light	ARG5	6.52	-0.12	0	1	HE2	HB3
ASN34	Light	HIS6	6.76	-0.02	0	0	HD22	HE2
GLN90	Light	PHE4	6.77	-0.06	0	0	HE22	O
GLN27	Light	ALA2	6.94	-0.02	0	1	HE22	HB1
GLN90	Light	ARG5	6.97	-0.08	0	1	HE22	HB2
SER30	Light	ASP7	6.98	-0.09	0	-2	HB2	OXT
GLN90	Light	ALA2	7.03	0.00	0	1	HE22	HA
ILE29	Light	ARG5	7.07	-0.04	0	1	HA	HB3
SER31	Light	HIS6	7.21	-0.09	0	0	H	HB3
TYR32	Light	PHE4	7.35	-0.06	0	0	HE2	HE1
SER28	Light	ARG5	7.52	-0.13	0	1	O	HB3
ILE2	Light	PHE4	7.55	-0.03	0	0	HG13	O
PRO95	Light	ARG5	7.56	-0.13	0	1	N	HB2
LEU96	Light	GLU3	7.77	-0.05	0	-1	HG	HB3
LEU96	Light	ALA2	7.85	-0.08	0	1	HD22	HB3
SER30	Light	ARG5	7.97	-0.02	0	1	HB3	HB3
LEU33	Light	HIS6	8.00	-0.05	0	0	N	HD2

Table S20 – Quantum biochemistry details of RC MD2-#8

Adu Residue	Adu Chain	Aβ Residue	Distance (Å)	Interaction Energy (kcal/mol)	Adu Residue Charge	Aβ Residue Charge	Adu Atom	Aβ Atom
LYS65	Heavy	GLU3	1.65	-13.85	1	-1	HZ2	OE2

PRO108	Heavy	HIS6	1.97	-5.14	0	0	O	HE2
ARG105	Heavy	PHE4	2.00	-2.26	1	0	HB3	HE2
TYR59	Heavy	GLU3	2.11	-1.95	0	-1	HD1	HB2
ILE102	Heavy	HIS6	2.27	-0.58	0	0	HG22	HE1
GLY107	Heavy	HIS6	2.50	-2.02	0	0	O	HE1
GLY107	Heavy	PHE4	2.53	-0.51	0	0	HA3	HZ
TYR59	Heavy	PHE4	2.92	-3.47	0	0	CZ	HB3
TRP52	Heavy	PHE4	3.03	-3.83	0	0	HZ2	HE2
ALA104	Heavy	ASP7	3.07	-0.72	0	-2	HB3	OD1
PRO108	Heavy	PHE4	3.54	-1.21	0	0	HD3	HE1
ARG105	Heavy	GLU3	3.58	-0.92	1	-1	HH21	O
GLY103	Heavy	HIS6	3.60	-0.14	0	0	HA3	ND1
TYR109	Heavy	HIS6	3.62	-2.06	0	0	HA	HE2
ARG105	Heavy	ARG5	3.64	0.55	1	1	HH22	H
ARG106	Heavy	PHE4	3.93	0.39	1	0	H	HZ
TYR60	Heavy	GLU3	4.25	0.00	0	-1	H	OE1
ILE102	Heavy	PHE4	4.34	-0.27	0	0	O	HZ
ARG106	Heavy	HIS6	4.37	-0.41	1	0	C	HE1
GLY101	Heavy	HIS6	4.42	0.30	0	0	C	HE1
ARG105	Heavy	ASP7	4.44	-3.03	1	-2	HD3	OD2
GLY103	Heavy	ASP7	4.60	-0.95	0	-2	HA3	H
GLY103	Heavy	PHE4	4.67	-0.10	0	0	HA3	HZ
ASP62	Heavy	GLU3	4.94	1.75	-1	-1	OD2	OE2
VAL50	Heavy	PHE4	5.34	-0.16	0	0	HG13	HB2
ALA104	Heavy	HIS6	5.38	-0.31	0	0	H	ND1
ARG105	Heavy	HIS6	5.52	-0.47	1	0	H	HA
LYS58	Heavy	GLU3	5.59	-1.05	1	-1	O	OE1
ALA104	Heavy	PHE4	5.67	0.05	0	0	HB3	HE2
TRP52	Heavy	ARG5	6.01	-0.11	0	1	HH2	H
TRP47	Heavy	GLU3	6.09	-0.02	0	-1	HH2	HB3
LYS65	Heavy	ALA2	6.18	1.64	1	1	HZ2	HA
TRP52	Heavy	HIS6	6.20	-0.13	0	0	HZ2	HE1
ARG105	Heavy	ALA2	6.27	0.88	1	1	HH21	O
ILE102	Heavy	ASP7	6.27	-0.14	0	-2	HG23	H
TYR59	Heavy	ALA2	6.27	0.00	0	1	HD1	C
LYS57	Heavy	PHE4	6.28	-0.09	1	0	HD2	HD2
ARG106	Heavy	ASP7	6.32	-1.74	1	-2	H	OD2
TRP47	Heavy	PHE4	6.37	-0.08	0	0	HH2	HB2
THR61	Heavy	GLU3	6.38	0.10	0	-1	HA	OE2
GLY107	Heavy	ARG5	6.39	-0.20	0	1	HA3	C
LYS58	Heavy	PHE4	6.54	-0.12	1	0	O	HB3
TYR110	Heavy	HIS6	6.64	-0.11	0	0	N	HE2
TYR109	Heavy	PHE4	6.68	-0.09	0	0	N	HE1
LYS65	Heavy	PHE4	6.73	-0.17	1	0	HZ2	H
ASP99	Heavy	HIS6	6.75	-0.20	-1	0	OD1	HE2
ALA104	Heavy	ARG5	6.82	0.02	0	1	HB3	O
GLY107	Heavy	ASP7	6.87	0.06	0	-2	H	H
TYR59	Heavy	ARG5	6.88	-0.19	0	1	CZ	H

PRO108	Heavy	ARG5	6.89	-0.32	0	1	HD3	HA
TRP52	Heavy	GLU3	6.92	-0.05	0	-1	HH2	O
LYS57	Heavy	GLU3	7.03	-1.08	1	-1	HD3	O
GLY103	Heavy	ARG5	7.15	-0.01	0	1	HA3	O
PHE53	Heavy	PHE4	7.17	-0.07	0	0	HE2	HE2
GLY101	Heavy	PHE4	7.18	-0.02	0	0	HA2	HE1
VAL50	Heavy	GLU3	7.27	0.01	0	-1	HG11	HB3
TYR60	Heavy	PHE4	7.37	-0.05	0	0	H	H
HIS35	Heavy	HIS6	7.42	0.00	0	0	HE1	HE2
ARG100	Heavy	HIS6	7.47	-0.02	1	0	C	HE1
HIS35	Heavy	PHE4	7.57	-0.03	0	0	HE1	HD1
ASP62	Heavy	ALA2	7.63	-1.29	-1	1	OD2	HA
ARG106	Heavy	ARG5	7.63	0.57	1	1	H	O
TRP52	Heavy	ASP7	7.86	-0.07	0	-2	HZ2	OD2
ILE102	Heavy	ARG5	7.94	-0.07	0	1	O	O
THR94	Light	PHE4	2.20	-4.63	0	0	OG1	H
TYR92	Light	HIS6	2.25	-4.36	0	0	HA	HD2
TYR92	Light	ARG5	2.26	-4.59	0	1	HH	HH11
SER93	Light	ARG5	2.39	-3.88	0	1	HB3	HB2
THR94	Light	ALA2	2.60	-1.42	0	1	O	HB3
THR94	Light	GLU3	2.61	-2.35	0	-1	HG1	HB3
SER91	Light	HIS6	2.68	-1.00	0	0	O	HD2
SER93	Light	ALA2	2.79	-0.72	0	1	HB2	HB3
SER93	Light	PHE4	2.81	-2.46	0	0	HA	O
TYR32	Light	HIS6	3.00	-4.42	0	0	HH	HB2
THR94	Light	ARG5	4.08	-0.96	0	1	H	HA
SER93	Light	HIS6	4.14	-1.04	0	0	HB3	H
LEU96	Light	PHE4	4.22	-0.38	0	0	HD22	HD1
TYR92	Light	PHE4	4.24	-1.28	0	0	O	HD1
PRO95	Light	GLU3	4.35	-0.72	0	-1	HD2	OE2
ASP1	Light	ALA2	4.42	-0.87	0	1	OD1	HB1
SER91	Light	PHE4	4.49	-0.29	0	0	O	HE1
PRO95	Light	ALA2	4.63	-0.61	0	1	HD2	HB3
ILE2	Light	ARG5	4.80	-0.17	0	1	HD11	HH22
TYR92	Light	ASP7	5.05	0.44	0	-2	HE1	N
TYR32	Light	ASP7	5.17	-0.18	0	-2	HH	H
ILE2	Light	ALA2	5.26	-0.14	0	1	HG13	HB1
SER30	Light	HIS6	5.46	-0.15	0	0	HB3	HB3
SER93	Light	GLU3	5.60	0.28	0	-1	HB2	H
LEU96	Light	HIS6	5.62	-0.12	0	0	HD22	HD2
LEU96	Light	ARG5	5.65	-0.10	0	1	HD22	HA
SER28	Light	ARG5	5.69	-0.25	0	1	HB3	HH11
SER91	Light	ARG5	5.79	0.04	0	1	O	HA
PRO95	Light	PHE4	5.85	-0.15	0	0	N	H
GLN27	Light	ARG5	6.04	-0.01	0	1	HB2	HH21
GLN90	Light	HIS6	6.10	-0.17	0	0	OE1	HD2
TYR32	Light	PHE4	6.21	-0.10	0	0	HE1	HE1
THR94	Light	HIS6	6.24	-0.08	0	0	H	H

ASP1	Light	GLU3	6.26	0.26	0	-1	OD1	H
TYR92	Light	ALA2	6.35	-0.05	0	1	O	HB2
ILE29	Light	HIS6	6.43	-0.13	0	0	O	HD2
GLN90	Light	ALA2	6.57	-0.03	0	1	HE22	HB3
LEU96	Light	ALA2	6.62	-0.07	0	1	HD21	HB3
SER30	Light	ASP7	6.64	-0.23	0	-2	HG	O
SER30	Light	ARG5	6.81	0.01	0	1	HB2	HH11
ASN34	Light	HIS6	6.92	-0.01	0	0	HD22	HE2
GLN90	Light	PHE4	6.95	-0.06	0	0	HE22	O
GLN90	Light	ARG5	6.98	-0.08	0	1	OE1	HA
SER31	Light	HIS6	7.11	-0.13	0	0	H	HB3
ILE29	Light	ARG5	7.16	-0.08	0	1	HA	HB3
ASP1	Light	ARG5	7.39	0.03	0	1	HA	HH22
TYR32	Light	ARG5	7.53	-0.22	0	1	HH	C
LEU96	Light	GLU3	7.62	-0.05	0	-1	HG	HB3
ILE2	Light	HIS6	7.74	-0.06	0	0	HD11	H
ILE2	Light	PHE4	7.78	-0.03	0	0	HG13	O
SER93	Light	ASP7	7.90	0.08	0	-2	HB3	N

Table S21 – Quantum biochemistry details of RC MD6-#0

Adu Residue	Adu Chain	Aβ Residue	Distance (Å)	Interaction Energy (kcal/mol)	Adu Residue Charge	Aβ Residue Charge	Adu Atom	Aβ Atom
TYR59	Heavy	GLU3	1.78	-12.99	0	-1	HH	OE1
LYS57	Heavy	GLU3	1.89	-10.61	1	-1	HZ2	OE1
ILE102	Heavy	HIS6	2.01	-0.25	0	0	O	HE1
PRO108	Heavy	HIS6	2.04	-5.33	0	0	O	HE2
LYS57	Heavy	PHE4	2.44	-3.79	1	0	HE3	H
TYR59	Heavy	PHE4	2.46	-1.62	0	0	OH	H
ARG105	Heavy	PHE4	2.56	-6.43	1	0	HG3	HB2
GLY107	Heavy	HIS6	2.90	-2.31	0	0	O	HE2
TRP52	Heavy	PHE4	3.23	-3.02	0	0	HZ2	HD2
GLY103	Heavy	HIS6	3.39	-1.04	0	0	HA3	HE1
ARG105	Heavy	ARG5	3.51	0.57	1	1	HG3	H
LYS57	Heavy	ALA2	3.56	1.86	1	1	HZ1	O
TYR109	Heavy	HIS6	3.86	-2.01	0	0	HB3	HE2
THR56	Heavy	PHE4	3.94	-0.41	0	0	OG1	HE2
ALA104	Heavy	ASP7	4.33	-0.73	0	-2	H	OD1
ARG106	Heavy	HIS6	4.50	0.02	1	0	HA	HE1
ARG105	Heavy	ALA2	4.68	1.17	1	1	HH11	H2
TRP52	Heavy	ARG5	4.81	-0.21	0	1	HH2	HA
GLY107	Heavy	ARG5	4.90	-0.13	0	1	HA3	HA
GLY103	Heavy	ASP7	4.91	-0.58	0	-2	HA2	OD1
ASP54	Heavy	PHE4	4.95	-0.26	-1	0	OD2	HE2
GLY101	Heavy	HIS6	4.97	0.05	0	0	HA2	HE2
PRO108	Heavy	ARG5	5.14	-0.38	0	1	HD3	HA
PHE53	Heavy	PHE4	5.35	-0.26	0	0	HE1	HD2

GLY107	Heavy	PHE4	5.38	-0.17	0	0	HA3	HB2
ARG105	Heavy	HIS6	5.41	-0.43	1	0	HB3	HA
TYR59	Heavy	ALA2	5.48	0.04	0	1	HH	O
TYR59	Heavy	ARG5	5.69	0.03	0	1	OH	N
ARG105	Heavy	GLU3	5.74	-1.30	1	-1	HG3	C
ALA104	Heavy	HIS6	5.81	-0.19	0	0	H	HA
LYS58	Heavy	GLU3	5.83	-1.11	1	-1	H	OE1
PRO108	Heavy	PHE4	5.91	-0.13	0	0	HD3	O
TRP52	Heavy	HIS6	5.94	-0.18	0	0	HH2	NE2
ARG105	Heavy	ASP7	5.98	-2.40	1	-2	HG2	OD2
ARG106	Heavy	PHE4	6.06	-0.19	1	0	N	HB2
GLY103	Heavy	ARG5	6.25	-0.09	0	1	HA3	O
ASP99	Heavy	HIS6	6.30	-0.24	-1	0	OD1	HE2
LYS57	Heavy	ARG5	6.39	0.92	1	1	HE3	N
ALA104	Heavy	ARG5	6.57	0.03	0	1	H	O
ALA104	Heavy	PHE4	6.63	0.09	0	0	C	HB2
ILE102	Heavy	ASP7	6.82	-0.08	0	-2	HG21	H
TYR110	Heavy	HIS6	6.90	-0.08	0	0	H	HE2
GLY107	Heavy	ASP7	7.18	0.02	0	-2	H	H
ILE102	Heavy	ARG5	7.38	-0.18	0	1	O	O
HIS35	Heavy	HIS6	7.48	0.00	0	0	HE2	HE2
GLY103	Heavy	PHE4	7.50	0.01	0	0	HA3	HB2
ARG106	Heavy	ARG5	7.52	0.57	1	1	N	H
TRP52	Heavy	GLU3	7.52	-0.14	0	-1	HZ2	C
THR56	Heavy	GLU3	7.54	-0.07	0	-1	O	OE1
LYS58	Heavy	PHE4	7.71	-0.19	1	0	H	H
TYR59	Heavy	HIS6	7.81	-0.04	0	0	HE2	H
ARG106	Heavy	ASP7	7.90	-1.55	1	-2	H	OD1
VAL50	Heavy	PHE4	7.97	-0.02	0	0	HG11	O
TYR32	Light	ASP7	1.68	-7.70	0	-2	HH	OXT
TYR91	Light	HIS6	2.38	-1.72	0	0	O	HD2
TYR32	Light	HIS6	2.54	-5.76	0	0	CZ	HB2
TYR92	Light	HIS6	3.09	-0.50	0	0	HA	HB3
TYR92	Light	ARG5	3.35	-0.84	0	1	O	HB3
SER93	Light	ARG5	3.66	-0.55	0	1	HA	HB3
SER30	Light	ASP7	4.32	-0.48	0	-2	HG	OXT
SER93	Light	HIS6	4.80	-0.15	0	0	HA	H
THR94	Light	ARG5	4.84	-0.33	0	1	H	HB2
LEU96	Light	HIS6	5.06	-0.12	0	0	HD22	HD2
TYR92	Light	ASP7	5.11	0.24	0	-2	HE1	O
TYR91	Light	ARG5	5.56	-0.38	0	1	O	HB3
THR94	Light	PHE4	5.62	-0.09	0	0	H	O
SER30	Light	HIS6	5.89	-0.08	0	0	HB3	HB2
SER93	Light	PHE4	5.98	-0.12	0	0	HA	O
THR94	Light	HIS6	6.12	-0.08	0	0	H	H
THR94	Light	GLU3	6.53	0.02	0	-1	OG1	HB2
GLN90	Light	HIS6	6.54	-0.11	0	0	C	HD2
SER31	Light	ASP7	6.67	-0.25	0	-2	H	OXT

TYR32	Light	ARG5	6.73	-0.53	0	1	HH	O
ILE29	Light	HIS6	6.73	-0.09	0	0	O	HB3
TYR92	Light	PHE4	6.86	-0.05	0	0	O	O
LEU96	Light	ARG5	6.91	-0.03	0	1	HD21	HA
ILE29	Light	ASP7	7.24	0.05	0	-2	O	OXT
TYR91	Light	ASP7	7.32	0.22	0	-2	O	N
SER31	Light	HIS6	7.33	-0.13	0	0	H	HB2
LEU96	Light	PHE4	7.44	-0.02	0	0	HD21	O
LEU33	Light	HIS6	7.78	-0.07	0	0	N	HD2
SER28	Light	ASP7	7.87	0.24	0	-2	O	O

Table S22 – Quantum biochemistry details of RC MD6-#1

Adu Residue	Adu Chain	Aβ Residue	Distance (Å)	Interaction Energy (kcal/mol)	Adu Residue Charge	Aβ Residue Charge	Adu Atom	Aβ Atom
ARG105	Heavy	ASP7	1.73	-17.43	1	-2	HH11	OD2
ARG105	Heavy	ARG5	1.76	-2.42	1	1	HH12	O
PRO108	Heavy	HIS6	1.88	-4.49	0	0	O	HE2
LYS57	Heavy	PHE4	2.31	-2.45	1	0	HD2	HZ
ARG105	Heavy	PHE4	2.33	-1.74	1	0	HG2	HE2
TRP52	Heavy	PHE4	2.40	-2.25	0	0	HH2	HE1
ARG105	Heavy	HIS6	2.64	-4.01	1	0	HH12	HA
GLY107	Heavy	HIS6	2.67	-0.97	0	0	H	HE1
TYR59	Heavy	PHE4	2.78	-1.60	0	0	HE2	HE1
ILE102	Heavy	HIS6	2.92	-1.77	0	0	HG22	HE2
ALA104	Heavy	ASP7	3.22	-1.31	0	-2	HB2	OD1
GLY103	Heavy	HIS6	3.68	-0.35	0	0	HA3	ND1
TYR109	Heavy	HIS6	4.08	-1.51	0	0	HA	HE2
TRP52	Heavy	HIS6	4.15	-0.39	0	0	HZ2	HE1
ARG106	Heavy	HIS6	4.50	0.26	1	0	C	HE1
ALA104	Heavy	HIS6	4.50	-0.46	0	0	H	HA
GLY103	Heavy	ASP7	4.71	-0.65	0	-2	HA3	H
GLY107	Heavy	PHE4	5.04	-0.10	0	0	HA3	HE1
GLY101	Heavy	HIS6	5.06	-0.30	0	0	HA2	HE1
PRO108	Heavy	PHE4	5.15	-0.11	0	0	HD3	HE1
TRP52	Heavy	ARG5	5.49	-0.12	0	1	HH2	HA
ARG105	Heavy	GLU3	6.05	-0.92	1	-1	HH12	O
ARG106	Heavy	PHE4	6.34	-0.10	1	0	N	HZ
PRO108	Heavy	ARG5	6.45	-0.31	0	1	HD3	HA
ALA104	Heavy	ARG5	6.49	-0.14	0	1	H	O
ASP99	Heavy	HIS6	6.49	-0.24	-1	0	OD1	HE2
TYR59	Heavy	GLU3	6.49	-0.18	0	-1	HH	HB3
ILE102	Heavy	ASP7	6.52	-0.18	0	-2	HG23	H
TYR59	Heavy	ARG5	6.66	-0.09	0	1	HH	N
VAL50	Heavy	PHE4	6.73	-0.03	0	0	HG11	HE1
GLY107	Heavy	ARG5	6.80	-0.05	0	1	HA3	HA
GLY103	Heavy	ARG5	6.84	0.02	0	1	HA3	C

TYR110	Heavy	HIS6	7.00	-0.09	0	0	N	HE2
LYS57	Heavy	ARG5	7.06	0.59	1	1	HE2	H
ALA104	Heavy	PHE4	7.19	0.04	0	0	C	HZ
PHE53	Heavy	PHE4	7.29	-0.04	0	0	HE1	HZ
ARG106	Heavy	ASP7	7.40	-1.36	1	-2	H	H
LYS58	Heavy	PHE4	7.40	-0.10	1	0	N	HE1
THR56	Heavy	PHE4	7.47	-0.07	0	0	OG1	HZ
HIS35	Heavy	HIS6	7.58	0.00	0	0	HE2	HE2
GLY107	Heavy	ASP7	7.97	0.07	0	-2	H	H
ARG100	Heavy	HIS6	7.98	0.02	1	0	C	HE1
TYR32	Light	ASP7	1.61	-7.00	0	-2	HH	O
SER93	Light	GLU3	2.06	-7.62	0	-1	HG	OE1
TYR92	Light	HIS6	2.44	-1.06	0	0	HE1	O
TYR32	Light	HIS6	2.61	-5.76	0	0	HD2	HD2
TYR92	Light	ARG5	2.71	-6.39	0	1	O	HB3
THR94	Light	GLU3	2.81	-1.24	0	-1	H	OE1
TYR91	Light	HIS6	3.13	-0.76	0	0	HB2	HE2
TYR92	Light	ASP7	3.35	0.36	0	-2	HE1	O
SER93	Light	ARG5	3.55	-0.17	0	1	HA	HB2
THR94	Light	PHE4	4.16	-0.58	0	0	HG1	O
ASP1	Light	GLU3	4.23	1.29	0	-1	OD1	OE2
TYR92	Light	GLU3	4.42	-0.37	0	-1	O	OE1
SER93	Light	PHE4	4.44	-0.61	0	0	HA	O
TYR91	Light	ARG5	4.54	-0.43	0	1	O	HB3
THR94	Light	ARG5	4.68	-0.38	0	1	HG1	HA
TYR92	Light	PHE4	4.71	-0.40	0	0	O	O
ILE2	Light	ARG5	4.99	-0.05	0	1	HD12	HH22
SER30	Light	ASP7	5.25	-0.53	0	-2	HG	O
TYR32	Light	ARG5	5.40	-0.51	0	1	HE1	HG3
ILE2	Light	GLU3	5.46	-0.15	0	-1	HG13	OE1
SER28	Light	ARG5	5.69	-0.27	0	1	O	HH21
PRO95	Light	GLU3	5.84	0.52	0	-1	HD3	OE1
LEU96	Light	HIS6	5.89	-0.08	0	0	HD21	HE2
SER93	Light	HIS6	6.01	-0.05	0	0	N	H
SER30	Light	HIS6	6.10	-0.04	0	0	HB3	HB3
GLN27	Light	ARG5	6.20	0.15	0	1	HB3	HH21
ILE29	Light	ARG5	6.27	-0.18	0	1	HA	HD2
TYR92	Light	ALA2	6.27	-0.26	0	1	HH	HB1
THR94	Light	HIS6	6.41	-0.07	0	0	HG1	H
GLN27	Light	GLU3	6.49	-0.23	0	-1	HE22	OE2
SER31	Light	HIS6	6.51	-0.09	0	0	C	HD2
ILE29	Light	HIS6	6.52	-0.10	0	0	O	HD2
ASP1	Light	ARG5	6.55	-0.31	0	1	OD1	HH22
GLN90	Light	GLU3	6.62	0.02	0	-1	HE22	OE1
LEU33	Light	HIS6	6.62	-0.10	0	0	N	HD2
SER31	Light	ASP7	6.70	-0.34	0	-2	H	O
LEU96	Light	ARG5	6.87	-0.03	0	1	HD23	HA
TYR91	Light	PHE4	6.93	-0.08	0	0	O	O

ILE29	Light	ASP7	7.03	0.08	0	-2	O	O
LEU96	Light	PHE4	7.11	-0.05	0	0	HD23	HD1
LEU96	Light	GLU3	7.39	-0.02	0	-1	HD23	OE1
GLN90	Light	ARG5	7.39	-0.14	0	1	OE1	HB3
GLN90	Light	HIS6	7.45	-0.06	0	0	C	HD2
SER30	Light	ARG5	7.61	-0.01	0	1	HB2	HD2
SER93	Light	ALA2	7.76	0.03	0	1	HG	C
ALA50	Light	HIS6	7.79	-0.01	0	0	HA	HD2
GLN27	Light	ALA2	7.86	0.13	0	1	HE21	HA
ASP1	Light	ALA2	7.89	-0.36	0	1	OD1	HA

Table S23 – Quantum biochemistry details of RC MD6-#2

Adu Residue	Adu Chain	Aβ Residue	Distance (Å)	Interaction Energy (kcal/mol)	Adu Residue Charge	Aβ Residue Charge	Adu Atom	Aβ Atom
PRO108	Heavy	HIS6	1.81	-5.37	0	0	O	HE2
ARG105	Heavy	ASP7	1.94	-15.44	1	-2	HH21	OD2
ARG105	Heavy	ARG5	2.11	-2.11	1	1	HH12	O
ARG105	Heavy	PHE4	2.18	-1.94	1	0	HD2	HZ
LYS57	Heavy	PHE4	2.21	-1.76	1	0	HE2	HZ
ILE102	Heavy	HIS6	2.28	-1.45	0	0	HG22	HE1
GLY107	Heavy	HIS6	2.57	-2.18	0	0	O	HE1
ARG105	Heavy	HIS6	2.76	-3.23	1	0	HH12	HA
TYR59	Heavy	PHE4	2.85	-2.22	0	0	HE2	HE2
TRP52	Heavy	PHE4	3.01	-2.21	0	0	HH2	HE2
TYR109	Heavy	HIS6	3.78	-1.87	0	0	HA	HE2
ARG106	Heavy	HIS6	4.26	-0.17	1	0	C	HE1
TRP52	Heavy	ARG5	4.51	-0.20	0	1	HH2	HA
GLY103	Heavy	HIS6	4.84	-0.23	0	0	N	HE1
GLY101	Heavy	HIS6	4.89	-0.05	0	0	HA2	HE1
ALA104	Heavy	ASP7	4.97	-0.92	0	-2	HB1	OD2
GLY103	Heavy	ASP7	5.48	-0.47	0	-2	HA3	HB3
TRP52	Heavy	HIS6	5.50	-0.37	0	0	HH2	H
GLY107	Heavy	ARG5	5.94	-0.05	0	1	HA3	HA
GLY107	Heavy	PHE4	5.96	-0.11	0	0	HA3	HE2
PRO108	Heavy	ARG5	5.98	-0.35	0	1	HD3	HA
ILE102	Heavy	ASP7	6.32	-0.09	0	-2	HG22	H
ASP99	Heavy	HIS6	6.38	-0.24	-1	0	OD1	HE2
PRO108	Heavy	PHE4	6.43	-0.08	0	0	HD2	HE2
TYR59	Heavy	ARG5	6.63	-0.13	0	1	HE2	HA
ARG106	Heavy	PHE4	6.68	-0.09	1	0	N	HZ
ALA104	Heavy	HIS6	6.72	-0.12	0	0	N	HE1
GLY107	Heavy	ASP7	6.74	-0.01	0	-2	H	H
TYR110	Heavy	HIS6	6.87	-0.13	0	0	N	HE2
THR56	Heavy	PHE4	6.95	-0.12	0	0	OG1	HZ
ASP54	Heavy	PHE4	7.02	-0.04	-1	0	OD2	HZ
ARG106	Heavy	ASP7	7.06	-1.56	1	-2	H	OD2

LYS58	Heavy	PHE4	7.20	-0.12	1	0	H	HE2
HIS35	Heavy	HIS6	7.23	0.00	0	0	HE1	HE2
ALA104	Heavy	PHE4	7.34	0.06	0	0	C	HZ
VAL50	Heavy	PHE4	7.36	-0.03	0	0	HG13	HE2
ARG105	Heavy	GLU3	7.39	-0.88	1	-1	HH12	O
TYR59	Heavy	ALA2	7.46	-0.02	0	1	HH	O
ARG100	Heavy	HIS6	7.52	0.05	1	0	HH22	HE1
PHE53	Heavy	HIS6	7.65	-0.05	0	0	HB2	HE1
TYR59	Heavy	GLU3	7.67	-0.11	0	-1	HH	C
LYS57	Heavy	ARG5	7.69	0.63	1	1	HE2	N
TRP52	Heavy	ASP7	7.82	-0.05	0	-2	HZ2	H
SER93	Light	GLU3	1.81	-8.25	0	-1	HG	OE1
TYR32	Light	ASP7	1.93	-7.06	0	-2	HH	OXT
TYR91	Light	HIS6	2.31	-1.68	0	0	O	HD2
TYR32	Light	HIS6	2.46	-6.58	0	0	HH	HB2
TYR92	Light	ARG5	2.57	-6.84	0	1	O	HB3
TYR92	Light	HIS6	2.69	-1.55	0	0	HD1	HB3
SER93	Light	ARG5	3.41	-0.78	0	1	HA	HB2
THR94	Light	GLU3	3.52	-0.47	0	-1	H	OE1
TYR92	Light	GLU3	3.89	0.24	0	-1	O	OE2
TYR92	Light	ASP7	4.01	-0.32	0	-2	HE1	HA
THR94	Light	PHE4	4.04	-0.37	0	0	HG1	O
TYR91	Light	ARG5	4.39	-0.31	0	1	O	HB3
SER30	Light	ASP7	4.52	-0.67	0	-2	HG	OXT
SER93	Light	PHE4	4.91	-0.45	0	0	HA	O
ILE2	Light	GLU3	5.01	-0.29	0	-1	HG13	OE1
ASP1	Light	GLU3	5.09	-0.68	0	-1	H2	OE1
SER30	Light	HIS6	5.09	-0.15	0	0	HB3	HB3
THR94	Light	ARG5	5.17	-0.44	0	1	H	HB2
TYR92	Light	PHE4	5.54	-0.62	0	0	O	O
ILE29	Light	ARG5	5.66	-0.12	0	1	HA	HD2
LEU96	Light	HIS6	5.78	-0.10	0	0	HD21	HD2
SER28	Light	ARG5	5.81	-0.30	0	1	O	HD2
ILE29	Light	HIS6	5.86	-0.16	0	0	O	HB3
SER93	Light	HIS6	5.93	-0.09	0	0	N	H
ILE2	Light	ARG5	6.02	-0.06	0	1	HD12	HD3
TYR32	Light	ARG5	6.09	-0.53	0	1	HD1	HB3
SER31	Light	HIS6	6.14	-0.23	0	0	H	HB3
SER31	Light	ASP7	6.16	-0.32	0	-2	H	OXT
LEU96	Light	ARG5	6.18	-0.06	0	1	HD23	HA
LEU96	Light	PHE4	6.40	-0.06	0	0	HD23	O
SER30	Light	ARG5	6.54	-0.01	0	1	HB3	HD2
GLN90	Light	GLU3	6.57	-0.02	0	-1	HE22	OE1
GLN90	Light	ARG5	6.62	-0.12	0	1	OE1	HB3
GLN90	Light	HIS6	6.62	-0.09	0	0	C	HD2
GLN27	Light	GLU3	6.67	-0.10	0	-1	HB2	OE2
TYR91	Light	PHE4	6.75	-0.04	0	0	O	O
PRO95	Light	GLU3	6.98	0.26	0	-1	N	OE1

SER28	Light	GLU3	7.04	0.23	0	-1	O	OE2
LEU33	Light	HIS6	7.15	-0.08	0	0	N	HD2
THR94	Light	ALA2	7.27	-0.09	0	1	HB	O
GLN27	Light	ARG5	7.44	-0.03	0	1	HB2	HD3
ASN34	Light	HIS6	7.56	-0.03	0	0	HD22	HE2
LEU96	Light	GLU3	7.61	-0.03	0	-1	HD22	HG3
TYR91	Light	ASP7	7.63	0.21	0	-2	O	N
THR94	Light	HIS6	7.66	-0.03	0	0	OG1	H
ILE29	Light	GLU3	7.77	-0.01	0	-1	HG22	OE2
ILE29	Light	ASP7	7.82	-0.04	0	-2	O	OXT

Table S24 – Quantum biochemistry details of RC MD6-#3

Adu Residue	Adu Chain	Aβ Residue	Distance (Å)	Interaction Energy (kcal/mol)	Adu Residue Charge	Aβ Residue Charge	Adu Atom	Aβ Atom
TYR59	Heavy	GLU3	1.63	-13.48	0	-1	HH	OE2
PRO108	Heavy	HIS6	1.91	-4.92	0	0	O	HE2
LYS57	Heavy	GLU3	1.95	-11.90	1	-1	HZ1	OE1
TYR59	Heavy	PHE4	2.15	-3.62	0	0	OH	H
GLY107	Heavy	HIS6	2.19	-1.54	0	0	O	HE1
ARG105	Heavy	PHE4	2.36	-6.07	1	0	HB2	HB2
LYS57	Heavy	PHE4	2.43	-3.85	1	0	HG3	HD2
ILE102	Heavy	HIS6	2.47	-1.24	0	0	HG22	HE1
TRP52	Heavy	PHE4	2.73	-4.36	0	0	HZ2	HD2
ARG105	Heavy	ARG5	2.76	-0.08	1	1	HG3	H
LYS57	Heavy	ALA2	2.82	0.40	1	1	HE3	O
ARG105	Heavy	ALA2	3.45	0.79	1	1	HH11	HB3
THR56	Heavy	PHE4	3.68	-0.51	0	0	OG1	HE2
ARG106	Heavy	HIS6	3.69	-0.85	1	0	C	HE1
ALA104	Heavy	ASP7	3.71	0.09	0	-2	H	OD2
TYR109	Heavy	HIS6	3.83	-1.82	0	0	HA	HE2
ARG105	Heavy	HIS6	3.92	-0.81	1	0	HB3	HA
ARG105	Heavy	ASP7	3.99	-3.28	1	-2	HG2	OD1
GLY103	Heavy	HIS6	4.02	-0.13	0	0	HA3	HE1
TYR59	Heavy	ALA2	4.60	-0.42	0	1	HH	O
GLY101	Heavy	HIS6	4.61	-0.08	0	0	HA2	HE1
GLY103	Heavy	ASP7	4.70	-0.63	0	-2	HA2	OD2
TRP52	Heavy	ARG5	4.86	-0.22	0	1	HH2	HA
GLY107	Heavy	PHE4	4.95	-0.26	0	0	H	HB2
ILE102	Heavy	ASP7	5.41	-0.23	0	-2	HG23	H
ASP54	Heavy	PHE4	5.42	-0.19	-1	0	OD2	HE2
GLY107	Heavy	ARG5	5.44	-0.13	0	1	HA3	HA
TRP52	Heavy	HIS6	5.51	-0.26	0	0	HZ2	HE1
ARG105	Heavy	GLU3	5.58	-1.42	1	-1	HG3	O
PRO108	Heavy	ARG5	5.79	-0.25	0	1	HD3	HA
ARG106	Heavy	PHE4	5.82	-0.02	1	0	N	HB2
LYS58	Heavy	GLU3	5.83	-1.07	1	-1	O	OE2

TYR59	Heavy	ARG5	6.30	-0.07	0	1	HE2	N
PRO108	Heavy	PHE4	6.33	-0.13	0	0	HD2	HB3
ALA104	Heavy	HIS6	6.40	-0.17	0	0	N	HE1
ALA104	Heavy	PHE4	6.40	-0.04	0	0	O	HZ
GLY107	Heavy	ASP7	6.56	0.07	0	-2	H	H
TRP52	Heavy	GLU3	6.79	-0.19	0	-1	HZ2	C
ASP99	Heavy	HIS6	6.80	-0.18	-1	0	OD1	HE2
TYR110	Heavy	HIS6	6.90	-0.07	0	0	N	HE2
ALA104	Heavy	ARG5	6.97	-0.03	0	1	HB3	O
GLY103	Heavy	ARG5	7.02	-0.03	0	1	HA3	O
ARG106	Heavy	ASP7	7.07	-1.62	1	-2	H	OD1
LYS58	Heavy	PHE4	7.18	-0.23	1	0	N	HD2
PHE53	Heavy	PHE4	7.18	-0.08	0	0	HB2	HE2
ARG106	Heavy	ARG5	7.20	0.45	1	1	H	O
VAL50	Heavy	PHE4	7.30	-0.03	0	0	HG12	HD2
LYS57	Heavy	ARG5	7.31	0.75	1	1	HG3	N
ARG100	Heavy	HIS6	7.37	0.03	1	0	O	HE1
LYS65	Heavy	GLU3	7.46	-1.10	1	-1	HZ3	HG3
HIS35	Heavy	HIS6	7.76	-0.01	0	0	HE1	HE2
ILE102	Heavy	ARG5	7.88	-0.04	0	1	O	O
GLY55	Heavy	PHE4	7.92	-0.01	0	0	C	HE2
ILE102	Heavy	PHE4	7.92	-0.03	0	0	O	HB2
TYR60	Heavy	GLU3	7.97	0.05	0	-1	H	OE2
TYR32	Light	ASP7	1.96	-9.41	0	-2	HH	O
TYR92	Light	HIS6	2.27	-5.08	0	0	O	H
TYR32	Light	HIS6	2.50	-5.10	0	0	HB2	HD2
TYR91	Light	HIS6	2.83	-1.67	0	0	O	HD2
TYR92	Light	ARG5	2.96	-3.82	0	1	O	HB3
SER93	Light	ARG5	3.08	-2.06	0	1	HG	HB2
TYR92	Light	ASP7	3.13	0.31	0	-2	HE1	OXT
THR94	Light	PHE4	3.39	-0.34	0	0	HG23	O
SER93	Light	HIS6	3.72	-1.27	0	0	HA	H
THR94	Light	ARG5	4.36	-0.59	0	1	H	HA
SER30	Light	ASP7	4.49	-0.39	0	-2	HB2	O
SER93	Light	PHE4	4.55	-0.34	0	0	HA	O
SER30	Light	HIS6	5.37	-0.15	0	0	HB3	HB2
TYR91	Light	ARG5	5.38	-0.28	0	1	O	HA
THR94	Light	HIS6	5.46	-0.15	0	0	H	H
TYR92	Light	PHE4	5.65	-0.22	0	0	O	O
THR94	Light	GLU3	5.66	-0.06	0	-1	HG22	HB2
LEU96	Light	HIS6	6.20	-0.10	0	0	HD22	HD2
GLN90	Light	HIS6	6.21	-0.08	0	0	C	HD2
ILE29	Light	HIS6	6.22	-0.16	0	0	O	HB3
GLN27	Light	ARG5	6.37	0.04	0	1	HE22	HH21
LEU33	Light	HIS6	6.37	-0.13	0	0	N	HD2
SER31	Light	ASP7	6.58	-0.27	0	-2	H	O
ILE2	Light	ARG5	6.65	-0.06	0	1	HD13	HB3
SER31	Light	HIS6	6.70	-0.10	0	0	C	HD2

LEU96	Light	ARG5	6.74	-0.03	0	1	HD21	HA
ASP1	Light	ARG5	6.75	0.19	0	1	H2	HH11
SER93	Light	GLU3	6.88	-0.10	0	-1	HG	O
TYR32	Light	ARG5	6.96	-0.33	0	1	HH	O
TYR91	Light	PHE4	7.00	-0.04	0	0	O	O
LEU96	Light	PHE4	7.05	-0.03	0	0	HD21	O
ILE2	Light	HIS6	7.14	-0.04	0	0	HD12	HB3
ILE29	Light	ASP7	7.21	-0.12	0	-2	O	O
TYR91	Light	ASP7	7.45	0.12	0	-2	O	N

Table S25 – Quantum biochemistry details of RC MD6-#4

Adu Residue	Adu Chain	Aβ Residue	Distance (Å)	Interaction Energy (kcal/mol)	Adu Residue Charge	Aβ Residue Charge	Adu Atom	Aβ Atom
PRO108	Heavy	HIS6	1.73	-3.62	0	0	O	HE2
ARG105	Heavy	ASP7	1.94	-15.85	1	-2	HE	OD1
GLY107	Heavy	HIS6	2.42	-1.45	0	0	O	HE1
TYR59	Heavy	ALA2	2.43	-0.59	0	1	HE1	HB2
TYR59	Heavy	PHE4	2.45	-4.46	0	0	CE2	HB3
ILE102	Heavy	HIS6	2.45	-1.77	0	0	HG22	HE1
ARG105	Heavy	ARG5	2.63	0.08	1	1	HH11	O
GLY107	Heavy	PHE4	2.73	-1.29	0	0	HA3	CZ
ARG105	Heavy	PHE4	2.74	-2.85	1	0	HD2	HE2
TRP52	Heavy	PHE4	2.81	-3.48	0	0	HH2	CD2
PRO108	Heavy	PHE4	3.00	-1.97	0	0	HD3	HD1
TYR59	Heavy	GLU3	3.08	-2.66	0	-1	HD1	HA
TYR109	Heavy	HIS6	3.48	-2.28	0	0	HA	HE2
LYS57	Heavy	PHE4	3.62	-0.57	1	0	HD3	HD2
GLY103	Heavy	ASP7	3.64	-1.05	0	-2	HA3	HB3
ALA104	Heavy	ASP7	3.93	-1.10	0	-2	H	HB3
ARG105	Heavy	HIS6	4.07	-0.64	1	0	HH12	HA
LYS65	Heavy	ALA2	4.23	1.25	1	1	HZ2	O
LYS65	Heavy	GLU3	4.35	-1.82	1	-1	HZ2	HG2
ARG106	Heavy	PHE4	4.52	-0.08	1	0	N	HZ
ARG105	Heavy	GLU3	4.55	-0.92	1	-1	HH21	O
ARG106	Heavy	HIS6	4.56	-0.25	1	0	C	HE1
GLY103	Heavy	HIS6	4.66	-0.23	0	0	HA3	HE1
ILE102	Heavy	PHE4	4.68	-0.26	0	0	O	HZ
GLY103	Heavy	PHE4	4.75	-0.11	0	0	HA3	HZ
GLY101	Heavy	HIS6	4.79	-0.06	0	0	HA2	HE1
VAL50	Heavy	PHE4	5.16	-0.22	0	0	HG13	HB2
ASP62	Heavy	GLU3	5.34	1.56	-1	-1	OD1	HG2
LYS58	Heavy	ALA2	5.41	0.61	1	1	O	H
ILE102	Heavy	ASP7	5.56	-0.26	0	-2	HG23	H
TYR59	Heavy	ARG5	5.59	-0.20	0	1	HH	H
TYR60	Heavy	ALA2	5.67	-0.27	0	1	H	O
ARG106	Heavy	ASP7	5.68	-1.71	1	-2	H	OD1

TRP47	Heavy	PHE4	5.75	-0.11	0	0	HH2	HB2
TYR60	Heavy	GLU3	5.80	0.00	0	-1	O	HG2
GLY107	Heavy	ASP7	5.96	-0.04	0	-2	H	H
TRP52	Heavy	HIS6	6.01	-0.12	0	0	HZ2	HE1
PRO108	Heavy	ARG5	6.10	-0.37	0	1	HD3	HA
ALA104	Heavy	PHE4	6.13	0.05	0	0	H	HZ
LYS58	Heavy	PHE4	6.24	-0.19	1	0	O	HB3
ARG105	Heavy	ALA2	6.26	1.05	1	1	HH21	HB3
GLY107	Heavy	ARG5	6.42	-0.20	0	1	HA3	O
TRP52	Heavy	ARG5	6.44	-0.10	0	1	HH2	H
TYR60	Heavy	PHE4	6.47	-0.10	0	0	H	H
TYR110	Heavy	HIS6	6.57	-0.12	0	0	N	HE2
TRP47	Heavy	GLU3	6.58	0.00	0	-1	HH2	HA
LYS57	Heavy	ALA2	6.62	1.16	1	1	HE3	HB2
ASP62	Heavy	ALA2	6.72	-1.07	-1	1	OD1	O
ASP99	Heavy	HIS6	6.74	-0.19	-1	0	OD1	HE2
LYS58	Heavy	GLU3	6.78	-0.63	1	-1	O	HA
THR61	Heavy	GLU3	7.02	-0.05	0	-1	HA	HG2
PHE53	Heavy	PHE4	7.04	-0.08	0	0	HE1	HE2
ALA104	Heavy	HIS6	7.13	-0.07	0	0	N	HE1
TRP52	Heavy	GLU3	7.14	-0.04	0	-1	HH2	C
LYS65	Heavy	PHE4	7.23	-0.11	1	0	HZ2	H
TYR109	Heavy	PHE4	7.24	-0.09	0	0	N	HE1
GLY101	Heavy	PHE4	7.25	-0.02	0	0	HA2	HE1
TRP52	Heavy	ASP7	7.40	-0.12	0	-2	HZ2	OD1
LYS57	Heavy	GLU3	7.57	-0.74	1	-1	HD3	O
TRP47	Heavy	ALA2	7.69	-0.10	0	1	HH2	O
PRO108	Heavy	ASP7	7.69	0.46	0	-2	HD3	H
HIS35	Heavy	PHE4	7.70	-0.03	0	0	HE1	HB2
GLY103	Heavy	ARG5	7.77	-0.01	0	1	HA3	O
VAL50	Heavy	GLU3	7.79	-0.01	0	-1	HG11	HA
HIS35	Heavy	HIS6	7.89	0.02	0	0	HE1	HE2
ARG100	Heavy	HIS6	7.92	0.01	1	0	C	HE1
LYS57	Heavy	ARG5	7.94	0.56	1	1	HD3	H
PRO108	Heavy	GLU3	7.97	-0.01	0	-1	HD3	C
TYR92	Light	ARG5	1.89	-11.46	0	1	OH	HH12
TYR92	Light	HIS6	2.04	-5.19	0	0	O	H
THR94	Light	PHE4	2.08	-6.24	0	0	H	O
SER93	Light	ARG5	2.21	-4.69	0	1	HA	HA
SER93	Light	PHE4	2.54	-4.35	0	0	HA	O
TYR91	Light	HIS6	2.86	-0.42	0	0	O	HD2
TYR32	Light	HIS6	2.97	-4.04	0	0	CD2	HD2
THR94	Light	GLU3	3.08	-2.46	0	-1	HG1	HA
THR94	Light	ARG5	3.52	-1.56	0	1	H	HA
PRO95	Light	GLU3	3.65	-0.29	0	-1	HD2	HG2
SER93	Light	HIS6	3.69	-1.73	0	0	HA	H
TYR32	Light	ASP7	3.87	-0.13	0	-2	HH	OXT
TYR92	Light	PHE4	4.35	-1.57	0	0	O	HD1

SER93	Light	GLU3	4.41	0.15	0	-1	HB2	HB2
THR94	Light	ALA2	4.94	-0.24	0	1	HG1	O
TYR91	Light	PHE4	5.24	-0.21	0	0	O	HD1
ILE2	Light	ARG5	5.35	-0.10	0	1	HD11	HG3
LEU96	Light	PHE4	5.39	-0.18	0	0	HD23	HD1
SER28	Light	ARG5	5.43	-0.38	0	1	O	HH12
GLN27	Light	ARG5	5.45	-0.25	0	1	OE1	HH21
THR94	Light	HIS6	5.58	-0.14	0	0	H	H
ILE29	Light	HIS6	5.70	-0.18	0	0	O	HD2
TYR91	Light	ARG5	5.71	0.04	0	1	O	HA
ASP1	Light	GLU3	5.80	-0.55	0	-1	H	OE2
TYR92	Light	ASP7	5.88	0.30	0	-2	HE1	OXT
SER30	Light	HIS6	5.94	-0.09	0	0	HB2	HB3
PRO95	Light	PHE4	6.04	0.00	0	0	N	H
ILE29	Light	ARG5	6.07	-0.15	0	1	HA	HH12
SER30	Light	ARG5	6.10	-0.15	0	1	OG	HH12
SER31	Light	HIS6	6.28	-0.20	0	0	H	HD2
LEU96	Light	HIS6	6.38	-0.08	0	0	HD21	HE2
LEU96	Light	ARG5	6.63	-0.05	0	1	HD23	HA
GLN90	Light	HIS6	6.70	-0.12	0	0	OE1	HD2
TYR32	Light	PHE4	6.78	-0.08	0	0	HE2	HE1
PRO95	Light	ALA2	6.78	-0.14	0	1	HD2	O
GLN90	Light	ARG5	6.87	-0.10	0	1	OE1	HG3
ALA50	Light	HIS6	6.89	-0.02	0	0	HB3	HE2
TYR32	Light	ARG5	7.04	-0.25	0	1	HE1	HG2
ASP1	Light	ARG5	7.21	0.36	0	1	H2	HD2
GLN90	Light	PHE4	7.22	-0.04	0	0	HE22	O
ASN34	Light	HIS6	7.25	-0.03	0	0	HD22	HE2
ILE2	Light	HIS6	7.32	-0.04	0	0	HD11	H
SER30	Light	ASP7	7.45	-0.01	0	-2	HB2	OXT
LEU33	Light	HIS6	7.48	-0.07	0	0	N	HD2
PRO95	Light	ARG5	7.48	-0.16	0	1	HD2	HB3
TYR92	Light	GLU3	7.81	-0.02	0	-1	O	HB2
ILE2	Light	PHE4	7.81	-0.02	0	0	HD11	O
ILE2	Light	GLU3	7.83	-0.01	0	-1	HG13	OE2
SER93	Light	ASP7	7.92	0.04	0	-2	HA	N
SER31	Light	ASP7	7.93	-0.10	0	-2	HG	OXT
TYR91	Light	ASP7	7.99	0.16	0	-2	O	H

Table S26 – Quantum biochemistry details of RC MD6-#5

Adu Residue	Adu Chain	Aβ Residue	Distance (Å)	Interaction Energy (kcal/mol)	Adu Residue Charge	Aβ Residue Charge	Adu Atom	Aβ Atom
ARG105	Heavy	ASP7	1.67	-16.58	1	-2	HH11	OD1
PRO108	Heavy	HIS6	1.93	-5.91	0	0	O	HE2
ARG105	Heavy	ARG5	2.35	-0.99	1	1	HH12	O
ILE102	Heavy	HIS6	2.45	-1.60	0	0	HG21	HE1

GLY107	Heavy	HIS6	2.47	-1.85	0	0	O	HE1
ARG105	Heavy	PHE4	2.49	-1.65	1	0	HB2	HZ
ARG105	Heavy	HIS6	2.59	-2.50	1	0	HH12	HA
TYR59	Heavy	PHE4	2.68	-1.42	0	0	OH	HD1
LYS57	Heavy	PHE4	2.85	-1.70	1	0	HD2	HE1
TRP52	Heavy	PHE4	3.15	-1.69	0	0	HZ2	HE1
TYR109	Heavy	HIS6	4.06	-2.01	0	0	HA	HE2
ARG106	Heavy	HIS6	4.46	-0.17	1	0	C	HE1
GLY103	Heavy	HIS6	4.58	-0.08	0	0	HA3	HE1
GLY101	Heavy	HIS6	4.92	0.01	0	0	HA2	HE1
GLY107	Heavy	PHE4	5.33	-0.13	0	0	H	HZ
TRP52	Heavy	HIS6	5.50	-0.21	0	0	HZ2	HE1
GLY103	Heavy	ASP7	5.57	-0.27	0	-2	HA3	H
ALA104	Heavy	ASP7	5.63	-0.85	0	-2	HB3	OD2
ARG106	Heavy	PHE4	5.88	-0.17	1	0	N	HZ
TRP52	Heavy	ARG5	6.02	-0.08	0	1	HH2	HA
TYR59	Heavy	GLU3	6.15	-0.13	0	-1	HH	HB3
ILE102	Heavy	ASP7	6.36	-0.04	0	-2	HG22	H
PRO108	Heavy	PHE4	6.42	-0.08	0	0	HD2	HE1
ALA104	Heavy	PHE4	6.50	0.23	0	0	C	HZ
ASP99	Heavy	HIS6	6.52	-0.23	-1	0	OD1	HE2
PRO108	Heavy	ARG5	6.58	-0.30	0	1	HD3	HA
ALA104	Heavy	HIS6	6.64	-0.18	0	0	H	HA
GLY107	Heavy	ARG5	6.75	-0.09	0	1	HA3	HA
THR56	Heavy	PHE4	6.95	-0.03	0	0	OG1	HE1
TYR110	Heavy	HIS6	7.02	-0.10	0	0	N	HE2
TYR59	Heavy	ARG5	7.14	-0.08	0	1	HH	N
HIS35	Heavy	HIS6	7.16	-0.01	0	0	HE1	HE2
ARG100	Heavy	HIS6	7.47	0.02	1	0	O	HE1
ARG106	Heavy	ASP7	7.56	-1.34	1	-2	H	H
ILE102	Heavy	PHE4	7.80	-0.02	0	0	O	HZ
GLY107	Heavy	ASP7	7.81	0.10	0	-2	H	H
LYS58	Heavy	PHE4	7.88	-0.07	1	0	H	HE1
GLY103	Heavy	ARG5	7.92	-0.04	0	1	HA3	O
GLY103	Heavy	PHE4	7.93	0.00	0	0	HA3	HZ
TYR32	Light	ASP7	1.69	-7.44	0	-2	HH	OXT
SER93	Light	GLU3	1.93	-14.64	0	-1	HG	OE2
THR94	Light	GLU3	2.36	-1.59	0	-1	H	OE1
TYR32	Light	HIS6	2.45	-5.94	0	0	HB2	HD2
TYR92	Light	ARG5	3.04	-3.51	0	1	O	HB2
TYR91	Light	HIS6	3.08	-1.52	0	0	O	HD2
TYR92	Light	HIS6	3.20	-0.67	0	0	HD1	HB3
TYR92	Light	ASP7	4.23	0.04	0	-2	HH	O
TYR92	Light	GLU3	4.29	-0.42	0	-1	O	HG3
SER93	Light	ARG5	4.39	0.11	0	1	HA	HB2
THR94	Light	PHE4	4.41	-0.24	0	0	HG1	O
ASP1	Light	GLU3	4.50	0.07	0	-1	HB2	OE2
ILE2	Light	GLU3	5.09	-0.23	0	-1	HD13	OE2

TYR91	Light	ARG5	5.12	-0.40	0	1	O	HA
TYR92	Light	PHE4	5.19	-0.29	0	0	O	O
SER93	Light	PHE4	5.24	-0.29	0	0	HA	O
THR94	Light	ARG5	5.49	-0.24	0	1	HG1	HB2
ILE2	Light	ARG5	5.59	-0.02	0	1	HD13	HH22
PRO95	Light	GLU3	5.62	1.08	0	-1	N	OE1
SER30	Light	ASP7	5.64	-0.14	0	-2	HB3	OXT
SER30	Light	HIS6	5.65	-0.10	0	0	HB3	HB3
LEU96	Light	HIS6	5.85	-0.10	0	0	HD22	HE2
SER93	Light	HIS6	6.04	-0.12	0	0	H	HD2
SER31	Light	ASP7	6.17	-0.25	0	-2	HB2	OXT
ILE29	Light	HIS6	6.18	-0.13	0	0	O	HB3
TYR32	Light	ARG5	6.24	-0.44	0	1	CD1	HB3
SER31	Light	HIS6	6.26	-0.25	0	0	H	HB3
LEU33	Light	HIS6	6.31	-0.15	0	0	N	HD2
TYR91	Light	PHE4	6.36	-0.04	0	0	O	O
ILE29	Light	ARG5	6.37	-0.11	0	1	HA	HD2
SER28	Light	ARG5	6.46	-0.24	0	1	O	HD2
GLN90	Light	HIS6	6.54	-0.06	0	0	C	HD2
ASP1	Light	ARG5	6.66	-0.32	0	1	OD2	HH21
GLN27	Light	ARG5	6.69	-0.11	0	1	OE1	HH21
GLN90	Light	GLU3	6.74	0.01	0	-1	HE22	OE2
LEU96	Light	GLU3	6.75	-0.03	0	-1	HD23	OE1
THR94	Light	HIS6	6.90	-0.05	0	0	HG1	H
LEU96	Light	PHE4	7.06	-0.03	0	0	HD21	O
GLN27	Light	GLU3	7.16	0.03	0	-1	HG3	OE2
LEU96	Light	ARG5	7.17	-0.02	0	1	HD21	HA
SER30	Light	ARG5	7.25	-0.04	0	1	HB3	HB3
TYR91	Light	GLU3	7.34	-0.13	0	-1	O	HG3
ILE29	Light	ASP7	7.76	0.06	0	-2	O	OXT
GLN90	Light	ARG5	7.76	-0.09	0	1	OE1	HB2
ASN34	Light	HIS6	7.81	-0.04	0	0	HD22	HE2
ILE29	Light	GLU3	7.84	0.01	0	-1	HG23	OE2
TYR92	Light	ALA2	7.85	-0.23	0	1	HE2	HB3
SER93	Light	ALA2	7.89	0.23	0	1	HG	HB1

Table S27 – Quantum biochemistry details of RC MD6-#6

Adu Residue	Adu Chain	Aβ Residue	Distance (Å)	Interaction Energy (kcal/mol)	Adu Residue Charge	Aβ Residue Charge	Adu Atom	Aβ Atom
TYR59	Heavy	GLU3	1.49	-12.11	0	-1	HH	OE1
LYS57	Heavy	GLU3	1.65	-13.21	1	-1	HZ2	OE1
PRO108	Heavy	HIS6	1.99	-5.64	0	0	O	HE2
ILE102	Heavy	HIS6	2.31	-2.41	0	0	O	HE1
TYR59	Heavy	PHE4	2.38	-3.06	0	0	OH	H
LYS57	Heavy	PHE4	2.55	-3.48	1	0	HG3	HE2
ARG105	Heavy	PHE4	2.80	-4.70	1	0	HD2	CZ

TRP52	Heavy	PHE4	2.83	-5.04	0	0	CE2	HD2
GLY107	Heavy	HIS6	2.86	-2.02	0	0	H	HE1
LYS57	Heavy	ALA2	3.50	1.71	1	1	HZ1	O
THR56	Heavy	PHE4	3.79	-0.44	0	0	OG1	HE2
TYR109	Heavy	HIS6	3.87	-1.63	0	0	HA	HE2
GLY103	Heavy	HIS6	4.14	-0.52	0	0	HA3	HE1
TRP52	Heavy	ARG5	4.25	-0.23	0	1	HH2	HA
ARG106	Heavy	HIS6	4.75	0.20	1	0	C	HE1
ARG105	Heavy	ARG5	4.95	0.58	1	1	HH12	H
GLY107	Heavy	PHE4	4.98	-0.18	0	0	HA3	HB2
ASP54	Heavy	PHE4	5.11	-0.22	-1	0	OD2	HE2
GLY101	Heavy	HIS6	5.19	0.11	0	0	HA2	HE2
TYR59	Heavy	ALA2	5.21	-0.08	0	1	HH	O
TRP52	Heavy	HIS6	5.31	-0.32	0	0	HH2	H
ARG105	Heavy	ALA2	5.39	0.99	1	1	HH21	HB2
PRO108	Heavy	ARG5	5.49	-0.33	0	1	HD3	HA
PRO108	Heavy	PHE4	5.72	-0.16	0	0	HD3	O
GLY103	Heavy	ASP7	5.74	-0.27	0	-2	HA3	H
ARG105	Heavy	ASP7	5.78	-2.43	1	-2	HH12	OD2
TYR59	Heavy	ARG5	5.81	0.03	0	1	HE2	N
GLY107	Heavy	ARG5	5.85	-0.03	0	1	HA3	HA
LYS58	Heavy	GLU3	6.05	-0.82	1	-1	H	OE1
ALA104	Heavy	ASP7	6.18	-0.36	0	-2	H	OD1
ARG105	Heavy	HIS6	6.31	-0.31	1	0	C	HE1
ASP99	Heavy	HIS6	6.33	-0.24	-1	0	OD1	HE2
ALA104	Heavy	HIS6	6.41	-0.10	0	0	H	HE1
ILE102	Heavy	ASP7	6.48	-0.06	0	-2	HG23	H
ARG106	Heavy	PHE4	6.61	-0.17	1	0	N	HB2
TRP52	Heavy	GLU3	6.68	-0.32	0	-1	HH2	C
PHE53	Heavy	PHE4	6.71	-0.12	0	0	H	HD2
TYR110	Heavy	HIS6	6.87	-0.09	0	0	N	HE2
ARG105	Heavy	GLU3	6.90	-1.33	1	-1	HE	C
VAL50	Heavy	PHE4	7.13	-0.04	0	0	HG13	HD2
LYS65	Heavy	GLU3	7.23	-0.98	1	-1	HZ3	HG3
LYS58	Heavy	PHE4	7.33	-0.18	1	0	N	HD2
ILE102	Heavy	PHE4	7.36	-0.04	0	0	O	HB2
HIS35	Heavy	HIS6	7.43	0.00	0	0	HE1	HE2
LYS57	Heavy	ARG5	7.52	0.71	1	1	HE3	H
GLY107	Heavy	ASP7	7.54	0.00	0	-2	HA3	H
ALA104	Heavy	PHE4	7.62	-0.04	0	0	HB2	HB2
GLY55	Heavy	PHE4	7.68	-0.01	0	0	H	HE2
GLY103	Heavy	PHE4	7.69	-0.01	0	0	HA3	HB2
GLY103	Heavy	ARG5	7.78	-0.05	0	1	HA3	O
THR56	Heavy	GLU3	7.84	-0.13	0	-1	HB	OE1
ARG100	Heavy	HIS6	7.87	0.07	1	0	HH22	HE1
TYR32	Light	ASP7	1.80	-9.45	0	-2	HH	OXT
TYR92	Light	ASP7	1.82	-6.45	0	-2	HH	O
TYR92	Light	HIS6	2.25	-3.45	0	0	HD1	HB3

TYR91	Light	HIS6	2.58	-1.46	0	0	O	HD2
TYR32	Light	HIS6	2.76	-6.05	0	0	OH	HB2
SER93	Light	ARG5	3.13	-2.13	0	1	HA	HB3
TYR92	Light	ARG5	3.60	-4.25	0	1	O	HB3
SER93	Light	HIS6	4.17	-0.73	0	0	HA	H
THR94	Light	ARG5	4.62	-0.84	0	1	H	HB3
SER30	Light	ASP7	4.71	-0.75	0	-2	HG	OXT
THR94	Light	PHE4	5.20	-0.21	0	0	OG1	O
THR94	Light	GLU3	5.38	-0.11	0	-1	HG1	HB2
LEU96	Light	HIS6	5.56	-0.12	0	0	HD22	HD2
SER93	Light	PHE4	5.58	-0.15	0	0	HA	O
TYR91	Light	ARG5	5.85	-0.19	0	1	O	HA
SER30	Light	HIS6	5.85	-0.08	0	0	HB3	HB2
THR94	Light	HIS6	6.09	-0.11	0	0	H	H
ILE29	Light	HIS6	6.18	-0.17	0	0	O	HB3
ILE2	Light	ARG5	6.21	-0.02	0	1	HD11	HH11
TYR92	Light	PHE4	6.30	-0.06	0	0	O	O
GLN90	Light	HIS6	6.45	-0.13	0	0	C	HD2
SER31	Light	ASP7	6.56	-0.38	0	-2	H	OXT
LEU96	Light	ARG5	6.61	-0.04	0	1	HD21	HA
TYR32	Light	ARG5	6.67	-0.30	0	1	HH	O
SER28	Light	ARG5	6.68	-0.14	0	1	O	HH12
ILE29	Light	ASP7	6.81	-0.05	0	-2	O	OXT
LEU96	Light	PHE4	7.05	-0.03	0	0	HD21	O
ILE29	Light	ARG5	7.12	-0.06	0	1	HA	HH12
LEU33	Light	HIS6	7.30	-0.09	0	0	N	HD2
GLN27	Light	ARG5	7.43	0.02	0	1	HB2	HH11
SER28	Light	ASP7	7.52	0.11	0	-2	HG	O
SER31	Light	HIS6	7.52	-0.10	0	0	H	HB2
TYR91	Light	PHE4	7.52	-0.03	0	0	O	O
ASN34	Light	HIS6	7.86	-0.03	0	0	HD22	HE2
SER93	Light	GLU3	7.92	-0.10	0	-1	HA	O
TYR91	Light	ASP7	7.95	-0.02	0	-2	O	N
ALA50	Light	HIS6	7.96	-0.01	0	0	HB3	HE2

Table S28 – Quantum biochemistry details of RC MD6-#7

**Adu Residue**	**Adu Chain**	**Aβ Residue**	**Distance (Å)**	**Interaction Energy (kcal/mol)**	**Adu Residue Charge**	**Aβ Residue Charge**	**Adu Atom**	**Aβ Atom**
PRO108	Heavy	HIS6	2.00	-5.28	0	0	O	HE2
GLY107	Heavy	PHE4	2.36	-0.52	0	0	HA3	HE1
ARG105	Heavy	PHE4	2.52	-1.96	1	0	HB3	HZ
GLY107	Heavy	HIS6	2.59	-1.58	0	0	O	HE1
PRO108	Heavy	PHE4	2.65	-1.38	0	0	HD3	HE1
TYR109	Heavy	HIS6	2.72	-2.29	0	0	HB3	HE2
ILE102	Heavy	HIS6	2.83	-1.95	0	0	HG22	HE1
TRP52	Heavy	PHE4	2.99	-3.10	0	0	HZ2	HZ

TYR59	Heavy	PHE4	3.18	-3.36	0	0	CE2	HB3
LYS57	Heavy	PHE4	3.32	-0.97	1	0	HE2	HE2
ARG106	Heavy	HIS6	4.14	-0.57	1	0	C	HE1
ARG105	Heavy	ASP7	4.21	-3.48	1	-2	HG2	OD1
GLY101	Heavy	HIS6	4.68	-0.28	0	0	HA2	HE1
GLY103	Heavy	HIS6	4.69	-0.09	0	0	HA3	HE1
ALA104	Heavy	ASP7	4.75	-0.84	0	-2	HB2	OD1
ARG105	Heavy	HIS6	4.90	-0.53	1	0	HB3	HA
ARG106	Heavy	PHE4	5.02	0.32	1	0	N	HZ
TYR59	Heavy	ALA2	5.11	-0.16	0	1	HE1	O
LYS65	Heavy	GLU3	5.30	-1.40	1	-1	HZ1	HG3
TRP52	Heavy	HIS6	5.46	-0.16	0	0	HZ2	HE1
ARG105	Heavy	ARG5	5.49	0.73	1	1	HG3	O
TYR59	Heavy	GLU3	5.52	-0.44	0	-1	HE1	HA
GLY103	Heavy	ASP7	5.53	-0.46	0	-2	HA3	H
VAL50	Heavy	PHE4	5.63	-0.15	0	0	HG12	HD1
ILE102	Heavy	PHE4	5.74	-0.11	0	0	O	HZ
ASP62	Heavy	GLU3	6.25	1.31	-1	-1	OD2	HG3
ALA104	Heavy	HIS6	6.36	-0.14	0	0	H	ND1
GLY107	Heavy	ARG5	6.39	-0.11	0	1	HA3	C
ILE102	Heavy	ASP7	6.47	-0.09	0	-2	HG23	O
ALA104	Heavy	PHE4	6.47	0.03	0	0	C	HZ
PRO108	Heavy	ARG5	6.48	-0.31	0	1	HD3	HA
TYR110	Heavy	HIS6	6.48	-0.08	0	0	H	HE2
TRP47	Heavy	PHE4	6.49	-0.07	0	0	HH2	HB2
ASP99	Heavy	HIS6	6.58	-0.23	-1	0	OD2	HE2
GLY103	Heavy	PHE4	6.64	-0.03	0	0	HA3	HZ
TRP52	Heavy	ARG5	6.67	-0.09	0	1	HH2	H
GLY107	Heavy	ASP7	6.72	0.01	0	-2	H	H
LYS57	Heavy	ARG5	6.76	0.71	1	1	HE2	H
TYR59	Heavy	ARG5	6.84	-0.20	0	1	HE2	H
TYR109	Heavy	PHE4	6.89	-0.08	0	0	HB3	HE1
GLY101	Heavy	PHE4	7.10	0.00	0	0	HA2	HE1
ARG106	Heavy	ASP7	7.20	-1.44	1	-2	H	H
TYR60	Heavy	GLU3	7.33	0.09	0	-1	O	HG3
LYS58	Heavy	PHE4	7.34	-0.08	1	0	O	HB3
TRP47	Heavy	GLU3	7.37	0.03	0	-1	HZ3	HG2
LYS65	Heavy	ALA2	7.49	1.08	1	1	HZ1	H2
LYS65	Heavy	PHE4	7.61	-0.07	1	0	HZ1	H
HIS35	Heavy	HIS6	7.62	0.00	0	0	HE2	HE2
ARG100	Heavy	HIS6	7.66	0.00	1	0	O	HE1
HIS35	Heavy	PHE4	7.70	-0.02	0	0	HE1	HD1
LYS57	Heavy	ALA2	7.78	0.87	1	1	HE3	O
THR61	Heavy	GLU3	7.78	0.02	0	-1	HA	HG2
TYR60	Heavy	PHE4	7.84	-0.05	0	0	H	H
SER93	Light	PHE4	1.89	-7.06	0	0	HG	O
TYR92	Light	HIS6	1.97	-5.24	0	0	O	H
SER93	Light	ARG5	2.15	-5.75	0	1	HB2	HG3

THR94	Light	PHE4	2.52	-2.90	0	0	H	O
TYR92	Light	ARG5	2.56	-10.28	0	1	O	HA
TYR32	Light	HIS6	2.56	-4.28	0	0	HH	HB2
THR94	Light	GLU3	2.69	-1.34	0	-1	O	HB2
TYR91	Light	HIS6	2.90	-0.58	0	0	O	HD2
PRO95	Light	GLU3	2.91	-0.67	0	-1	HD2	HG2
SER93	Light	GLU3	3.27	-0.37	0	-1	HG	HB2
SER93	Light	HIS6	3.59	-1.84	0	0	HA	H
THR94	Light	ARG5	3.65	-0.83	0	1	H	HA
TYR32	Light	ASP7	3.70	-0.35	0	-2	HH	O
ASP1	Light	GLU3	4.07	-0.29	0	-1	H3	OE1
LEU96	Light	PHE4	4.42	-0.26	0	0	HD22	HD1
TYR92	Light	ASP7	4.48	0.11	0	-2	HE1	OXT
TYR92	Light	PHE4	4.82	-1.43	0	0	O	O
ILE2	Light	ARG5	4.83	-0.09	0	1	HD13	HG3
TYR91	Light	PHE4	5.02	-0.25	0	0	O	HE1
TYR91	Light	ARG5	5.12	-0.42	0	1	O	HA
THR94	Light	HIS6	5.40	-0.13	0	0	H	H
SER30	Light	HIS6	5.93	-0.10	0	0	HB3	HB3
PRO95	Light	PHE4	6.01	0.03	0	0	N	O
GLN27	Light	ARG5	6.23	0.08	0	1	HE21	HD2
THR94	Light	ALA2	6.34	-0.44	0	1	HG1	O
ILE29	Light	ARG5	6.38	-0.08	0	1	HA	HG3
LEU96	Light	HIS6	6.41	-0.07	0	0	HD22	HE2
SER28	Light	ARG5	6.42	-0.19	0	1	O	HD2
ILE29	Light	HIS6	6.45	-0.17	0	0	O	HD2
TYR32	Light	ARG5	6.52	-0.20	0	1	HE1	HG2
LEU96	Light	ARG5	6.63	-0.06	0	1	HD21	HA
ILE2	Light	GLU3	6.64	-0.08	0	-1	H	HG2
GLN90	Light	ARG5	6.75	-0.11	0	1	HE22	HA
GLN90	Light	PHE4	6.79	-0.06	0	0	HE22	O
GLN90	Light	HIS6	6.93	-0.12	0	0	OE1	HD2
ILE2	Light	PHE4	7.02	-0.04	0	0	HD13	O
SER30	Light	ASP7	7.17	-0.15	0	-2	HG	OXT
ILE2	Light	HIS6	7.17	-0.10	0	0	HD13	H
SER31	Light	HIS6	7.20	-0.14	0	0	H	HD2
SER93	Light	ALA2	7.24	-0.01	0	1	HG	C
GLN90	Light	GLU3	7.30	-0.04	0	-1	HE22	HB2
TYR92	Light	GLU3	7.47	-0.03	0	-1	O	HB2
TYR32	Light	PHE4	7.52	-0.06	0	0	HE2	HE1
LEU96	Light	GLU3	7.54	-0.06	0	-1	HD21	HB2
ASN34	Light	HIS6	7.57	-0.01	0	0	HD22	HE2
LEU33	Light	HIS6	7.63	-0.05	0	0	N	HD2
SER30	Light	ARG5	7.66	0.00	0	1	HB3	HG2
PRO95	Light	ARG5	7.69	-0.14	0	1	N	HA
SER93	Light	ASP7	7.85	0.02	0	-2	HB2	N
ASP1	Light	PHE4	7.89	-0.07	0	0	HA	O
PRO95	Light	ALA2	7.92	-0.23	0	1	HD2	C

TYR91	Light	ASP7	7.98	0.16	0	-2	O	H

Table S29 – Quantum description of energetic hot spots based on representative conformations.

		Adu-Residue	MD2-#0	MD2-#1	MD2-#2	MD2-#3	MD2-#4	MD2-#5	MD2-#6	MD2-#7	MD2-#8	MD6-#0	MD6-#1	MD6-#2	MD6-#3	MD6-#4	MD6-#5	MD6-#6	MD6-#7
HC Resdues		VAL50	-0.18	-0.25	-0.26	-0.16	-0.23	-0.19	-0.20	-0.15	-0.19	-0.03	-0.05	-0.05	-0.04	-0.31	-0.03	-0.05	-0.17
HC Resdues	H2	TRP52	-3.67	-3.17	-3.80	-3.08	-3.98	-3.97	-3.41	-4.11	-4.25	-3.63	-2.83	-2.85	-5.15	-3.92	-2.02	-5.99	-3.46
HC Resdues	H2	LYS57	-0.09	-0.89	-0.75	-1.71	-0.96	-1.15	0.31	-0.75	-0.57	-11.78	-2.73	-1.91	-14.74	-1.56	-1.95	-14.26	-2.15
HC Resdues	H2	LYS58	-0.98	-1.06	-1.26	-0.40	-1.18	-0.59	-0.50	-1.31	-1.16	-1.31	-0.10	-0.12	-0.69	-0.21	-0.07	-1.00	-0.10
HC Resdues	H2	TYR59	-7.51	-7.86	-8.32	-7.38	-8.09	-7.70	-7.16	-8.00	-5.63	-14.59	-1.90	-2.51	-17.60	-7.88	-1.65	-15.25	-4.18
HC Resdues	H2	TYR60	-0.18	0.06	0.40	3.19	0.21	1.54	1.09	0.00	-0.29	0.10	-0.01	-0.02	0.02	-0.38	0.14	0.10	-0.11
HC Resdues		THR61	0.11	0.12	0.15	0.63	0.18	0.02	-0.08	0.03	-0.01	0.00	0.00	0.00	0.00	-0.20	0.06	0.00	0.01
HC Resdues		ASP62	0.41	1.05	1.13	1.13	0.23	1.33	1.61	1.63	0.55	0.00	0.97	1.17	0.00	0.58	1.18	0.00	0.40
HC Resdues		LYS65	-9.15	-12.78	-9.11	-10.77	-9.46	-9.51	-10.49	-10.22	-12.38	0.00	0.00	-0.01	-1.25	-0.68	-1.06	-0.98	-0.40
HC Resdues		ASP99	-0.27	-0.29	-0.33	-0.32	-0.21	-0.30	-0.29	-0.24	-0.22	-0.24	-0.24	-0.24	-0.18	-0.20	-0.23	-0.24	-0.27
HC Resdues		ARG100	-0.02	-0.02	0.00	0.03	-0.06	0.00	0.00	-0.02	-0.03	-0.01	0.02	0.05	0.03	0.01	0.02	0.07	0.00
HC Resdues		GLY101	-0.23	-0.19	0.14	-0.17	-0.06	0.10	-0.11	-0.10	0.27	0.05	-0.29	-0.05	-0.32	-0.22	0.01	0.12	-0.28
HC Resdues	H3	ILE102	-2.02	-1.35	-1.56	-0.99	-2.44	-0.37	-1.52	-1.77	-1.06	-0.54	-2.02	-1.59	-1.54	-2.30	-1.74	-2.58	-2.18
HC Resdues	H3	GLY103	-0.47	-0.54	-0.32	-1.11	-0.11	-0.53	-1.17	-0.42	-1.20	-1.70	-0.97	-0.71	-0.81	-1.39	-0.39	-0.84	-0.56
HC Resdues	H3	ALA104	-0.48	-0.42	-0.37	-0.44	0.01	-1.07	-0.89	-0.46	-0.96	-0.80	-1.86	-0.94	-0.19	-1.09	-0.82	-0.48	-0.87
HC Resdues	H3	ARG105	-6.38	-4.40	-22.31	-14.92	-5.42	-16.95	-4.91	-16.86	-5.25	-8.81	-25.64	-23.61	-10.86	-19.13	-22.48	-7.20	-5.85
HC Resdues	H3	ARG106	-0.87	-1.59	-0.71	-1.58	0.77	-1.17	-1.02	-1.02	-1.19	-1.15	-0.66	-1.25	-2.05	-1.58	-1.18	-0.78	-1.14
HC Resdues	H3	GLY107	-2.74	-2.97	-2.17	-4.04	-1.55	-4.46	-3.10	-2.03	-2.73	-2.62	-1.04	-2.34	-1.88	-2.97	-1.97	-2.28	-2.20
HC Resdues	H3	PRO108	-4.73	-5.20	-7.60	-6.79	-7.07	-6.74	-7.06	-5.40	-6.31	-5.46	-4.50	-5.36	-4.85	-5.51	-5.84	-5.72	-6.51
HC Resdues	H3	TYR109	-2.49	-2.36	-2.33	-3.29	-2.42	-2.16	-1.70	-2.46	-2.13	-2.13	-1.51	-1.75	-1.60	-2.14	-2.01	-1.63	-2.32
HC Resdues		TYR110	-0.19	-0.32	-0.17	-0.14	-0.13	-0.18	-0.12	-0.14	-0.13	-0.08	-0.09	-0.13	-0.07	-0.12	-0.10	-0.09	-0.09
LC Residues		ASP1	-4.73	-14.27	-11.70	0.34	-9.32	-0.17	0.08	-0.26	-0.57	0.00	0.62	-0.55	0.18	-0.22	-0.59	0.09	-0.22
LC Residues		ILE2	-0.63	-0.49	-0.49	-0.21	-0.58	-0.18	-0.18	-0.29	-0.37	-0.02	-0.24	-0.40	-0.10	-0.17	-0.34	-0.04	-0.30

	SER28	0.07	-0.34	-0.09	-0.19	-0.23	-0.09	-0.06	-0.25	-0.27	0.24	-0.15	-0.06	-0.04	-0.38	0.10	-0.04	-0.03
	ILE29	-0.22	-0.26	-0.28	-0.45	-0.35	-0.21	-0.33	-0.21	-0.25	-0.08	-0.21	-0.33	-0.29	-0.22	-0.18	-0.28	-0.21
	SER30	-0.24	-0.16	-0.22	-0.81	-0.16	-0.25	-0.30	-0.24	-0.38	-0.57	-0.59	-0.82	-0.57	-0.24	-0.27	-0.86	-0.25
	SER31	-0.20	-0.12	-0.15	-0.30	-0.18	-0.22	-0.28	-0.20	-0.17	-0.39	-0.34	-0.47	-0.38	-0.23	-0.47	-0.48	-0.17
	TYR32	-5.94	-4.56	-5.48	-4.92	-5.22	-5.03	-4.61	-3.76	-4.92	-13.99	-13.32	-14.08	-14.84	-4.50	-13.85	-15.79	-4.88
	ASN34	-0.02	-0.04	-0.03	-0.03	-0.02	0.00	-0.01	-0.02	-0.01	-0.02	-0.04	-0.03	-0.04	-0.03	-0.04	-0.03	-0.01
	GLN90	-0.29	-0.43	-0.35	-0.29	-0.22	-0.32	-0.36	-0.28	-0.34	-0.17	-0.19	-0.25	-0.13	-0.31	-0.15	-0.17	-0.33
L3	SER91/ TYR91	-0.42	-0.21	-0.50	-0.97	-0.56	-0.67	-0.83	-0.92	-1.19	-1.93	-1.22	-2.02	-1.87	-0.42	-1.97	-1.69	-1.10
L3	TYR92	-13.45	-15.59	-14.44	-19.70	-21.31	-17.59	-15.24	-17.57	-9.84	-1.02	-8.13	-9.30	-8.74	-17.93	-5.08	-14.11	-16.87
L3	SER93	-9.91	-11.44	-10.73	-10.23	-11.55	-8.42	-7.98	-10.73	-7.75	-0.75	-8.19	-9.23	-3.69	-10.49	-14.69	-2.92	-15.00
L3	THR94	-7.80	-8.07	-10.37	-20.79	-10.97	-19.51	-20.90	-10.81	-9.43	-0.48	-2.35	-1.41	-1.09	-10.42	-2.19	-1.34	-5.45
L3	PRO95	-1.14	-1.69	-1.36	-2.16	-1.55	-1.20	-1.69	-1.45	-1.64	-0.08	0.30	0.06	-0.20	-0.60	0.91	-0.25	-1.01
	LEU96	-0.61	-0.72	-0.76	-0.48	-0.75	-0.79	-0.59	-0.84	-0.72	-0.17	-0.19	-0.25	-0.16	-0.33	-0.18	-0.21	-0.43
